# Hyperagonism of a Vitamin D Receptor Agonist/Histone Deacetylase Inhibitor Hybrid Molecule

**DOI:** 10.1021/acs.jmedchem.5c01932

**Published:** 2025-09-18

**Authors:** Fatemeh Sarmadi, Amelia Caza, Zhizhong Gao, Natacha Rochel, James L. Gleason, John H. White

**Affiliations:** † Department of Physiology, 5620 McGill University, 3655 Promenade Sir William Osler, Montreal, QC H3G 1Y6, Canada; ‡ Department of Chemistry, 5620 McGill University, 801 Sherbrooke W., Montreal, QC H3A 0B8, Canada; § Department of Integrative and Structural Biology, Institut de Génétique et de Biologie Moléculaire et Cellulaire, Université de Strasbourg, CNRS, Inserm, UMR 7104-UMR-S 1258, F-67400 Illkirch, France; ∥ Department of Medicine, 5620 McGill University, 3655 Promenade Sir William Osler, Montreal, QC H3G 1Y6, Canada

## Abstract

1,25-Dihydroxyvitamin D (1,25D) analogs engage the vitamin D receptor (VDR) and can exert anticancer and immunomodulatory effects. Although tumors often resist 1,25D monotherapy, combining VDR agonism with histone deacetylase inhibitors (HDACi) restores anticancer efficacy. Here, we present AC-340, a novel bifunctional molecule that incorporates HDACi into a VDR agonist backbone. Besides its robust bifunctionality in vitro in multiple melanoma models, RNaseq analysis of B16–F10 mouse melanoma cells revealed that AC-340 superinduces the expression of a broad array of VDR target genes. Comparative structural studies and ChIP-qPCR revealed that AC-340 forms more interactions than 1,25D with residues in the VDR coactivator binding domain, leading to more efficacious recruitment of coactivator CBP. This, likely coupled with AC-340 HDACi activity, leads to elevated H3K27 acetylation of VDR target genes, a mark of active transcription. Thus, AC-340 functions as a VDR hyperagonist and should be efficacious in mono- or combination therapies against multiple cancer models.

## Graphical abstract

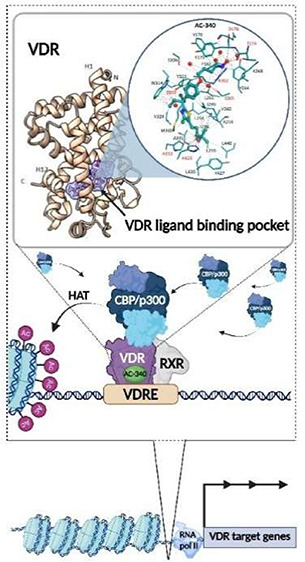

## Introduction

The hormonal form of vitamin D, 1α,25-dihydroxyvitamin D (1,25D; Figure 1A, 1), is an agonist of the nuclear VDR, a ligand-regulated transcription factor.^1–3^ Vitamin D is obtained from limited dietary sources, supplements, or by cutaneous photochemical and thermal conversion of 7-dehydrocholesterol in the presence of adequate solar UVB irradiation.^4^ 1,25D is produced from circulating vitamin D by largely hepatic hydroxylation at the 25-position to produce 25-hydroxyvitamin D (25D), followed by highly and tissue-specifically regulated 1α-hydroxylation catalyzed by CYP27B1.^5^ While its regulation was first characterized in the kidney, CYP27B1 is now known to be expressed in several nonrenal cell types, including throughout the immune system.^5^ Vitamin D signaling is self-attenuating via VDR-driven induction of the gene encoding CYP24A1, which initiates catabolic degradation of 1,25D and 25D.^6^ Vitamin D was initially discovered for its critical role in calcium homeostasis^7,8^ but it is now known to have pleiotropic actions in multiple organs, notably in the immune system.^9^ Accordingly, the VDR is widely expressed, and a combination of gene expression profiling and chromatin immunoprecipitation followed by high-throughput sequencing (ChIPseq) studies have shown that the VDR regulates the transcription of several genes controlling the cell cycle, differentiation and immunity.^10–12^ These and other experiments provide a mechanistic basis for the potential of 1,25D and its analogs as therapeutics in proliferative disorders, such as cancer and psoriasis, as well as in immune system regulation.^13–17^

**Figure 1:**
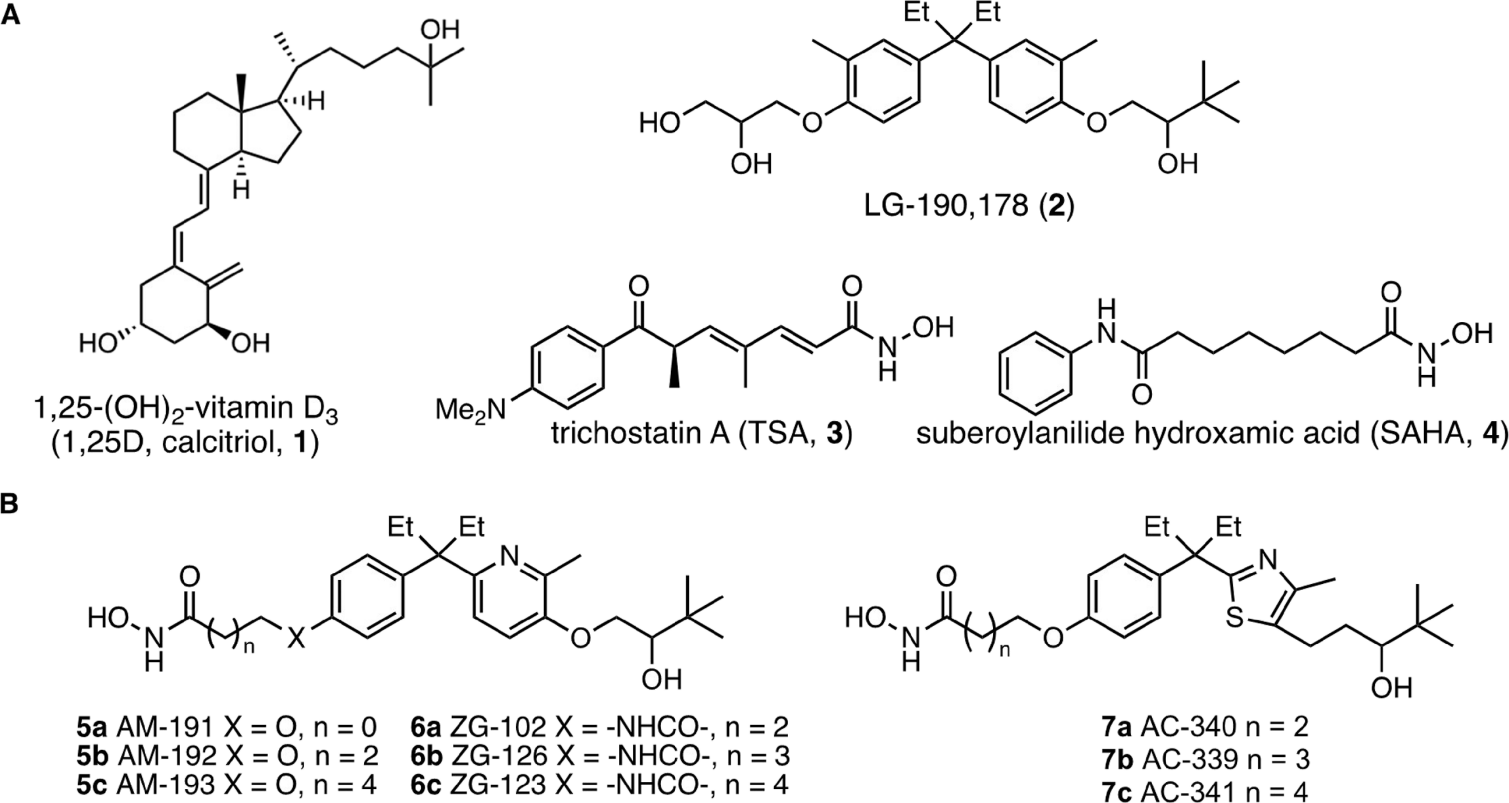
(A) Structures of 1,25D (1), nonsecosteroidal VDR agonist LG190178 (2), HDACi’s TSA (3), and SAHA (4). (B) Structure of AM, ZG, and the newly synthesized AC series of hybrid molecules.

While there is molecular and epidemiological evidence for a role of vitamin D signaling in cancer prevention,^18–21^ 1,25D and its analogs have failed as monotherapies due to acquired tumor resistance, even though vitamin D signaling remains active. However, resistant cells are sensitive to 1,25D combined with HDACi.^22–25^ As most HDACs are zinc metalloenzymes, HDACi are generally composed of a zinc chelating group, usually a hydroxamic acid, linked via an aliphatic chain to a highly variable cap group structure.^26,27^ The combinatorial effects of 1,25D (analogs) and HDACi led us to develop bifunctional molecules that incorporate HDACi activity into the backbone of VDR agonists,^28–31^ thus acting as a form of combination therapy in a single integrated molecule. The initial hybrid design was based on the secosteroidal backbone of 1,25D. However, given the complexity of secosteroid synthesis, we turned to hybrid designs based on more easily assembled nonsecosteroidal VDR agonist LG-190178 (Figure 1A, 2). In prototypical HDACi such as tricostatin A (TSA; Figure 1A, 3) or suberoylanilide hydroxamic acid (SAHA; Figure 1A, 4), hydroxamic acids are linked to aromatic cap structures. In nonsecosteroidal hybrids, the 1α-hydroxy surrogate of LG-190178 was replaced by an aliphatic chain bearing a hydroxamic acid, with the rest of the structure serving as the cap. To optimize HDACi potency and reduce the hydrophobicity of the LG-190178 diarylpentane core, the methyl group in the aryl ring adjacent to the hydroxamic acid-bearing chain was removed, and pyridine rings were introduced into the diarylpentane core. This generated AM-193 and ZG-126 (Figure 1B),^32,33^ which possess, respectively, ether and amide linkages to the chain containing the hydroxamic acid. ZG and AM hybrids are bioavailable and act as efficacious antitumor agents in the B16–F10 mouse model of melanoma, and the mouse triple-negative breast cancer model 4T1.^33^

Among the many vitamin D analogs prepared over the years, several have been described as superagonists.^34–36^ This designation has been applied to ligands that promote gene transcription at levels higher than 1,25D at equivalent concentrations. In most cases, the source of this superagonism was higher potency for the VDR, as transcription levels became equal at saturating concentrations of 1,25D and analog.^34–36^ Here, we describe a different type of superagonism that we term hyperagonism to differentiate it from molecules that appear simply more potent. We report a 1,25D/HDACi hybrid, AC-340, which superinduces the expression of the well-characterized VDR target gene *CYP24A1* in multiple human and mouse cell lines. Notably, the levels of expression are several-fold higher than those achieved with saturating concentrations of 1,25D. Further investigation by gene expression profiling (RNaseq) showed that hyperagonism of hybrids is not unique to AC-340, although AC-340 is generally the most efficacious of compounds tested. Based on structural and mechanistic studies, we conclude that hyperagonism arises in part from an enhanced capacity of the AC-340-bound VDR to recruit coactivators. This, likely combined with the HDACi activity of AC-340, leads to a substantially elevated H3K27 acetylation at VDR target genes, a mark of active transcription.

## Results and Discussion

### Synthesis of AC Compounds

While numerous 3,3-diphenylpentane analogs of LG-190,178 have been investigated,^37^ only a few examples of 5-membered ring heterocycle analogs have been reported. These have mainly focused on thiophene and pyrrole analogs, replacing the aromatic bearing the A-ring diol mimic.^38–40^ We previously demonstrated that in VDR agonist/HDACi hybrids, pyridines were better tolerated in the aromatic ring bearing the side-chain mimic. This generated compounds with improved solubility relative to hybrids retaining the original diarylpentane core of LG190,178 (Figure 1A, 2).^32,33^ To further explore heterocyclic hybrids of this form, we prepared a series of thiazole-containing structures using a thioamide/bromoketone coupling strategy. The synthesis of the thioamide component began with double alkylation of 4-(benzyloxy)­phenyl-acetonitrile using LDA and ethyl iodide to afford **9** in 88% yield (Scheme 1). The nitrile in **9** was hydrolyzed to the corresponding amide **10** under basic conditions in 73% yield, and the necessary thioamide **11** was then generated by treatment with Lawesson’s reagent in 25% yield. Preparation of bromoketone **17a** began with alkylation of the dimethyl hydrazone of pinacolone with 4-bromo-2-methyl-3-butene to produce ketone **13** in 37% yield. Reduction of the ketone with NaBH_4_ followed by TBS protection and ozonolysis, afforded **16** in 32% over three steps. Finally, a-halogenation with NBS afforded the key bromoketone **17a** along with its corresponding 1-bromo analog **17b**. The latter was inseparable from **17b** but could be easily removed by selective reaction with 4-chlorobenzenethiol.

**Scheme 1:**
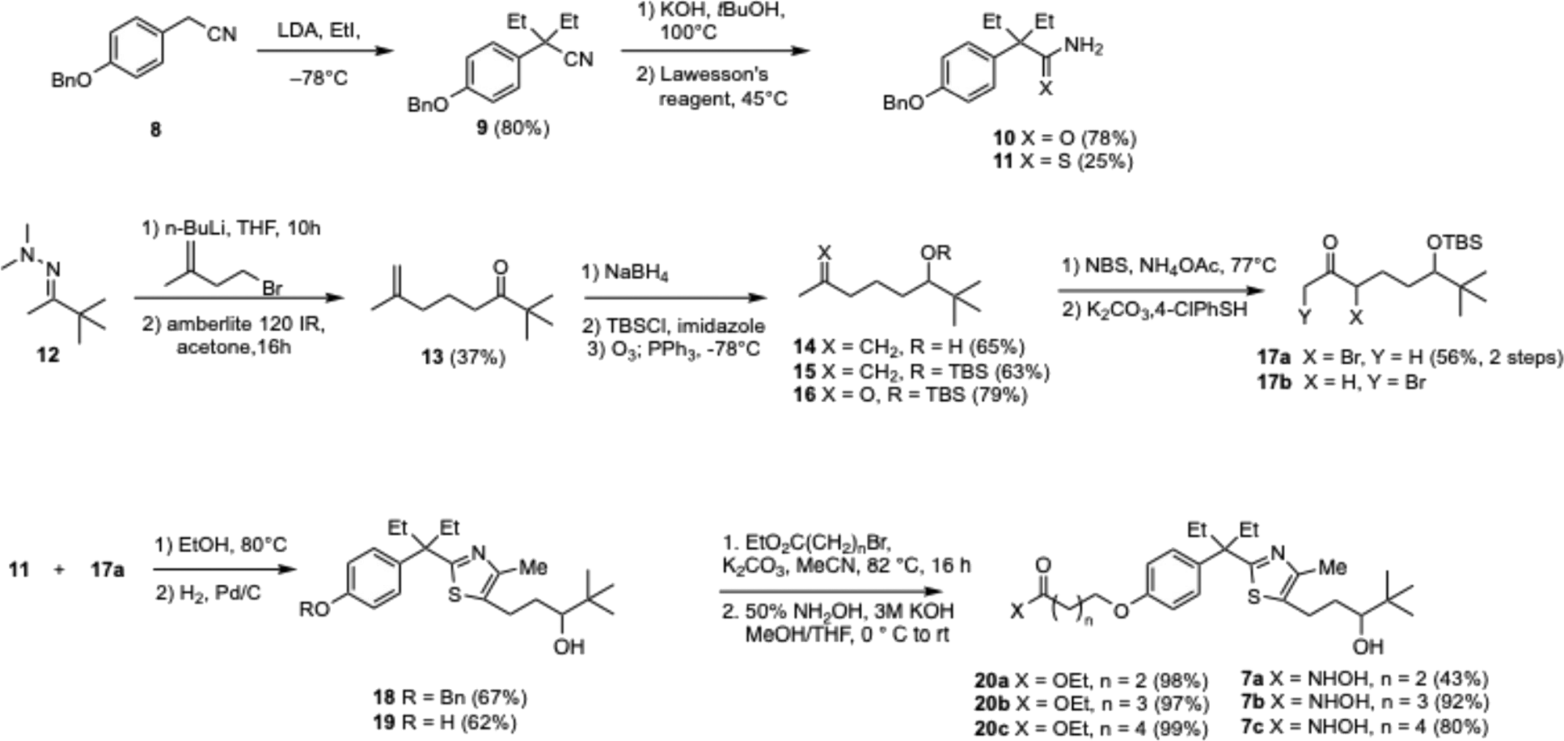
Synthesis of AC VDR Agonist/HDACi Hybrid Molecules

The thiazole was constricted as planned by direct condensation of **11** with **17a** in ethanol to afford **18** in 67% yield. Elaboration to the hybrids was achieved by hydrogenolysis of the benzyl group, in 62% yield, followed by alkylation of the resulting phenol with appropriate length bromoesters followed by condensation with hydroxylamine. This provided hybrids **7a** (AC-340), **7b** (AC-339) and **7c** (AC-341), with linking chain lengths of 3-, 4- and 5-carbons, in yields of 26–49% over the final three steps.

### Assessment of Bifunctionality of AC Compounds

AC compounds were analyzed using a battery of assays.^32,33^ VDR agonism was tested by induction of the *Cyp24a1* gene, which encodes the enzyme that initiates catabolic breakdown of 1,25D, and was used because its gene expression is exquisitely sensitive to the presence of the agonist-bound VDR. Unexpectedly, AC-340 superinduced expression of the *Cyp24a1* gene at 10 μM in mouse B16–F10 melanoma cells, unlike other hybrids (Figure 2A), suggesting it might have hyperagonist activity. In multiple experiments, *Cyp24a1* induction was >10-fold higher than that observed with 100 nM 1,25D, a saturating concentration, and several-fold higher than sister compounds AC-339 and AC-341, as well as pyridine hybrids AM-193 and ZG-126. We note that the potency of AC-340 is substantially lower than that of 1,25D. However, it is very similar to that of ZG-126, which displays VDR agonist activity in vivo at an efficacious and well-tolerated dose (ref).

**Figure 2:**
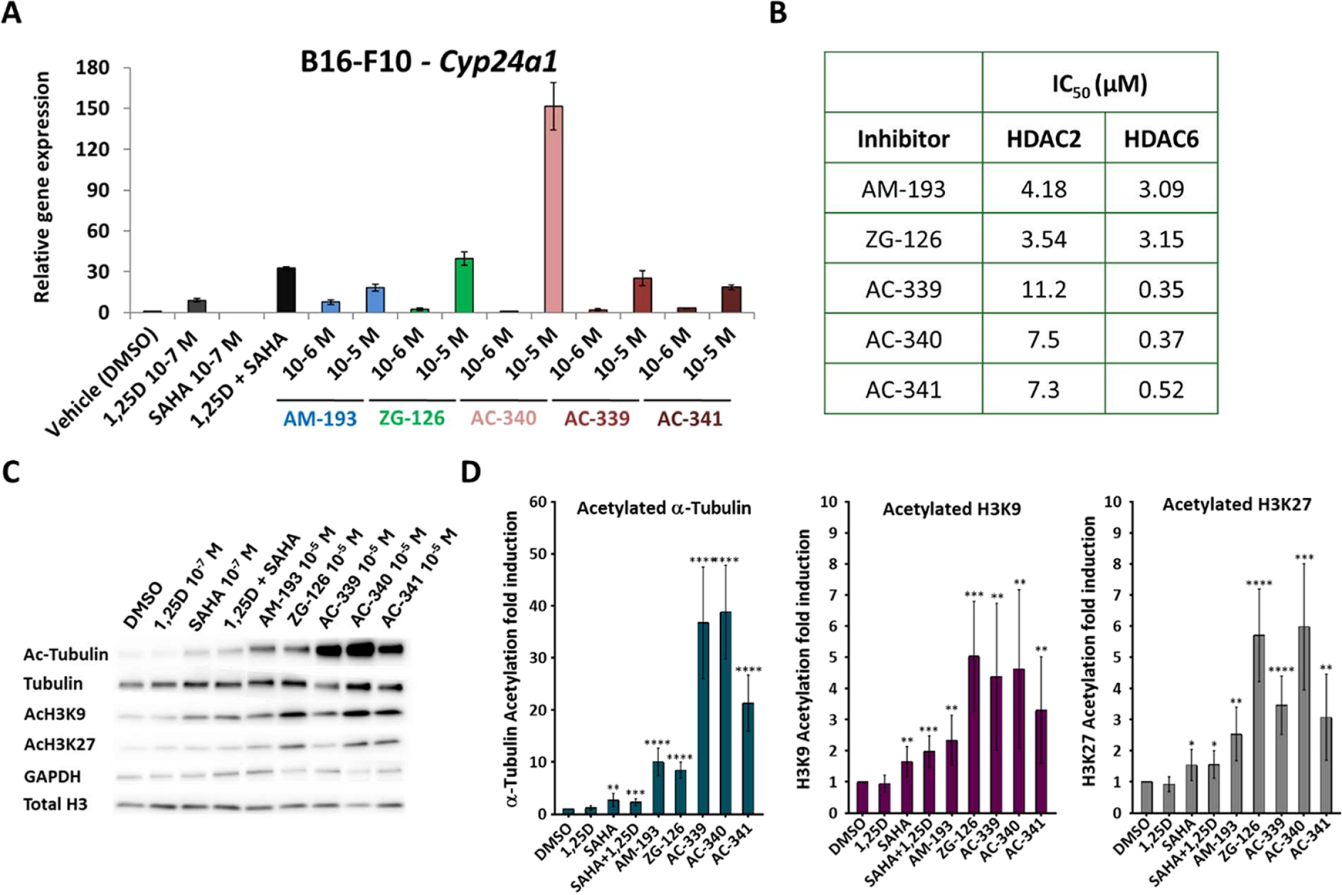
Bifunctionality assessment of AC series of compounds. (A) Analysis of *Cyp24a1* induction by AC compounds, along with 1,25D or SAHA alone or together, AM-193 and ZG-126 in B16–F10 cells. (B) IC_50_ values of AM-193, ZG-126, and AC compounds against purified HDAC2 and HDAC6 in vitro. (C) Representative Western blots showing the level of α-tubulin and histone H3 acetylation in 1,25D-, SAHA- 1,25D+SAHA-, or hybrid-treated B16–F10 lysates (D) Fold induction of α-tubulin, H3K9, and H3K27 acetylation quantified from multiple biological replicates.

We initially screened for HDACi activity of AC compounds using the class I enzyme HDAC2 and class IIb enzyme HDAC6. Their IC_50_ values for HDAC2 inhibition in vitro were slightly higher than those of AM-193 and ZG-126, whereas their IC_50_ values for HDAC6 inhibition were ∼10-fold lower (Figure 2B). Next, we analyzed by Western blotting with specific antibodies the effects of AC compounds (10 μM) on tubulin acetylation (targeted by HDAC6) and acetylation of histone H3 lysines 9 and 27 (H3K9Ac and H3K27Ac; regulated by multiple class I enzymes) (Figure 2C,D). All AC compounds induced robust protein hyperacetylation, although AC-341 was less efficacious than −339 or −340.

Because of its apparent VDR hyperagonism and robust induction of protein hyperacetylation, we decided to focus further on AC-340. Similar results in tests of the bifunctionality of AC-340 were obtained in two human melanoma cell lines, A375 and SK-MEL-28 (Figure S1). VDR hyperagonism of AC-340 was observed in both cell lines at 10 μM. Notably, ZG-126 also displayed hyperagonism in SK-MEL-28 cells. We performed an extended dose–response curve in SK-MEL-28 cells in an attempt to determine an EC50 value for AC-340 for *CYP24A1* induction. However, AC-340 induced extensive cell death at the highest concentrations tested (see Figure S1D), precluding the determination of EC50 values. (Note that extending the dose range of 1,25D to the supraphysiological concentration of 1 mM in these assays failed to enhance the induction of *CYP24A1* (Figure S1D.)

### IC_50_ Values for Inhibition of Purified HDACs 1–11 by AC-340

We assessed HDAC inhibition by AC-340 further by determining its IC_50_ for all 11 zinc metalloenzyme HDACs (i.e., class I, class II or class IV enzymes; unlike Sirtuins, class III). As a positive control, TSA was used as a nonselective inhibitor of class 1 enzymes (HDACs 1–3 and 8), class IIb HDAC 6 and class IV HDAC 11.^41^ TMP269, a selective class II inhibitor^42^ was used for class IIa HDAC4, 5, 7 and 9, whereas quisinostat was used for HDAC10 assay.^43^ IC_50_ values for AC-340 ranged from 38.9 nM for HDAC6 to very limited inhibition at concentrations higher than 100 μM for HDAC9 (Table 1). HDACs 1–3 and HDAC6, were selected for raw data demonstration in Figure 3 because they are key regulators of histone and tubulin acetylation, respectively.^44,45^ These data are in qualitative agreement with our assays above (Figure 2B) and show that AC-340 is a much more potent inhibitor of HDAC6 than HDAC2. The discrepancies in HDAC2 and HDAC6 IC_50_ values between the two sets of assays (Figure 2B and Table 1) likely arise from the differences in the peptide substrates used.

**Table 1:** IC_50_ Values for Inhibition of Purified HDACs 1-11 by AC-340

HDAC	Compound			
	AC-340	Trichostatin A	TMP269	Quisinostat
HDAC 1	1.90 × 10^−6^	2.21 × 10^−9^	ND	ND
HDAC 2	3.15 × 10^−6^	5.60 × 10^−9^	ND	ND
HDAC 3	1.07 × 10^−6^	2.26 × 10^−9^	ND	ND
HDAC 4	7.30 × 10^−5^	ND	1.08 × 10^−7^	ND
HDAC 5	9.27 × 10^−6^	ND	1.10 × 10^−7^	ND
HDAC 6	3.89 × 10^−8^	7.50 × 10^−10^	ND	ND
HDAC 7	4.51 × 10^−5^	ND	1.77 × 10^−8^	ND
HDAC 8	1.90 × 10^−6^	4.29 × 10^−67^	ND	ND
HDAC 9	>1.00 × 10^−4^	ND	1.38 × 10^−8^	ND
HDAC 10	5.91 × 10^−6^	ND	ND	4.52 × 10^−9^
HDAC 11	1.08 × 10^−6^	2.21 × 10^−6^	ND	ND

**Figure 3:**
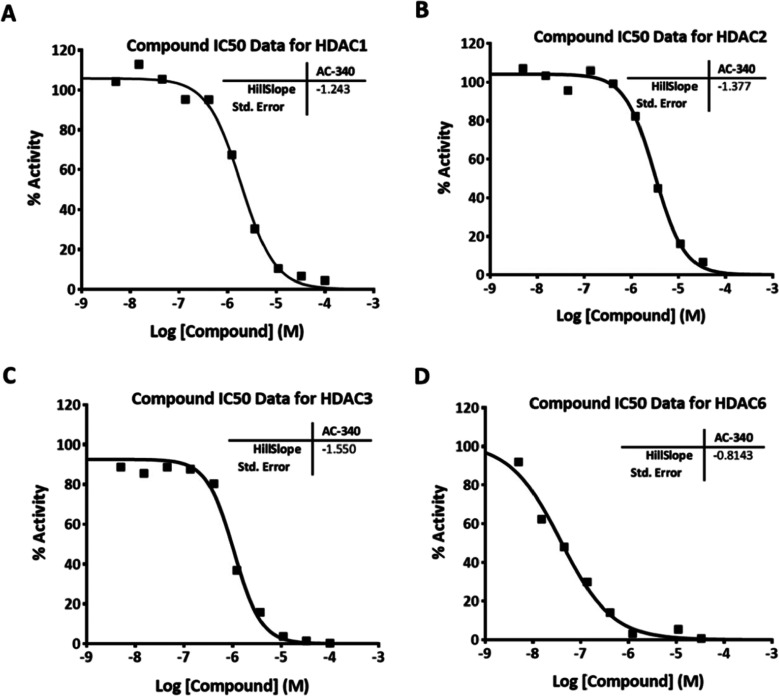
Dose–response profiles for the inhibition of (A) HDAC1, (B) HDAC2, (C) HDAC3, and (D) HDAC6 by AC-340.

### RNaseq Studies in B16–F10 Cells In Vitro

AC-340 superinduced *CYP24A1* expression in all cell lines tested, and ZG-126 appeared to be a hyperagonist in SK-MEL-28 cells. The potential for hybrid hyperagonism was probed in more depth in an RNaseq study in B16–F10 cells, in which cells were treated for 24 h under each condition (1,25D, 100 nM and SAHA, 100 nM, alone or together; hybrids 10 μM; Figure S3A). B16–F10 cells were more responsive to 1,25D+SAHA than to each compound in isolation and substantially more responsive to hybrids. AC-340 treatment produced 3 times more differentially expressed genes (DEGs) than AM-193, with ZG-126 producing an intermediate number (Figure 4A). The profiles of DEGs identified in AM-193- or ZG-126-treated cells largely overlapped with DEGs in the presence of AC-340 and those regulated by 1,25D and/or SAHA (Figure 4B). The varying effects of each treatment on the numbers of regulated genes and the fold changes observed are reflected in plots of log-fold changes and volcano plots (Figure S3B).

**Figure 4:**
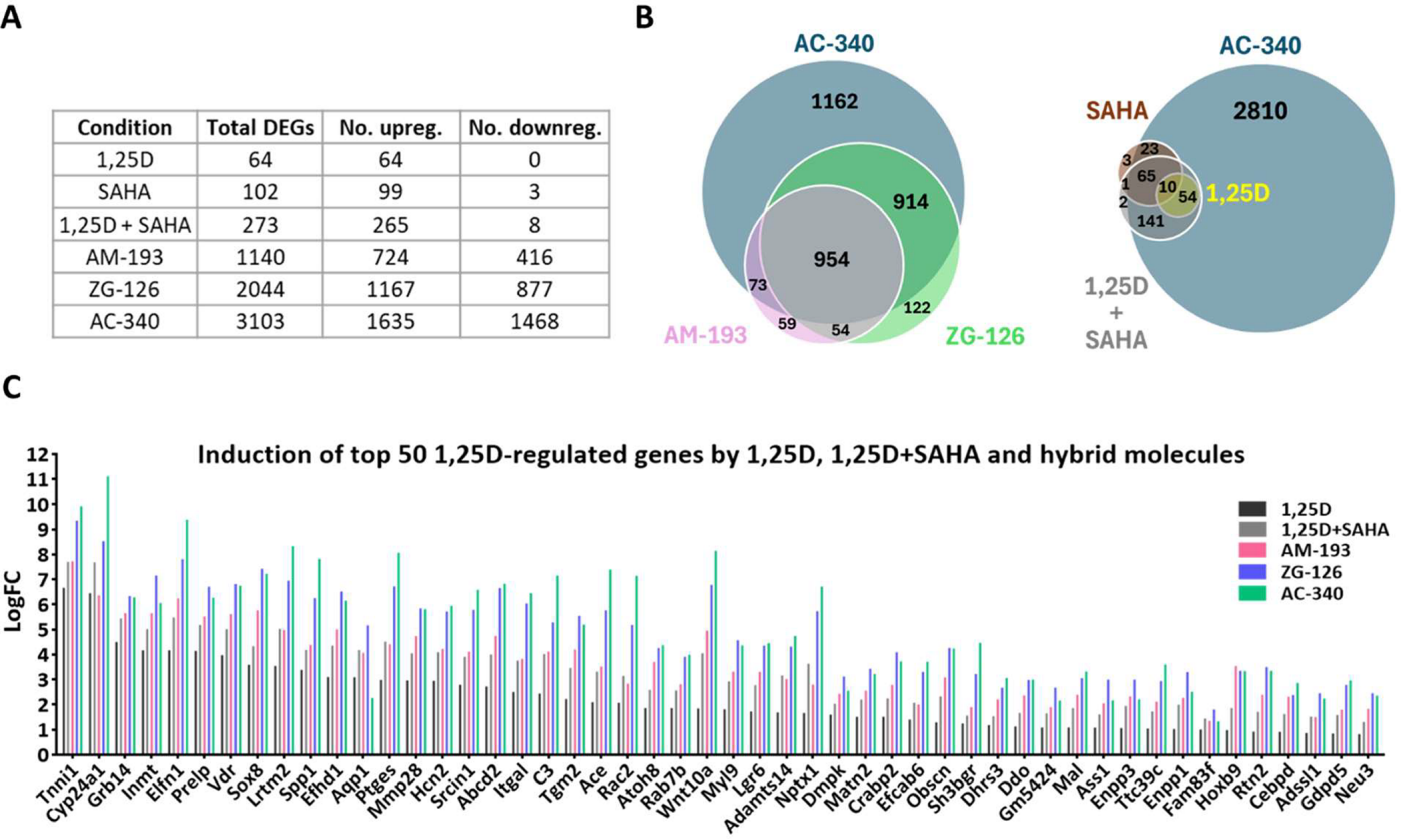
RNaseq studies in B16–F10 cells in vitro. (A) Quantity of differentially expressed genes regulated by 1,25D or SAHA alone or together, AM-193, ZG-126, and AC-340 relative to the control untreated condition. (B) Venn diagrams showing the convergence between populations of DEGs (up- or downregulated) observed in the presence of AC-340 and those regulated by 1,25D and/or SAHA, AM-193, and ZG-126. (C) Aligned fold regulations of the top 50 most induced genes by 1,25D with those observed in 1,25D+SAHA, AM-193, ZG-126, or AC-340 treatment.

The enhanced response of B16–F10 cells to AC-340 likely reflects the VDR hyperagonist activity observed in *Cyp24a1* induction assay (Figure 2A). To examine this further, we aligned the fold regulations of the top 50 most induced genes by 1,25D with those observed with 1,25D+SAHA or hybrids. In 49 of 50 cases, AC-340 superinduced their expression, often several fold (Figure 4C), consistent with AC-340 being a broad hyperagonist of 1,25D-induced genes. While superinduction by AC-340 was generally greater than that of other hybrids or 1,25D+SAHA, consistent with the relative numbers of DEGs observed under each condition, it was not universal. The relative fold inductions of *Cyp24a1* expression by 1,25D, 1,25D+SAHA and hybrids observed by RNaseq were in excellent agreement of those seen by directed RT-qPCR (Figure 2A). Notably, while the *Spp1* gene (encoding osteopontin) was most strongly regulated by AC-340, ZG-126 was the most efficacious inducer of genes encoding transglutaminase 2 (*Tgm2*) and proline and arginine rich end leucine rich repeat protein (*Prelp*) (Figure S4). The only 1,25D target gene not more strongly induced by AC-340 was the gene encoding aquaporin1 (*Aqp1*) (Figure S4). Collectively, these results show that the RNaseq data is very reliable and that AC-340 hyperagonism is widespread.

### Exploring Potential Mechanisms of AC-340 Hyperagonism

It is highly significant that AC-340 hyperagonism is observed in both mouse and human cell lines, as expression of the *Vdr* gene is autoregulated in mice but not in humans.^46^ Indeed, we found that hybrids induced much higher levels of VDR gene and protein expression than 1,25D after 24 or 6 h of treatment in B16–F10 cells (Figure 5A,B). In contrast, *VDR* gene expression was not induced in A375 and SK-MEL28 cells (and indeed suppressed in A375 cells) by 1,25D, AC-340 or other hybrids (Figure 5C,E and data not shown). However, 1,25D and AC-340 treatments led to higher VDR protein expression (suggesting protein stabilization) in A375 cells, whereas AC-340 showed no such activity in SK-MEL28 cells (Figure 5D,F). Collectively, these data show that levels of VDR mRNA or protein expression observed with 1,25D or hybrid treatments do not correlate with the degree of superinduction observed of VDR target genes, suggesting that other mechanisms control hyperagonism.

**Figure 5:**
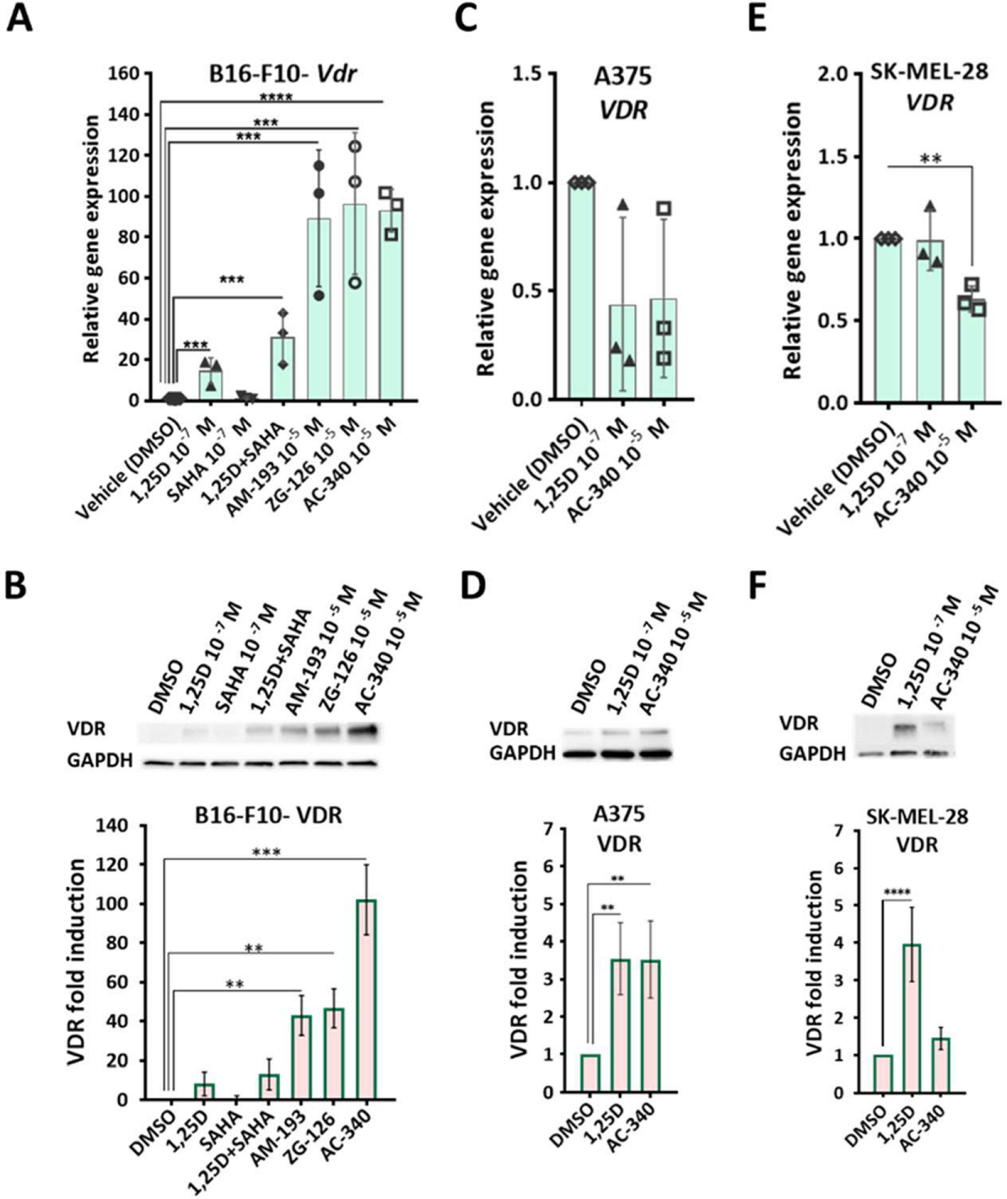
Regulation of VDR at mRNA and protein levels in mouse and human melanoma cells. (A) VDR gene and (B) protein expression in B16–F10 cells treated with 1,25D or SAHA alone or together, AM-193, ZG-126, and AC-340. (C) VDR gene and (D) protein expression in A375 cells treated with 1,25D or AC-340. (E) VDR gene and (F) protein expression in SK-MEL-28 cells under the same conditions as A375 cells.

CYP24A1 induction selectively attenuates vitamin D signaling by catalyzing catabolic degradation of 1,25D by hydroxylating the cholesterol side chain of 1,25D. In contrast, hybrids should be resistant to induced catabolism. If CYP24A1 resistance was a mechanism for hybrid hyperagonism, then blockade of CYP24A1 activity should boost the efficacy of 1,25D-regulated gene transcription to levels observed with hybrid treatment. To test this, we examined the effect of CYP inhibitor ketoconazole on the expression of three 1,25D-regulated genes. While, as expected, ketoconazole enhanced induction by 1,25D of the target genes tested, combined 1,25D/ketoconazole treatment was not as efficacious as AC-340 under any conditions (Figure 6A). Therefore, resistance to CYP24A1-catalyzed catabolism does not appear to be a mechanism for AC-340 hyperagonism.

**Figure 6:**
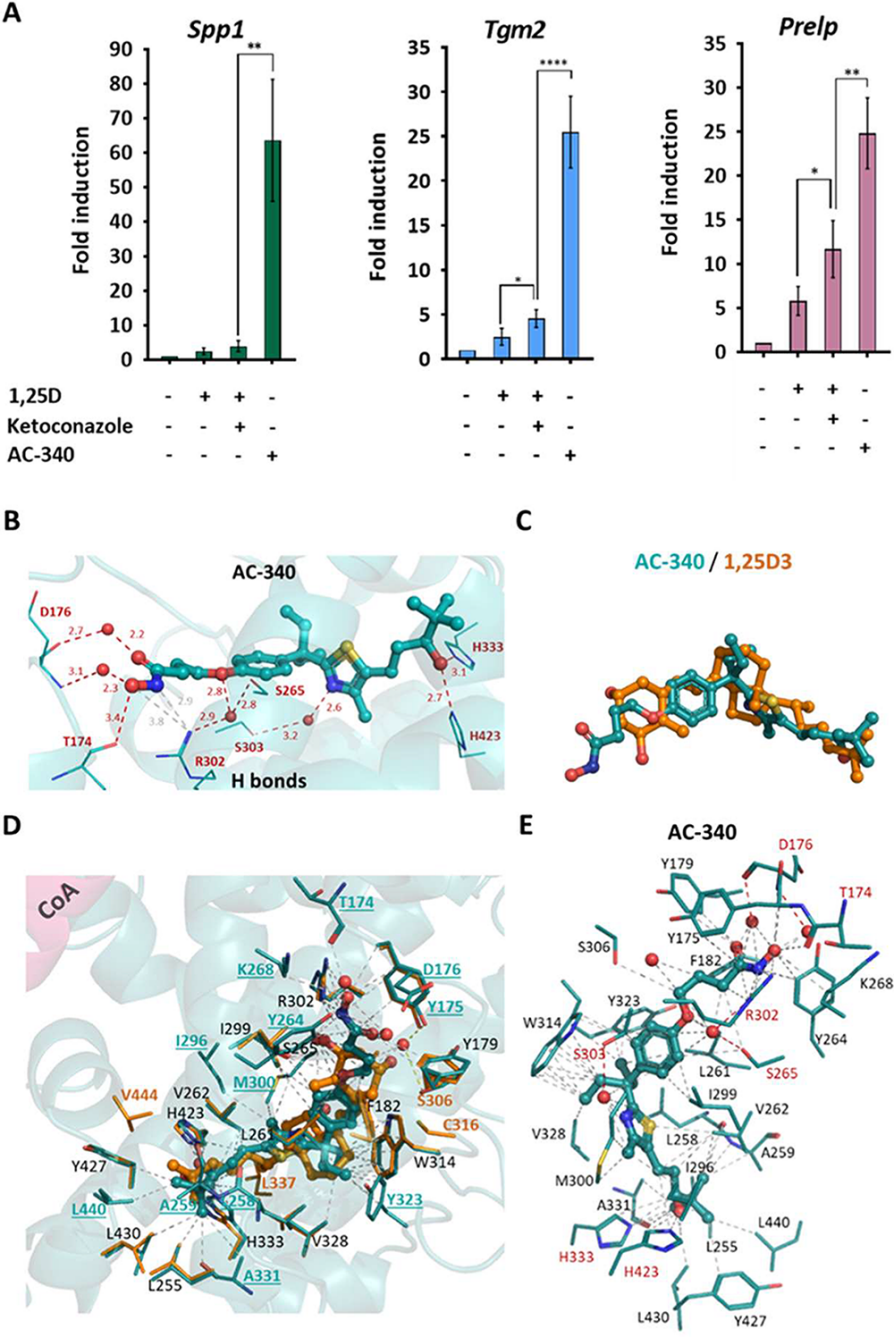
(A) Effect of CYP24A1 blockade by ketoconazole on the transcriptional activity of 1,25D in inducing VDR target genes. (B) Crystal structure of the zebrafish VDR bound to AC-340 (PDB 9RCG) showing the ligand within the binding pocket and the hydrogen bonds it forms with multiple residues. (C) Fitted structure of AC-340 onto 1,25D (PDB 2HC4) and (D) demonstrating how each ligand positions within the binding pocket. (E) Network of van der Waals interactions that AC-340 forms with multiple residues, notably with residues of H3, such as S265.

1,25D, like other nuclear receptor agonists, generates an active conformation of its receptor ligand binding domain by inducing a conformational change in the C-terminal helix 12 (H12). The induced movement of H12 creates a hydrophobic cleft, which provides a docking surface for coactivators recruited via amphipathic LxxLL α-helices (so-called nuclear receptor or NR boxes). To probe potential mechanisms of hyperagonism further, we determined the crystal structure of the zebrafish VDR bound to AC-340 and compared it to the 1,25D-bound structure (Figure 6B–E). Complexes were crystallized in the presence of an excess of ligand, and a peptide from the human nuclear receptor coactivator 2 (NCoA2) encompassing its second NR box. The AC-340 structure was solved by molecular replacement and refined to 2.1 Å. The electron density allowed unambiguous fitting of the ligands into the maps (Figure S6). When compared to the zVDR LBD-1,25D structure, the Cα atoms of the zVDR LBD-AC-340 complex had a root-mean-square deviation (rmsd) of 0.31 Å over 239 residues. As expected, AC-340 was buried in the hydrophobic pocket (Figure 6B), and the zVDR LBD structure adopted the canonical agonist conformation of all previously reported structures of the receptor bound to agonist, with helix 12 folded in the agonistic position.^47^ The chain with the AC-340 hydroxyl group formed hydrogen bonds with His333 and His423. The side chain containing the hydroxamic group occupied the region of the A-ring of 1,25D (Figure 6C) and formed H bonds with multiple residues in the binding pocket (Figure 6B). Notably, the nitrogen atom in the thiazole group formed a water-mediated hydrogen bond with Ser303 in H5 of the LBD. For comparison, the hydroxyl groups on the A-ring of 1,25D are anchored through hydrogen bonds with Arg302 and Ser265 for the C1-OH group and with Tyr175 and Ser306 for the C3-OH (Figure 6D).

AC-340 also formed a strong network of van der Waals interactions (Figure 6E; Table S1). It interacts strongly with Ser265 in H3, another component of the coactivator binding groove. Comparison of the two structures showed that AC-340 formed more numerous van der Waals interactions (102 interactions at a 4.0 Å cutoff) than 1,25D (71 interactions at a 4.0 Å cutoff) (Figure 6D). Notably, additional interactions were formed by AC-340 with the residues of the loop 1–2 (Thr174, Tyr175, Asp176), H3 (Leu258, Ala259, Tyr264, Lys268), H5 (Ile296, Met300), H6 (A331), β-sheet (Tyr323) and loop 11–12 (Leu440). In contrast, 1,25D formed stronger interactions with Ser306 (H5), Cys316 (β-sheet), Leu337 (H7) and Val444 (H12). The increased number of interactions of AC-340, notably with residues of H3, a key element of coactivator interactions, may explain the greater efficacy of AC-340 in inducing VDR target gene expression.

### Enhanced Coactivator Recruitment by AC-340

Structural studies described above suggest that AC-340 may act as a hyperagonist at least partly through increasing stabilization of the agonist-bound conformation and enhancing coactivator recruitment to chromatin by the DNA-bound VDR. To test this, we performed ChIP assays in B16–F10 cells, analyzing ligand-induced VDR and CBP (Creb binding protein) coactivator recruitment to the well-characterized consensus vitamin D receptor element (VDRE) in the mouse *Spp1* gene (Figure 7A), which is superinduced by AC-340 (Figures 4C and 6A). CBP was chosen because it is widely expressed, and previous studies have shown that it is recruited to *Cyp24a1* VDREs.^48^ Two h treatment of B16–F10 cells with either 1,25D or AC-340 induced similar levels of VDR recruitment to the *Spp1* VDRE (Figure 7B). In contrast, AC-340 recruited almost 3-fold more CBP under the same conditions (Figure 7C). CBP is a component of a large coactivator assembly recruited by VDR and required for chromatin remodeling due to its H3K27 acetyltransferase activity.^49–51^ Since we previously observed a substantially higher level of H3K27 acetylation in AC-340-treated B16–F10 cells (Figure 2C,D), we intend to assess H3K27 acetylation at the *Spp1* VDRE. Our ChIP-qPCR revealed that AC-340 treatment leads to a significant induction of H3K27 acetylation, whereas 1,25D was ineffective under the same conditions (Figure 7D). The same experiments were also performed on the tandem intronic VDREs in the mouse *Vdr* gene,^46^ where we observed similar levels of VDR recruitment but elevated binding of coactivator CBP and enhanced H3K27 acetylation in the presence of AC-340 relative to 1,25D (Figure S5).

**Figure 7:**
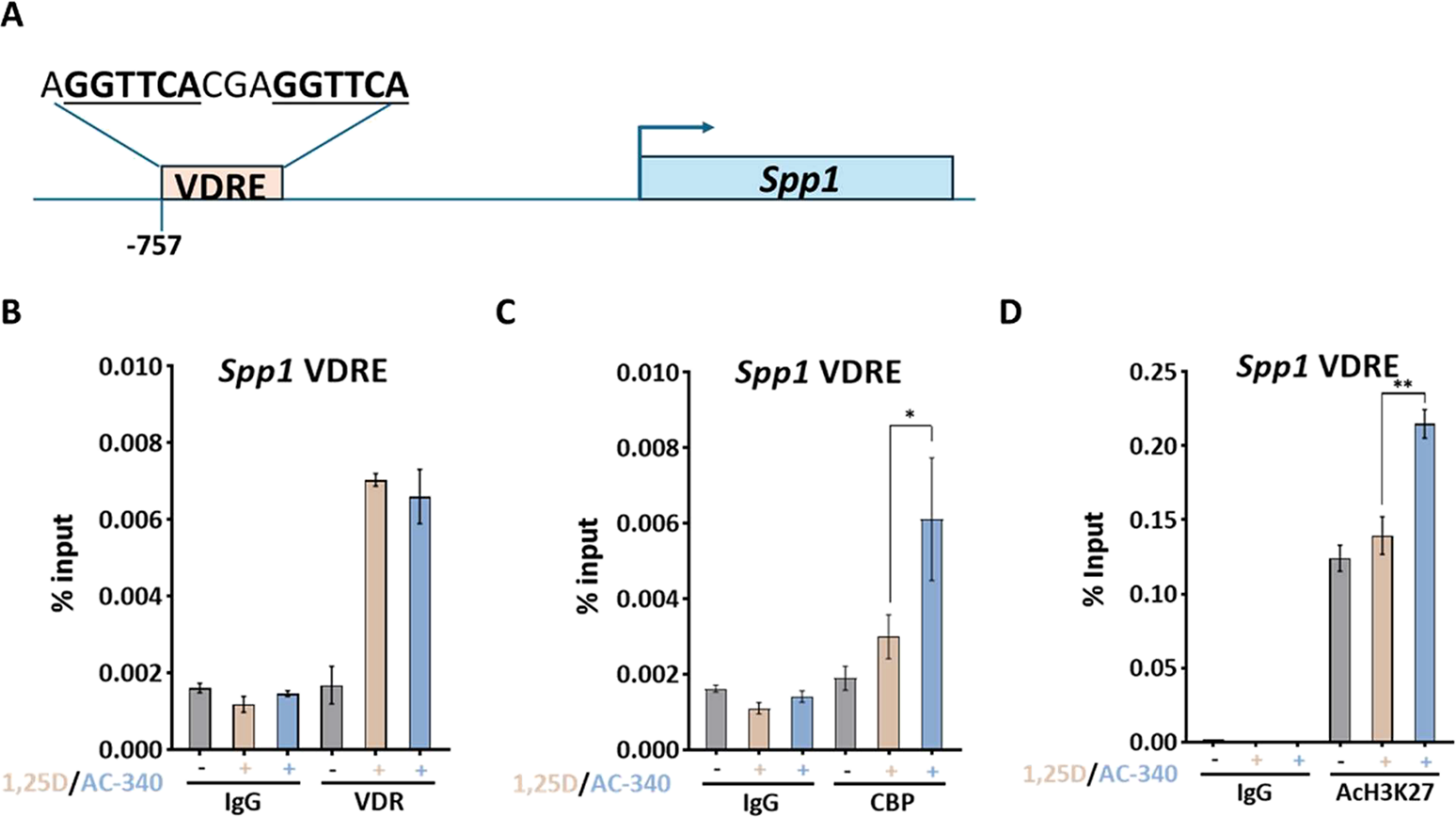
(A) VDRE sequence and location in *Spp1* gene. (B) ChIP-qPCR data showing the recruitment of VDR and (C) coactivator CBP to the gene promoter, along with (D) acetylation level of H3K27 in B16–F10 cell treated with 1,25D or AC-340 relative to control.

Here, we have explored the potential mechanisms of hyperagonism of VDR agonist/HDACi hybrid molecules observed in both human and mouse cell lines. Initial experiments in B16–F10 cells using *Cyp24a1* as a target gene suggested that hyperagonism was restricted to AC-340; *Cyp24a1* expression was induced several fold more by AC-340 than by 1,25D at saturating concentrations alone or with SAHA, AM-193 or ZG-126 (Figure 2A). ZG-126 was more efficacious than 1,25D (and AM-193) but not substantially different from 1,25D and SAHA combined in this assay. However, the initial observation on the *Cyp24a1* gene was not borne out by comparative gene expression profiling by RNA-seq, which revealed that all hybrids display some degree of hyperagonism, although AC-340 was generally the most efficacious, while AM-193 was the least. This suggested that the hyperagonism of AC-340 was not due simply to 1,25D-like VDR agonism combined with independent HDACi activity but rather that AC-340 was superactivating the VDR.

## Conclusions

While there are several possible mechanisms for hyperagonism, experiments presented above show that it does not correlate with (relative) changes in VDR expression observed in 1,25D- or hybrid-treated cells (Figure 5). While resistance to 1,25D-induced catabolism via Cyp24A1 may explain a portion of the differences in fold induction of gene expression seen in 1,25D- or hybrid-treated cells (Figure 6A), this does not appear to be the main driver of hyperagonism. Comparative crystal structure analyses of the 1,25D- and AC-340-bound zVDR ligand binding domains revealed, as expected, that the two LBDs adopted very similar agonist structures and that the hydroxamic acid-bearing side chain of AC-340 occupied the portion of the pocket bound by the secosteroidal A ring of 1,25D (Figure 6B–E). However, AC-340 differed from 1,25D by forming an increased number of contacts with key residues associated with the coactivator binding groove of the LBD, suggesting that AC-340 may stabilize the agonist-bound conformation and potentially enhance coactivator recruitment. This was borne out by ChIP assays, which revealed that 1,25D and AC-340 induced identical levels of VDR recruitment to chromatin on two genes after 2h, but that AC-340 treatment substantially enhanced CBP coactivator recruitment to the same sites (Figures 7 and S5). This correlated with increased levels of H3K27 acetylation induced by AC-340 at the same VDREs. Based on these findings, we hypothesize that enhanced coactivator recruitment by the AC-340-bound VDR contributes to its hyperagonism. What our studies do not quantify is the role of HDAC inhibition in the hyperagonist activity of AC-340. The HDACi activity of AC-340 should stabilize the increase in H3K27 acetylation (and induced acetylation of other histone marks) driven by its VDR agonist activity. However, it is unlikely that the HDACi activity of AC-340 accounts for all of the elevated levels of H3K27 acetylation or the effects on gene expression as the induction of vitamin D target genes by 1,25D and SAHA is generally lower than that observed with AC-340 (Figure 4C). While the HDACi activity of AC-340 functions independently of its VDR agonism (e.g., by inducing tubulin acetylation in the cytoplasm), the studies presented above highlight the potential complementarity of combining HDACi activity with VDR agonism in a hybrid molecule.

## Experimental Section

### Chemical Procedures

All compounds are >95% pure by HPLC analysis. Unless otherwise stated, reactions were conducted under an argon atmosphere and glassware was oven-dried prior to use. Tetrahydrofuran (THF) and diethyl ether were purified by distillation from sodium under a nitrogen atmosphere. Toluene, dichloromethane and triethylamine were purified by distillation from calcium hydride under nitrogen atmosphere. Deuterated chloroform was stored over activated 4 Å molecular sieves. All commercial reagents and solvents were used as purchased without further purification. Thin-layer chromatography (TLC) was carried out on glass-backed Ultrapure silica TLC plates (extra hard layer, 60 Å, thickness 250 μm, saturated with F-254 indicator) purchased from Silicycle. Flash column chromatography was carried out on 230–400 mesh silica gel (Silicycle) using reagent grade solvents. Proton and carbon nuclear magnetic resonance spectra were obtained on Varian 500 or Bruker 500 and 800 MHz spectrometers. Chemical shifts (δ) were internally referenced to the residual proton resonance including but not limited to CDCl_3_ (δ 7.26 ppm), CD_3_OD (δ3.31 ppm), and (CD_3_)_2_SO (δ 2.50 ppm). Coupling constants (*J*) are reported in Hertz (Hz). HRMS were obtained by Dr. Nadim Saadeh or Dr. Alexander S. Wahba at the McGill University Department of Chemistry. 1,25D (BML-DM200) and SAHA (#10009929) were purchased from Enzo Life Sciences and Cayman Chemical, respectively. They were both used at a final concentration of 100 nM.

#### 2-(4-(Benzyloxy)­phenyl)-2-ethylbutanenitrile (**9**)

To a solution of diisopropylamine (3.9 mL, 27.7 mmol, 2.8 equiv) in anhydrous THF (52 mL) at -78 °C was added *n*-butyllithium (10.3 mL, 2.5 M in hexanes) and the resulting solution was stirred for 1 min. A solution of **8** ^52^ (2.2 g, 9.89 mmol, 1 equiv) in 18 mL of anhydrous THF was added to the reaction mixture dropwise via cannula. The resulting mixture was stirred at −78 °C for 15 min. Ethyl iodide (2.2 mL, 27.7 mmol, 2.8 equiv) in 35 mL anhydrous THF was added dropwise to the reaction mixture via cannula. The resulting mixture was stirred for 15 min at −78 °C, then 1 h at 0 °C. The reaction was monitored by TLC. The reaction mixture was quenched by the addition of 1 M NH_4_Cl (aq.) (50 mL) and followed by extraction with ethyl acetate (50 mL). The organic layer was washed with brine, dried over anhydrous sodium sulfate and concentrated under reduced pressure. The residue was purified via chromatography (15:1 hexanes: ethyl acetate) to give **9** as a white solid (2.4 g, 88% yield). *R*_f_ = 0.35 (10:1 hexanes: ethyl acetate). IR (thin film) ν 2973, 2936, 2877, 2235 cm^–1^. ^1^H NMR (500 MHz, CDCl_3_) δ7.48–7.32 (m, 5H), 7.32–7.28 (m, 2H), 7.02–6.96 (m, 2H), 5.08 (s, 2H), 2.19–1.97 (m, 2H), 1.97–1.77 (m, 2H), 0.92 (t, *J* = 7.4 Hz, 6H). ^13^C NMR (126 MHz, CDCl_3_) δ 158.26, 136.93, 130.46, 128.77, 128.22, 127.69, 127.43, 122.67, 115.12, 70.25, 49.25, 34.00, 9.84. HRMS (ESI) calculated for C_19_H_21_NO [M + Na]^+^: 302.1521, found 302.1515.

#### 2,2,7-Trimethyloct-7-en-3-one (**13**)

To a solution of **12** ^53^ (8.7 g, 61 mmol, 1 equiv) in THF (100 mL) was added dropwise *n*-butyllithium (26.9 mL, 2.5 M solution in hexanes) at 0 °C. The mixture was stirred for 30 min, and 4-bromo-2-methylbut-1-ene^54^ (10 g, 67 mmol, 1.05 equiv) was added dropwise to the reaction mixture. The resulting solution was stirred at room temperature for 10 h, quenched by the addition of water (75 mL), and extracted with EtOAc (75 mL, 2x). The organic phase was concentrated, after which acetone (163 mL) and acidic resin (Amberlyte IR 120, 16.6 g) were added. The mixture was stirred for 12 h, filtered, concentrated, and purified by chromatography (5% ethyl acetate in hexanes) to give **13** as a yellow oil. (37% yield, 3.8 g). *R*_f_ = 0.45 (5:95, ethyl acetate to hexanes). IR (thin film) ν 2966, 2871, 1705, 1477, 1366 cm^–1^. ^1^H NMR (500 MHz, CDCl_3_) δ 4.73–4.71 (m, 1H), 4.68–4.67 (m, 1H), 2.47 (t, *J* = 7.3 Hz, 2H), 2.03–1.97 (m, 2H), 1.74–1.66 (m, 5H), 1.14 (s, 9H). ^13^C NMR (126 MHz, CDCl_3_) δ 216.02, 145.38, 110.51, 44.22, 37.26, 35.78, 26.56, 22.31, 21.70. HRMS (ESI) calculated for C_11_H_20_O­[M + Na]^+^; 191.1406, found 191.1403.

#### 2,2,7-Trimethyloct-7-en-3-ol (**14**)

To a solution of **13** (559 mg, 3.3 mmol, 1 equiv) in dry methanol (3.3 mL, 1.0 M) at 0 °C was slowly added NaBH_4_ (185 mg, 4.9 mmol, 1.5 equiv). The resulting mixture was warmed to room temperature and stirred for 1 h. Water (5 mL) was added, and the product was extracted with DCM (10 mL). The combined organic layers were washed with brine (10 mL), dried over anhydrous sodium sulfate and concentrated in vacuo. The residue was purified by chromatography (10% ethyl acetate in hexanes) to give **14** as a colorless oil (492 mg, 75% yield). *R*_f_ = 0.32 (10% Ethyl acetate in hexanes). IR (thin film) ν 3405, 2952, 2868, 1462, 1363 cm^–1^. ^1^H NMR (500 MHz, CDCl_3_) δ 4.70–4.69 (m, 1H), 4.68–4.67 (m, 1H), 3.19 (dd, *J* = 10.5, 2.8 Hz, 1H), 2.07–2.02 (m, 2H), 1.82–1.67 (m, 4H), 1.59–1.38 (m, 3H), 1.30–1.22 (m, 1H), 0.89 (s, 9H). ^13^C NMR (126 MHz, CDCl_3_) δ 146.05, 110.02, 80.01, 37.91, 35.10, 31.23, 25.82, 25.18, 22.50. HRMS (ESI) calculated for C_11_H_22_O­[M + Na]^+^; 193.1563, found 193.1568.

#### Tert-butyldimethyl­((2,2,7-trimethyloct-7-en-3-yl)­oxy)­silane (**15**)

To a solution of **14** (310 mg, 1.8 mmol, 1 equiv) in DMF (0.92 mL, 2 M) at 0 °C, was added imidazole (313 mg, 4.6 mmol, 2.55 equiv) followed by TBSCl (415 mg, 2.7 mmol, 1.5 equiv). The reaction mixture was stirred for 3 days at room temperature. Water (10 mL) was added, and the product was extracted with DCM (10 mL, 3x). The combined organic layers were washed with brine (15 mL) and dried over anhydrous sodium sulfate. The residue was purified via chromatography (2% ethyl acetate in hexanes) to give **15** as a colorless oil (322 mg, 63% yield). *R*_f_ = 0.72 (5% ethyl acetate in hexanes). IR (thin film) ν 2953, 2867, 1462, 1363 cm^–1^. ^1^H NMR (500 MHz, CDCl_3_) δ 4.72 (s, 1H), 4.69 (s, 1H), 3.25 (dd, *J* = 6.6, 3.2 Hz, 1H), 2.01–1.98 (m, 2H), 1.74 (s, 3H), 1.67–1.56 (m, 1H), 1.54–1.47 (m, 1H), 1.39–1.24 (m, 2H), 0.90 (s, 9H), 0.85 (s, 9H), 0.08 (3H), 0.06 (3H). ^13^C NMR (126 MHz, CDCl_3_) δ 146.18, 109.86, 80.82, 38.51, 36.02, 33.29, 26.65, 26.32, 25.85, 22.59, 18.57, −3.23, −3.78. HRMS (ESI) calculated for C_17_H_36_OSi [M + Na]^+^: 307.2428, found 307.2430.

#### 6-((*tert*-Butyldimethylsilyl)­oxy)-7,7-dimethyloctan-2-one (**16**)

A stream of ozone was bubbled through a solution of **15** (100 mg, 0.35 mmol, 1 equiv) in 6 mL dry dichloromethane at −78 °C until the solution turned blue. Excess ozone was removed by passing a stream of nitrogen through the solution. Triphenylphosphine (102 mg, 0.39 mmol, 1.05 equiv) was added slowly, and the solution was warmed to room temperature overnight. The solution was concentrated in vacuo and the residue purified by chromatography (2% diethyl ether in hexanes) to give **16** as a colorless oil (79 mg, 79% yield). *R*_f_ = 0.35 (5% ethyl acetate in hexanes). IR (thin film) ν 2954, 2856, 1718, 1360 cm^–1^. ^1^H NMR (500 MHz, CDCl_3_) δ 3.23 (dd, *J* = 6.9, 3.1 Hz, 1H), 2.41 (td, *J* = 7.2, 1.7 Hz, 2H), 2.15 (s, 3H), 1.80–1.68 (m, 1H), 1.57–1.48 (m, 2H), 1.35–1.21 (m, 1H), 0.92 (s, 9H), 0.86 (s, 9H), 0.07 (s, 3H), 0.03 (s, 3H). ^13^C NMR (126 MHz, CDCl_3_) δ 209.02, 80.56, 44.38, 35.99, 33.17, 29.94, 26.58, 26.28, 22.20, 18.52, −3.27, −3.84. HRMS (ESI) calculated for C_16_H_34_O_2_Si [M + Na]^+^: 309.2226, found 309.2224.

#### 2-(4-(Benzyloxy)­phenyl)-2-ethylbutanamide (**10**)

In a screw capped vial was added freshly powdered potassium hydroxide (430 mg, 7.65 mmol, 2 equiv), 8 mL of dry t-butanol (dried over 3Å molecular sieves), and **9** (1.07g, 3.8 mmol, 1 equiv). The vial was sealed, and the solution was stirred at 100 °C for 5 days. The reaction mixture was diluted with dichloromethane (8 mL) and water (10 mL). The aqueous layer was extracted with DCM (8 mL, 3x). The combined organic layers were dried over anhydrous sodium sulfate, concentrated under reduced pressure and purified via chromatography (1:1 hexanes to ethyl acetate) to give **10** as a white solid (0.83 g, 73% yield). *R*_f_ = 0.36 (1:1 hexanes to ethyl acetate). IR (thin film) ν 3468, 3156, 2968, 2934, 2877, 1667, 1509, 1455 cm^–1^. ^1^H NMR (500 MHz, CDCl_3_) δ 7.50–7.31 (m, 5H), 7.27–7.22 (m, 2H), 7.03–6.89 (m, 2H), 5.19 (d, *J* = 19.5 Hz, 2H), 5.07 (s, 2H), 2.06–1.89 (m, 4H), 0.77 (t, *J* = 7.4 Hz, 6H). ^13^C NMR (126 MHz, CDCl_3_) δ 179.07, 157.76, 137.08, 135.64, 128.76, 128.47, 128.17, 127.66, 114.85, 70.18, 54.05, 27.21, 8.45. HRMS (ESI) calculated for C_19_H_23_NO_2_ [M + Na]^+^: 320.1626, found 320.1621.

#### 2-(4-(Benzyloxy)­phenyl)-2-ethylbutanethioamide (**11**)

To a solution of **10** (500 mg, 1.7 mmol, 1 equiv) in anhydrous THF (4 mL, 0.4 M), Lawesson’s reagent (414 mg, 1 mmol, 1.1 equiv) was added at room temperature. The reaction mixture was heated to 45 °C for 3 h and cooled to room temperature. Ethyl acetate (10 mL) and saturated sodium bicarbonate (10 mL) were added to the reaction mixture. The aqueous layer was extracted with ethyl acetate (10 mL x 3), the combined organic layers were dried over anhydrous sodium sulfate and concentrated under reduced pressure. The residue was purified via chromatography (gradient of 15 to 50% ethyl acetate in hexanes) to give **11** as a white solid (113 mg, 26% yield). *R*_f_ = 0.37 (30% ethyl acetate in hexanes). IR (thin film) ν 3395, 3270, 3153, 2971, 2935, 2875, 1619, 1506, 1454 cm^–1^. ^1^H NMR (500 MHz, CDCl_3_) δ 7.56 (s, 1H), 7.50–7.35 (m, 5H), 7.28–7.25 (m, 2H), 7.06–6.91 (m, 2H), 6.54 (s, 1H), 5.08 (s, 2H), 2.22 (dq, *J* = 14.5, 7.3 Hz, 2H), 2.12–2.02 (m, 2H), 0.78 (t, *J* = 7.4 Hz, 6H). ^13^C NMR (126 MHz, CDCl_3_) δ 216.71, 157.89, 136.98, 135.57, 128.76, 128.73, 128.20, 127.65, 114.98,, 70.17, 58.16, 30.54, 8.82. HRMS (ESI) calculated for C_19_H_23_NOS [M + Na]^+^: 336.1398, found 336.1393.

#### 3-Bromo-6-((*tert*-butyldimethylsilyl)­oxy)-7,7-dimethyloctan-2-one (**17a**)

To a mixture of **16** (690 mg, 2.4 mmol, 1 equiv) and NBS (448 mg, 2.52 mmol, 1.05 equiv) in CCl_4_ (3 mL) was added NH_4_OAc (19 mg, 0.024, 0.1 equiv) at room temperature. After stirring at 80 °C for 15 min, the mixture was cooled to room temperature and diluted with DCM (10 mL) and water (10 mL). The aqueous phase was extracted with DCM (10 mL, 3x). The combined organic layers were dried over anhydrous sodium sulfate and concentrated under reduced pressure. The residue was purified via chromatography (1% ether in hexanes) to give **17a** and **17b** as a yellow oil (4:1 rr, 559 mg, 64% yield). *R*_f_ = 0.35 (2% diethyl ether in hexanes). The mixture of α-bromoketones (100 mg, 0.27 mmol, 1 equiv) was added to a solution of 4-chlorothiophenol (8 mg, 0.067 mmol, 0.25 equiv) and potassium carbonate (10 mg, 0.067 mmol, 0.25 equiv) in ethanol (0.6 mL) at room temperature. The reaction mixture was stirred for 2 h, then filtered through a thin pad of Celite and washed with ethanol (5 mL). The solution was concentrated under reduced pressure and purified by chromatography (1% diethyl ether in hexanes) to give **17a** as a yellow oil (20:3 rr, 1:1 *dr*, 68 mg, 68% yield). IR (thin film) ν 2954, 2856, 1720, 1472, 1359 cm^–1^. ^1^H NMR (500 MHz, CDCl_3_) δ 4.24–4.15 (m, 1H) 3.32–3.23 (m, 1H), 2.38 (d, *J* = 3.5 Hz, 3H), 2.29–1.71 (m, 4H), 0.98–0.92 (m, 9H), 0.92–0.82 (m, 9H), 0.13–0.02 (m, 6H). ^13^C NMR (126 MHz, CDCl_3_) δ 202.06, 202.04, 79.99, 79.96, 55.20, 55.00, 36.15, 36.09, 31.92, 31.62, 31.27, 31.21, 26.62, 26.61, 26.34, 26.29, 26.27, 26.05, 18.53, 18.52, −3.14, −3.15, −3.74, −3.76. HRMS (ESI) calculated for C_16_H_33_O_2_BrSi [M + Na]^+^: 387.1331, found 387.1324.

#### 1-(2-(3-(4-(Benzyloxy)­phenyl)­pentan-3-yl)-5-methylthiazol-4-yl)-4,4-dimethylpentan-3-ol (**18**)

To a solution of **11** (256 mg, 0.82 mmol, 1 equiv) in anhydrous ethanol (1.6 mL, 0.5 M) was added **17a** (329 mg, 0.9 mmol, 1.1 equiv). The reaction was heated at reflux overnight. The solvent was removed under reduced pressure and the crude mixture was dissolved in ethyl acetate (2 mL) and water (2 mL). The aqueous layer was extracted with ethyl acetate (2 mL, 3x). The combined organic layers were washed with brine (6 mL) and dried over anhydrous sodium sulfate. The residue was purified by chromatography (10% ethyl acetate in hexanes) to give **18** as a white solid (257 mg, 67% yield). *R*_f_ = 0.14 (20% ethyl acetate in hexanes). IR (thin film) ν 3364, 2961, 2924, 2864, 1607, 1509 cm^–1^. ^1^H NMR (500 MHz, CDCl_3_) δ 7.45–7.40 (m, 2H), 7.40–7.35 (m, 2H), 7.34–7.29 (m, 1H), 7.23–7.18 (m, 2H), 6.93–6.86 (m, 2H), 5.05 (s, 2H), 3.18 (dd, *J* = 10.6, 1.8 Hz, 1H), 2.95 (ddd, *J* = 14.5, 9.4, 4.7 Hz, 1H), 2.73 (ddd, *J* = 15.0, 9.1, 7.3 Hz, 1H), 2.35 (s, 3H), 2.31–2.17 (m, 4H), 1.78–1.72 (m, 1H), 1.58–1.50 (m, 1H), 1.44 (br. s, 1H), 0.87 (s, 9H), 0.76–0.69 (m, 6H). ^13^C NMR (126 MHz, CDCl_3_) δ 175.30, 157.14, 146.20, 138.77, 137.15, 131.26, 128.57, 128.41, 127.94, 127.56, 114.13, 79.02, 69.97, 51.00, 34.92, 33.36, 29.71, 29.69, 25.62, 23.88, 15.10, 8.30, 8.29. HRMS (ESI) calculated for C_29_H_39_NO_2_S [M + H]^+^: 466.2780, found 466.2768.

#### 4-(3-(4-(3-Hydroxy-4,4-dimethylpentyl)-5-methylthiazol-2-yl)­pentan-3-yl)­phenol (**19**)

Pd/C (12 mg, 10 wt %) was added in one portion to **18** (264 mg, 0.57 mmol, 1 equiv) in anhydrous methanol (6.4 mL, 0.09 M) at room temperature. The reaction vessel was filled with hydrogen gas. The reaction mixture was stirred at room temperature overnight, then filtered over a thin pad of Celite and washed with methanol (10 mL). The filtrate was concentrated under reduced pressure and purified via chromatography (30% ethyl acetate in hexanes) to give **19** as a pale-yellow solid (169 mg, 79% yield). *R*_f_ = 0.25 (30% ethyl acetate in hexanes) IR (thin film) ν 3367, 2961, 2925, 2864, 1509, 1455 cm^–1^. ^1^H NMR (500 MHz, CDCl_3_) δ 7.71 (br. s, 1H), 6.96 (t, *J* = 7.7 Hz, 2H), 6.44 (d, *J* = 9.9 Hz, 2H), 3.22 (dd, *J* = 10.5, 5.3 Hz, 1H), 2.99 (ddd, *J* = 14.5, 9.4, 4.7 Hz, 1H), 2.77 (dt, *J* = 15.5, 8.2 Hz, 1H), 2.33 (s, 3H), 2.33–2.27­(m, 2H), 2.18–2.11 (m, 2H), 1.84–1.77 (m, 1H), 1.69–1.59 (m, 1H), 1.42 (d, *J* = 7.4, Hz, 1H), 0.92 (S, 9H), 0.71 (t, *J* = 7.3 Hz, 6H). ^13^C NMR (126 MHz, CDCl_3_) δ 177.45, 155.25, 147.05, 136.19, 130.51, 128.02, 115.18, 79.18, 50.64, 35.09, 33.47, 28.79, 28.88, 25.77, 23.88, 14.53, 8.23. HRMS (ESI) calculated for C_22_H_33_NO_2_S [M + H]^+^: 376.2305, found 376. 2303.

#### Ethyl 4-(4-(3-(4-(3-Hydroxy-4,4-dimethylpentyl)-5-methylthiazol-2-yl)­pentan-3- yl)­phenoxy)­butanoate (**20a**)

Ethyl 4-bromobutyrate (16 μL, 0.1 mmol, 1.25 equiv) was added in one portion to a vigorously stirred slurry of **19** (31 mg, 0.08 mmol, 1 equiv) and solid K_2_CO_3_ (22 mg, 0.16 mmol, 2 equiv) in MeCN (0.4 mL) under air. The reaction mixture was heated at reflux overnight, at which point TLC analysis indicated complete consumption of the starting material. The reaction mixture was allowed to cool to room temperature and filtered over a pad of Celite via vacuum filtration. The filtrate was collected as a clear solution and concentrated to a clear oil. The residue was purified by chromatography (30% ethyl acetate in hexanes) to provide **20a** as a clear oil (39 mg, 98% yield). *R*_f_ = 0.40 (30% ethyl acetate in hexanes). IR (thin film) ν 3516, 2963, 2872, 1733, 1609, 1510 cm^–1^. ^1^H NMR (500 MHz, CDCl_3_) δ 7.26–7.15 (m, 2H), 6.85–6.77 (m, 2H), 4.15 (q, *J* = 7.1 Hz, 2H), 3.99 (t, *J* = 6.1 Hz, 2H), 3.17 (dd, *J* = 10.6, 1.8 Hz, 1H), 2.94 (ddd, *J* = 14.4, 9.4, 4.7 Hz, 1H), 2.72 (ddd, *J* = 15.0, 9.1, 7.4 Hz, 1H), 2.51 (t, *J* = 7.3 Hz, 2H), 2.34 (s, 3H), 2.30–2.17 (m, 4H), 2.17–2.09 (m, 2H), 1.77–1.70 (m, 1H), 1.60–1.50 (m, 1H), 1.28–1.24 (m, 4H), 0.88 (s, 9H), 0.73 (td, *J* = 7.3, 1.0 Hz, 6H). ^13^C NMR (126 MHz, CDCl_3_) δ 175.44, 173.38, 157.20, 146.25, 138.63, 131.37, 128.47, 113.88, 79.10, 66.66, 60.50, 51.07, 35.02, 33.47, 30.95, 29.79, 29.77, 25.72, 24.81, 23.98, 15.19, 14.31, 8.39, 8.37. HRMS (ESI) calculated for C_28_H_43_NO_4_S [M + H]^+^: 490.2985, found 490.2981.

#### Ethyl 5-(4-(3-(4-(3-Hydroxy-4,4-dimethylpentyl)-5-methylthiazol-2-yl)­pentan-3- yl)­phenoxy)­pentanoate (**20b**)

Ethyl 5-bromovalerate (16 μL, 0.1 mmol, 1.25 equiv) was added in one portion to a vigorously stirred slurry of **19** (29.2 mg, 0.07 mmol, 1 equiv) and solid K_2_CO_3_ (22 mg, 0.16 mmol, 2 equiv) in MeCN (0.4 mL) under air. The reaction mixture was heated at reflux overnight, at which point TLC analysis indicated complete consumption of the starting material. The reaction mixture was allowed to cool to room temperature and filtered over a pad of Celite via vacuum filtration. The filtrate was collected as a clear solution and concentrated to a clear oil. The residue was purified by chromatography (30% ethyl acetate in hexanes) to provide **20b** as a clear oil (37 mg, 97% yield,). *R*_f_ = 0.35 (30% ethyl acetate in hexanes). IR (thin film) ν 3517, 2963, 2872, 1732, 1609, 1510, 1468 cm ^–1^. ^1^H NMR (500 MHz, CDCl_3_) δ 7.23–7.14 (m, 2H), 6.85–6.75 (m, 2H), 4.14 (q, *J* = 7.2 Hz, 2H), 4.02–3.93 (m, 2H), 3.17 (dd, *J* = 10.6, 1.8 Hz, 1H), 2.94 (ddd, *J* = 14.5, 9.4, 4.7 Hz, 1H), 2.72 (ddd, *J* = 14.9, 9.1, 7.4 Hz, 1H), 2.43–2.37 (m, 2H), 2.34 (s, 3H), 2.28–2.16 (m, 4H), 1.87–1.78 (m, 4H), 1.76–1.70 (m, 1H), 1.57–1.49 (m, 1H), 1.27 (m, 4H), 0.88 (s, 9H), 0.73 (t, *J* = 7.3, 6H). ^13^C NMR (126 MHz, CDCl_3_) δ 175.52, 173.61, 157.33, 146.25, 138.51, 131.37, 128.47, 113.87, 79.12, 67.31, 60.42, 51.08, 35.02, 34.10, 33.47, 29.80, 29.78, 28.85, 25.73, 23.99, 21.82, 15.20, 14.37, 8.40, 8.38. HRMS (ESI) calculated for C_29_H_45_NO_4_S [M + H]^+^: 504.3142, found 504.3134.

#### Ethyl 6-(4-(3-(4-(3-Hydroxy-4,4-dimethylpentyl)-5-methylthiazol-2-yl)­pentan-3- yl)­phenoxy)­hexanoate (**20c**)

Ethyl 6-bromohexanoate (19 μL, 0.1 mmol, 1.25 equiv) was added in one portion to a vigorously stirred slurry of **19** (28 mg, 0.07 mmol, 1 equiv) and solid K_2_CO_3_ (22 mg, 0.16 mmol, 2 equiv) in MeCN (0.4 mL) under air. The reaction mixture was heated at reflux overnight, at which point TLC analysis indicated complete consumption of the starting material. The reaction mixture was allowed to cool to room temperature and filtered over a pad of Celite via vacuum filtration. The filtrate was collected as a clear solution and concentrated to a clear oil. The residue was purified by chromatography (30% ethyl acetate in hexanes) to provide **20c** as a clear oil (38 mg, 99% yield). *R*_f_ = 0.42 (30% ethyl acetate in hexanes). IR (thin film) ν 3488, 2939, 2869, 1731, 1609, 1510, 1467 cm^–1^. ^1^H NMR (500 MHz, CDCl_3_) δ 7.23–7.15 (m, 2H), 6.85–6.77 (m, 2H), 4.23–4.01 (m, 2H), 3.94 (t, *J* = 6.4 Hz, 2H), 3.17 (dd, *J* = 10.6, 1.8 Hz, 1H), 2.93 (ddd, *J* = 14.5, 9.4, 4.7 Hz, 1H), 2.72 (ddd, *J* = 14.9, 9.1, 7.4 Hz, 1H), 2.33 (d, *J* = 7.4 Hz, 5H), 2.28–2.11 (m, 4H), 1.93–1.62 (m, 5H), 1.59–1.44 (m, 3H), 1.28–1.24 (m, 4H), 0.87 (s, 9H), 0.73 (t, *J* = 7.9 Hz, 6H). ^13^C NMR (126 MHz, CDCl_3_) δ 175.50, 173.76, 157.40, 146.22, 138.43, 131.36, 128.44, 113.86, 79.09, 67.57, 60.34, 51.06, 35.01, 34.38, 33.46, 29.79, 29.77, 29.13, 25.81, 25.72, 24.84, 23.98, 15.19, 14.36, 8.39, 8.38. HRMS (ESI) calculated for C_30_H_47_NO_4_S [M]^+^: 518.3298, found 518.3297.

#### *N*-Hydroxy-4-(4-(3-(4-(3-hydroxy-4,4-dimethylpentyl)-5-methylthiazol-2-yl)­pentan-3- yl)­phenoxy)­butanamide (**7a**; AC-340)

Hydroxylamine (1.67 mL, 25 mmol, aq. 50 wt % solution, 500 equiv), and 3 M KOH (aq.) (0.12 mL, 0.35 mmol, 7 equiv) were added in that order to a vigorously stirring solution of **20a** (32.8 mg, 0.067 mmol, 1 equiv) at 0 °C in a 1:1 MeOH/THF mixture (6.5 mL) under air. The reaction was left to warm slowly to room temperature overnight. After 24 h, volatiles were removed in vacuo and the residue was dissolved in HPLC grade ethyl acetate. The organic layer was washed with a sodium phosphate buffer (pH = 7) (5 mL). The organic layer was concentrated in vacuo and the residue was purified by reversed-phase chromatography on octadecyl-functionalized silica gel using a gradient of 60–95% MeOH:H2O over a period of 20 min as eluent to provide **7a** (AC-340) as a fine white powder (13.7 mg) in 43% yield after lyophilization. IR (thin film) ν 3213, 2963, 2873, 1652, 1510, 1469 cm ^–1^. ^1^H NMR (500 MHz, DMSO-*d*_6_) δ 10.39 (s, 1H), 8.68 (s, 1H), 7.17–7.11 (m, 2H), 6.86–6.79 (m, 2H), 4.42 (d, *J* = 6.4 Hz, 1H), 3.91 (t, *J* = 6.3 Hz, 2H), 2.93 (ddd, *J* = 10.5, 6.4, 1.7 Hz, 1H), 2.84 (ddd, *J* = 14.4, 9.5, 4.4 Hz, 1H), 2.62 (ddd, *J* = 15.0, 9.2, 7.3 Hz, 1H), 2.21 (s, 3H), 2.12 (p, *J* = 7.4 Hz, 6H), 1.90 (p, *J* = 6.7 Hz, 2H), 1.65–1.55 (m, 1H), 1.42–1.28 (m, 1H), 0.77 (s, 9H), 0.64 (td, *J* = 7.3, 1.5 Hz, 6H). ^13^C NMR (126 MHz, DMSO- *d*_6_) δ 173.95, 168.60, 156.72, 145.56, 137.78, 131.56, 128.02, 113.83, 76.71, 66.64, 50.37, 34.67, 33.35, 28.99, 28.74, 25.86, 24.86, 23.31, 14.80, 8.19. HRMS (ESI-) calculated for C_26_H_40_N_2_O_4_S [M – H]^−^: 475.2636, found 475.2637. Purity >99% by LC–MS, tr = 11.5 min.

#### *N*-Hydroxy-5-(4-(3-(4-(3-hydroxy-4,4-dimethylpentyl)-5-methylthiazol-2-yl)­pentan-3- yl)­phenoxy)­pentanamide (**7b**; AC-339)

Hydroxylamine (1.67 mL, 25 mmol, aq. 50 wt % solution, 500 equiv), and 3 M KOH (aq.) (0.12 mL, 0.35 mmol, 7 equiv) were added in that order to a vigorously stirring solution of **20b** (30 mg, 0.059 mmol, 1 equiv) at 0 °C in a 1:1 MeOH/THF mixture (6.5 mL) under air. The reaction was left to warm slowly to room temperature overnight. After 24 h, volatiles were removed in vacuo and the residue was purified by reversed-phase chromatography on octadecyl-functionalized silica gel using a gradient of 50–95% MeOH:H2O over a period of 20 min as eluent to provide **7b** (AC-339) as a fine white powder (27 mg) in 92% yield after lyophilization. IR (thin film) ν 3231, 2939, 2873, 1643, 1510, 1393 cm^–1^. ^1^H NMR (500 MHz, DMSO) δ 7.19–7.09 (m, 2H), 7.02–6.70 (m, 2H), 4.44 (s, 1H), 3.92 (t, *J* = 6.2 Hz, 2H), 2.95 (d, *J* = 10.4 Hz, 1H), 2.85 (ddd, *J* = 14.4, 9.4, 4.5 Hz, 1H), 2.71–2.57 (m, 1H), 2.23 (s, 3H), 2.14 (q, *J* = 7.3 Hz, 4H), 1.97 (br. s, 2H), 1.73–1.54 (m, 5H), 1.44–1.31 (m, 1H), 0.78 (s, 9H), 0.65 (, *J* = 7.3 Hz, 6H). ^13^C NMR (126 MHz, DMSO) δ 173.98, 168.88, 156.83, 145.55, 137.64, 131.56, 127.99, 113.80, 76.70, 66.93, 50.36, 34.67, 33.34, 32.07, 28.99, 28.27, 25.86, 23.31, 21.92, 14.80, 8.19. HRMS (ESI+) calculated for C_27_H_42_N_2_O_4_S [M + H]^+^: 491.2938, found 491.2938. Purity >96% by LC–MS, tr = 11.84 min.

#### *N*-Hydroxy-6-(4-(3-(4-(3-hydroxy-4,4-dimethylpentyl)-5-methylthiazol-2-yl)­pentan-3- yl)­phenoxy)­hexanamide (**7c**; AC-341)

Hydroxylamine (1.67 mL, 25 mmol, aq. 50 wt % solution, 500 equiv), and 3 M KOH (aq.) (0.12 mL, 0.35 mmol, 7 equiv) were added in that order to a vigorously stirring solution of **20c** (29.8 mg, 0.057 mmol, 1 equiv) at 0 °C in a 1:1 MeOH/THF mixture (6.5 mL) under air. The reaction was left to warm slowly to room temperature overnight. After 24 h, volatiles were removed in vacuo and the residue was purified by reversed-phase chromatography on octadecyl-functionalized silica gel using a gradient of 60–95% MeOH:H2O over a period of 20 min as eluent to provide **7c** (AC-341) (23.1 mg, 80% yield) as a fine white powder after lyophilization. IR (thin film) ν 3213, 2937, 2865, 1633, 1509 cm^–1^. ^1^H NMR (500 MHz, DMSO) δ 7.19–7.09 (m, 2H), 6.86–6.80 (m, 2H), 4.44 (br. s, 1H), 3.92 (t, *J* = 6.2 Hz, 2H), 2.95 (d, *J* = 10.4 Hz, 1H), 2.85 (ddd, *J* = 14.4, 9.7, 4.5 Hz, 1H),), 2.71–2.57 (m, 1H), 2.23 (s, 3H), 2.14 (q, *J* = 7.3 Hz, 4H), 1.90 (s, 2H), 1.66 (p, J = 6.7 Hz, 2H), 1.62–1.55 (m, 1H), 1.53–1.48 (m, 2H), 1.39–1.31 (m, 3H), 0.78 (s, 9H), 0.65 (t, *J* = 7.3 Hz, 6H). ^13^C NMR (126 MHz, DMSO) δ 173.98, 168.99, 156.84, 145.55, 137.64, 131.56, 127.99, 113.78, 76.70, 67.17, 50.35, 34.67, 33.34, 32.46, 28.99, 28.52, 25.86, 25.27, 24.87, 23.31, 14.80, 8.19. HRMS (ESI-) calculated for C_28_H_44_N_2_O_4_S [M – H]^−^: 503.2949, found 503.2948. Purity >98% by LC–MS, tr = 12.40 min.

### Biological Evaluation

#### Cell Culture

Mouse B16–F10 and human A375 cells were cultured in Dulbecco’s Modified Eagle Medium (DMEM; 319-005-CL; WISENT Inc.) supplemented with 10% heat-inactivated Fetal Bovine Serum (FBS; 098150; WISENT Inc.), while human SK-MEL-28 melanoma cells were cultured in Eagle’s Minimum Essential Medium (EMEM; 30-2003; ATCC) supplemented with 10% heat-inactivated FBS for further experiments.

#### RNA Extraction, Reverse Transcription, and qPCR

B16–F10, A375 and SK-MEL-28 cells were treated with DMSO (vehicle), 1,25D, SAHA, combination of 1,25D and SAHA, and hybrid molecule(s) for 24 h. RNA extraction was performed with the Blood/Cultured cell total RNA purification mini kit (FAVORGEN Biotech Corp. #FABRK 001-2) as per manufacturer’s instructions. cDNA was obtained from 1 μg of RNA using 5X All-in-One RT Mastermix (Applied Biological Materials (abm) Inc. #G485) and diluted 5 times. qPCR was performed with BrightGreen 2X q-PCR MasterMix (abm MasterMix-LR-XL) on a Roche LightCycler 96 system. Expression of target genes were normalized to 18s. All primer sequences are listed in Table S3.

#### Biochemical Assays for HDAC Inhibition

Purified HDAC2 and HDAC6 were purchased from Cayman Chemical. Boc-Lys­(Ac)-7-amino-4-methylcoumarin (BocLys­(Ac)-AMC) was used as substrate for the HDAC assays. Substrate solution was prepared as follows: Boc­(Lys-Ac)-AMC was dissolved in DMSO and diluted with HDAC buffer (15 mM tris–HCl [pH 8.1], 250 μM EDTA, 250 mM NaCl, 10% glycerol) to give 1 mM solutions containing 1.7% DMSO. In parallel, each inhibitor diluted in 50 μL of HDAC buffer was mixed with 10 μL of diluted enzyme solution in HDAC buffer at room temperature (RT). This was followed by adding 40 μL of substrate solution into the mix and 1 h incubation with stirring at 37 °C. The reaction was stopped by adding 100 μL of trypsin solution and the release of AMC was monitored by measuring the fluorescence at 460 nm (λ_ex_ = 390 nm) with a microplate reader (SpectraMax Gemini from Molecular Devices) at 37 °C. The AMC signals were recorded against a blank with buffer, substrate and trypsin but without the enzyme. All experiments were carried out at least in triplicate.

Assays for 11 HDACs inhibition were performed by Reaction Biology (Malvern, PA, USA) using standard in-house protocols. Briefly, AC-340 was tested in 10-dose IC_50_ mode, in singlicate, with 3-fold serial dilution starting at 100 μM. The HDACi reference compounds TSA, TMP269 and Quisinostat were tested in a 10-dose IC_50_, with 3-fold serial dilution starting at 10 μM or 1 μM, depending on the HDAC tested. For HDACs 1, 2, 3 and 6, a fluorogenic peptide from p53 residues 379–382 (RHKK­(Ac)­AMC) was used as a substrate, while a fluorogenic peptide from p53 residues 379–382 (RHK­(Ac)­K­(Ac)­AMC) was used as a substrate for HDAC8. Trifluoroacetyl Lysine was used as the substrate for HDACs 4, 5, 7, 9 and 11, whereas Ac-Spermidine-AMC was the substrate for HDAC10 assays.

#### Western Blotting and Protein Analysis

After 6 h treatments with DMSO, 1,25D, SAHA, combination of 1,25D and SAHA, and hybrid molecule(s), B16–F10, A375 and SK-MEL-28 cells were solubilized in lysis buffer (20 mM Tris, pH 8, 150 mM NaCl, 1% Triton X-100, 3.5 mM SDS, 13 mM deoxycholic acid) supplemented with 1% protease inhibitor cocktail. Lysates were then sonicated on a Bioruptor with a 3 repeated cycle of 10 s ON/10 s OFF. To remove cellular debris, the samples were centrifuged for 5 min at 10,000 rpm at 4 °C followed by heating at 65 °C for 15 min for protein denaturation prior to running on a 4–15% Tris/Glycine/SDS gel (Bio-Rad- #4568084). Subsequently, the proteins were transferred to a 0.45 μm nitrocellulose membrane (Cytiva- #10600002) followed by blocking in 5% skim milk in Tris-buffered saline solution (TBS, 1X). After blocking, the membranes were incubated overnight at 4 °C with the primary antibodies listed in Table S4. Then the membranes were incubated with secondary antibodies (Table S4) at recommended concentrations for 1 h at RT. Finally, signals were detected using Clarity Max Western ECL Substrates (Bio-Rad- #1705062) and ChemiDoc Imaging System. Changes in protein levels were quantified relative to control (DMSO) using Image Lab software (Version 6.0.1) after normalization to GAPDH.

#### RNA Sequencing

B16–F10 cells were prepared in triplicate and RNA was extracted after 24 h treatment with DMSO, 1,25D, SAHA, 1,25D + SAHA and hybrid molecules. RNA concentrations were measured to ensure that each sample had a minimum concentration of 100 ng/μL and a 260/280 ratio around 2 prior to submission to the McGill Genome Centre. A quality control was performed before sequencing in paired-end at 50 M reads by Illumina NovaSeq 6000 S4 PE100. After sequencing, the FASTQ files were sent to us and all downstream analysis were performed on Galaxy (Galaxy (usegalaxy.org)). First, trimmomatic tool was used to remove the adapters from the reads. Then, using the alignment tool, HISAT2, the reads were mapped to the mouse genome. In the next step, the counted reads were annotated and finally the list of differentially expressed genes (DEGs) for each treatment was obtained using the preinstalled R package called Limma.

#### Biochemistry, Crystallization, and Structure Determination

The cDNA encoding His-tagged zVDR LBD (156–453) was cloned into pET28b. The recombinant proteins were produced in *Escherichia coli* BL21 DE3 grown ON at 18 °C after induction with 1 mM IPTG at an OD600 of ∼0.7. Soluble proteins were purified on Ni Hitrap FF crude column (Cytiva), followed by His tag removal by thrombin cleavage and by size exclusion chromatography on HiLoad Superdex 75 column (Cytiva) equilibrated in Tris 20 mM pH 7, NaCl 200 mM, TCEP 1 mM. The proteins were concentrated to 3–7 mg/mL with an Amicon Ultra 30 kDa MWCO and incubated with a 2-fold excess of ligand and a 3-fold excess of the coactivator NCoA2 NR2 (KHKILHRLLQDSS) peptide. Crystals of VDR - AC-340 were obtained at 293 K in Na_2_ Tartrate 1.1 M, MgCl_2_ 0.2 M, Hepes 0.1 M pH7.5, and were mounted in a fiber loop and flash-cooled under a nitrogen flux after cryoprotection with Na_2_ Tartrate 1.1 M, MgCl_2_ 0.2 M, Hepes 0.1 M pH7.5, Glycerol 25%. Data collection from a single frozen crystal was performed at 100 K on the PX2 at SOLEIL synchrotron (France). The raw data were processed with XDS^55^ and scaled with AIMLESS^56^ programs. The crystals belong to the space group *P*6_5_22, with one LBD complex per asymmetric unit. The structures were solved and refined using Phenix,^57^ Buster^58^ and iterative model building using COOT.^59^ Crystallographic refinement statistics are presented in Table S2.

#### Chromatin Immunoprecipitation (ChIP)

B16–F10 cells were treated with DMSO, 1,25D or AC-340 for 2 or 6 h, depending on the protein of interest. Cells were then fixed by adding formaldehyde directly to the culture medium to a final concentration of 1% and incubated for 15 min at RT. To quench the fixation, glycine was added to the medium at a final concentration of 125 mM. Cells were then washed twice with cold PBS followed by solubilizing in lysis buffer (1% SDS, 50 mM Tris-HCl pH = 8, 10 mM EDTA) supplemented with 1% protease inhibitor cocktail. After scraping the cells and incubating for 30 min on ice, the lysates were sonicated on a Bioruptor with a repeated cycle of 10 s ON/10 s OFF, 6 times, to obtain chromatin fragments <600 bp. To remove cellular debris, the samples were centrifuged for 10 min at 10,000 rpm at 4 °C. Each sample was divided into 3 parts; 150 μL supernatant diluted 1:10 in ChIP dilution buffer (1% Triton X-100, 1 mM EDTA, 17 mM Tris-HCl pH = 8, 334 mM NaCl) supplemented with 1% protease inhibitor cocktail was incubated with Ab or nonspecific rabbit IgG (Table S4) overnight at 4 °C on a rotating platform. Ten μL was stored at −20 °C as input control. The next day, immune complexes were collected using Dynabeads Protein G (Invitrogen- #10003D) for 2 h at 4 °C with rotation. The beads were then washed twice, 5 min each, with the following buffers: TSE I (low salt; 0.1% SDS, 1% Triton X-100, 2 mM EDTA, 20 mM Tris-HCl pH = 8, 150 mM NaCl), TSE II (high salt; 0.1% SDS, 1% Triton X-100, 2 mM EDTA, 20 mM Tris-HCl pH = 8, 500 mM NaCl), Lithium Chloride (250 mM LiCl, 1% IGEPAL, 1 mM EDTA, 10 mM Tris-HCl pH = 8, 24 mM Deoxycholic acid) and TE (10 mM Tris-HCl pH = 8, 1 mM EDTA). Immune complexes were then eluted using 200 μL elution buffer (1% SDS, 100 mM NaHCO3) followed by reverse cross-linking for 4 h at 65 °C in the presence of 200 mM NaCl. To eliminate proteins, samples were shaken for another hour at 45 °C in the presence of proteinase K (Fisher Scientific- #P8107S). Finally, DNA was purified using the GEL/PCR purification mini kit (FAVORGEN Biotech Corp. #FAGCK 001-1). Quantification of immunoprecipitated materials was performed by qPCR and the enrichment of the target gene VDRE in each condition was normalized to the percentage input. ChIP-qPCR primer sequences are listed in Table S3.

#### Statistical Analyses

Statistics were calculated using GraphPad Prism (version 8). A parametric unpaired *t*-test was used for comparison of two groups in all in vitro experiments. Statistical significance is indicated as follows: *p < 0.05, **p < 0.01, ***p < 0.001, and ****p < 0.0001.

## Supplementary Material







## Notes

Additional figures illustrating NMR spectra, HRMS, and HPLC reports from synthesized compounds, bifunctionality assessments of AC-340 in human melanoma cells in vitro, dose response profile for inhibition of zinc-dependent HDACs by AC-340, RNaseq workflow and volcano plots demonstrating the magnitude of gene regulation in each treatment condition, RNaseq data validation by RT-qPCR, ChIP-qPCR data showing the recruitment of VDR and coactivator CBP to the mouse *Vdr* gene along with the acetylation level of H3K27 in 1,25D- or AC-340-treated B16–F10 cell, crystallographic data such as AC-340 modeled into the omit Polder maps and protein–ligand distances observed in the AC- or 1,25D-zVDR LBD complexes, list of primers used for RT-qPCR and ChIP-qPCR, and antibodies used for Western blots and ChIP assays (PDF)
Significant DEGs compiled (XLSX)
Molecular formula strings (CSV)

## Notes

Atomic coordinates for the X-ray structure of zVDR LBD-AC-340 (PDB 9RCG) are available from the RCSB Protein Data Bank.

## Abbreviations

1,25D: 1α,25-dihydroxyvitamin D
25D: 25-hydroxyvitamin D
Aqp1: aquaporin1
CBP: Creb binding protein
ChIP: chromatin immunoprecipitation
C NMR: carbon nuclear magnetic resonance
CYP24A1: cytochrome P450 family 24 subfamily A member 1
CYP27B1: cytochrome P450 family 27 subfamily B member 1
DEG: differentially expressed genes
DMEM: Dulbecco’s Modified Eagle Medium
ECL: enhanced chemiluminescence
EMEM: Eagle’s Minimum Essential Medium
FBS: fetal bovine serum
GAPDH: glyceraldehyde 3-phosphate dehydrogenase
HDACi: histone deacetylase inhibitor(s)
H NMR: proton nuclear magnetic resonance
HRP: horseradish peroxidase
LBD: ligand binding domain
NCoA2: nuclear receptor coactivator 2
PBS: phosphate buffer saline
Prelp: proline and arginine rich end leucine rich repeat protein
rmsd: root-mean-square deviation
RT: room temperature
RT-qPCR: reverse transcription-quantitative Polymerase chain reaction
SAHA: suberoylanilide hydroxamic acid
Spp1: osteopontin
TBS: tris-buffered saline
Tgm2: transglutaminase 2
THF: tetrahydrofuran
TLC: thin-layer chromatography
TSA: trichotomic A
VDR: vitamin D receptor
VDRE: vitamin D receptor element
