# Supplementary material for: Hyperagonism of a Vitamin D Receptor Agonist/Histone Deacetylase Inhibitor Hybrid Molecule
Source: J Med Chem. 2025 Sep 18;68(19):20675–88. doi: 10.1021/acs.jmedchem.5c01932 (PMC12517324; doi:10.1021/acs.jmedchem.5c01932)
Supplement: Supplementary file 1 [file jm5c01932_si_001.pdf]

Supporting Information

Hyperagonism of a vitamin D receptor agonist/histone deacetylase inhibitor hybrid molecule

Fatemeh Sarmadi[a], Amelia Caza[b], Zhizhong Gao[b], Natacha Rochel[c], James L. Gleason[b]*, John H. White[a,d]*

[a] *Department of Physiology, McGill University, 3655 Promenade Sir William Osler, Montreal, QC, H3G 1Y6, Canada*
[b] *Department of Chemistry, McGill University, 801 Sherbrooke W., Montreal, QC, H3A 0B8, Canada*
[c] *Department of Integrative and Structural Biology, Institut de Génétique et de Biologie Moléculaire et Cellulaire, Université de Strasbourg, CNRS, Inserm, UMR 7104-UMR-S 1258, F-67400 Illkirch, France.*
[d] *Department of Medicine, McGill University, 3655 Promenade Sir William Osler, Montreal, QC, H3G 1Y6, Canada*

* Address correspondence to J.H.W. for molecular/cell biology: john.white@mcgill.ca
Address correspondence to J.L.G. for chemistry: jim.gleason@mcgill.ca

Contents

NMR spectra and HRMS for all new compounds synthesized 2
HPLC reports of 7a (AC-340), 7b (AC-339), and 7c (AC-341) 53
Bifunctionality of AC-340 in human melanoma cells *in vitro* 71
Dose response profiles for inhibition of different HDACs by AC-340 72
RNAseq workflow and volcano plots demonstrating the magnitude of gene regulation in each treatment condition 73
RNAseq data validation by RT-qPCR of selected VDR target genes in B16-F10 cells 74
ChIP-qPCR data showing the recruitment of VDR and coactivator CBP to the mouse *Vdr* gene along with the acetylation level of H3K27 in 1,25D- or AC-340-treated B16-F10 cells 75
AC-340 modeled into the omit Polder maps 76
Protein-ligand distances observed in the AC- or 1,25D-zVDR LBD complexes 77
Crystallographic data and refinement 78
Primers used for RT-qPCR and ChIP-qPCR 79
Antibodies used for western blots and ChIP assays 79

NMR spectra and HRMS for all new compounds synthesized

^{1}H NMR of **13** (500 MHz, $CDCl_3$)

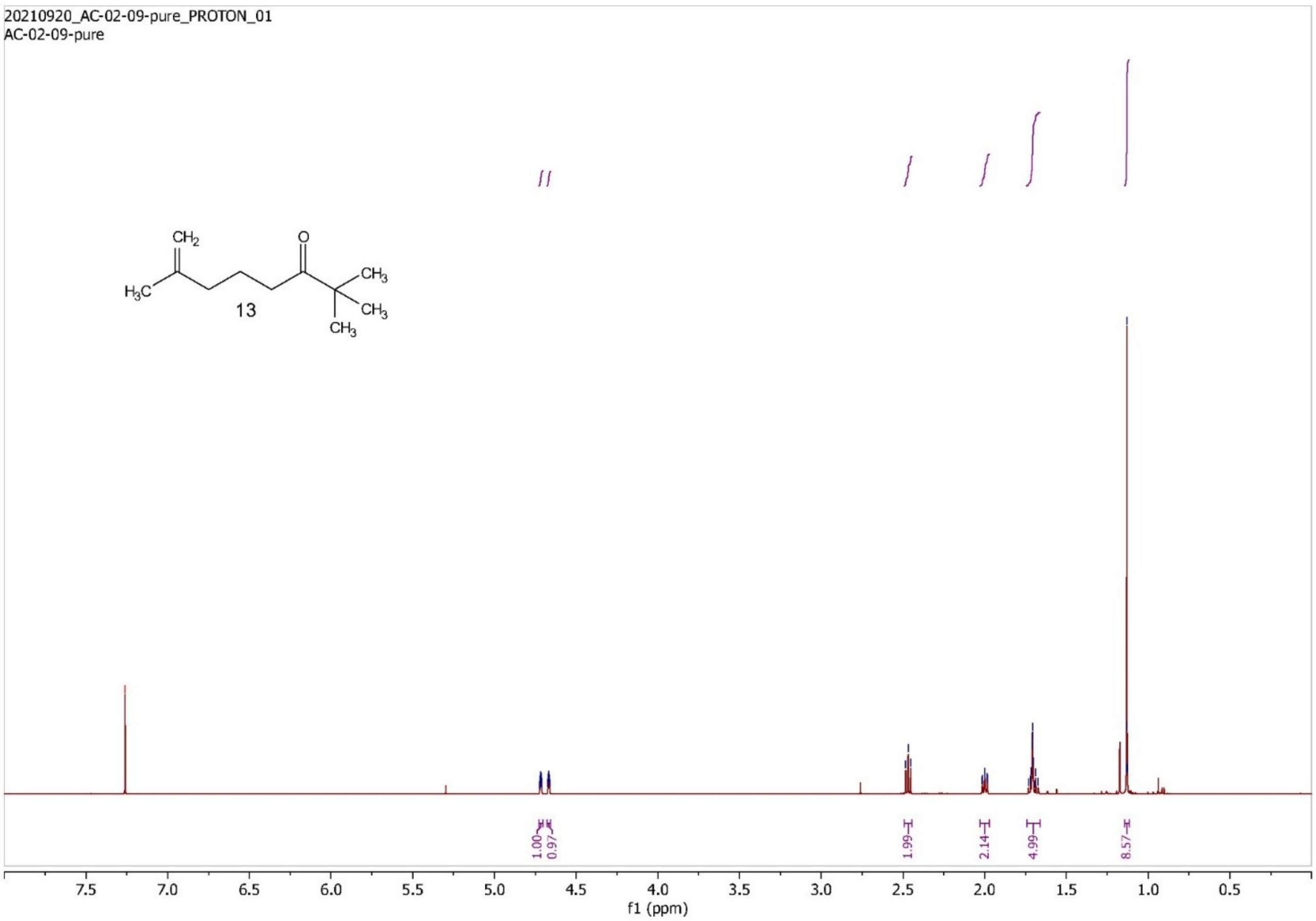

^{13}C NMR of **13** (126 MHz, $CDCl_3$)

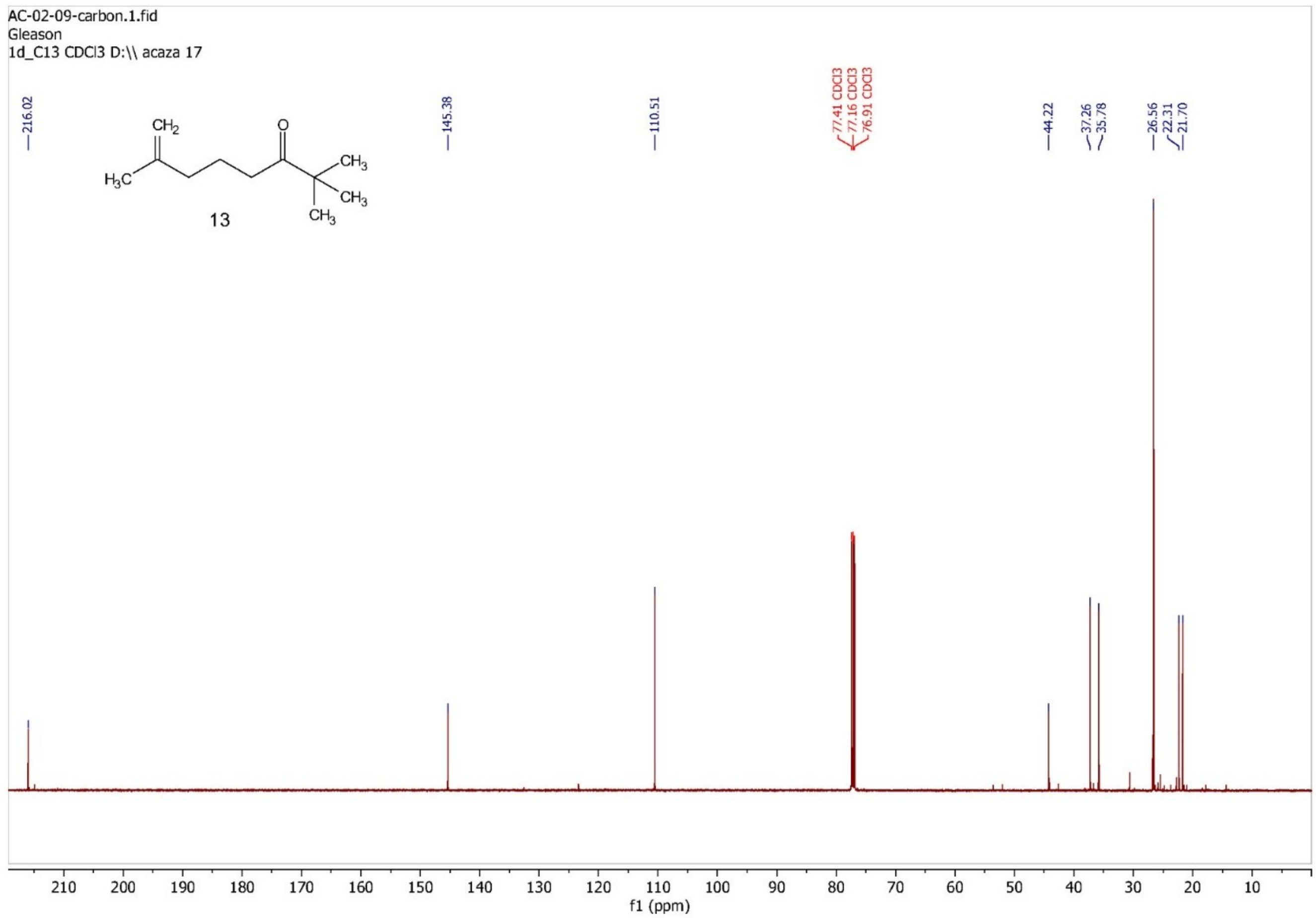

HRMS of **13**

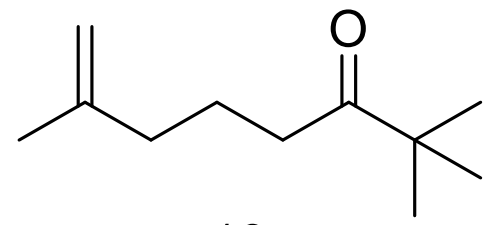

13

220913-01ESI- HRMS-Gleason-   09/13/22 11:16:28

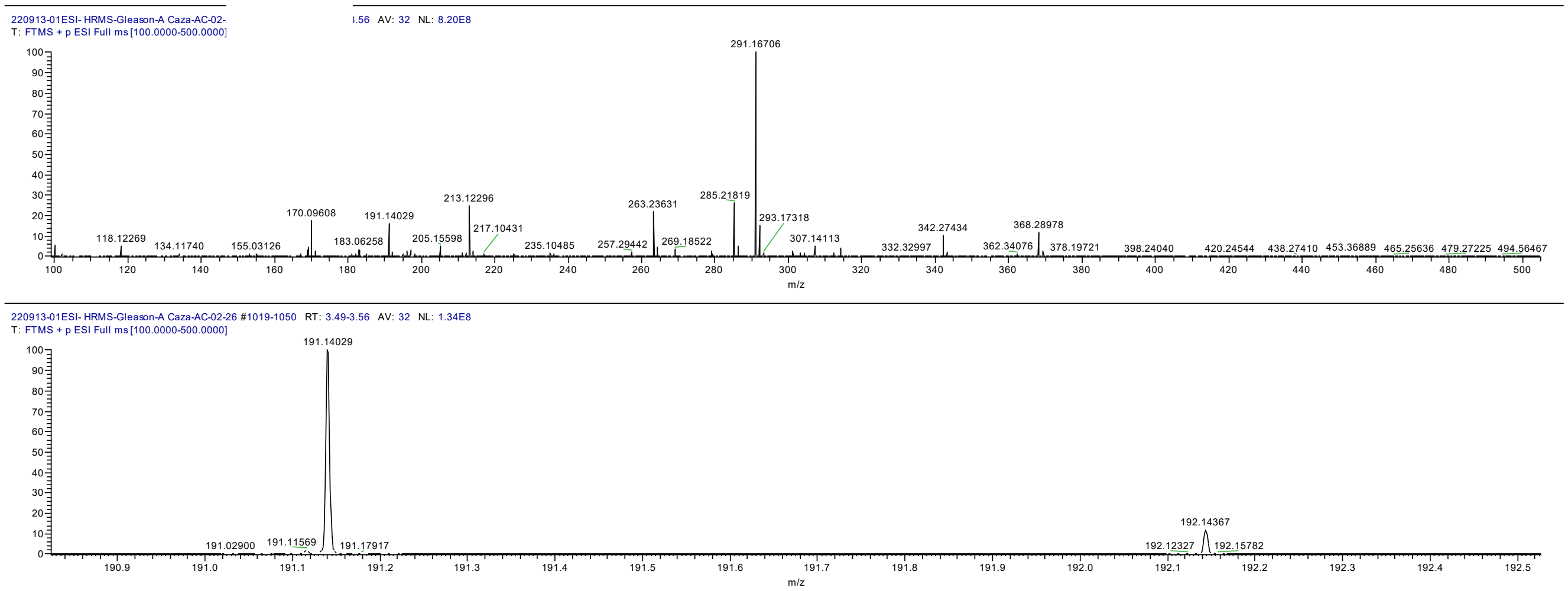

220913-01ESI- HRMS-Gleason-A Caza-AC-02-26#1019-1050 RT: 3.49-3.56 AV: 32
T: FTMS + p ESI Full ms [100.0000-500.0000]
m/z= 191.13564-191.14577

m/z	Intensity	Relative	Resolution	Charge	Theo. Mass	Delta (ppm)	RDB equiv.	Composition
191.14029	138542208.0	100.00	38244.67	1.00	191.14064	-1.83	1.5	$C_{11}H_{20}ONa$

^{1}H NMR of **14** (500 MHz, $CDCl_3$)

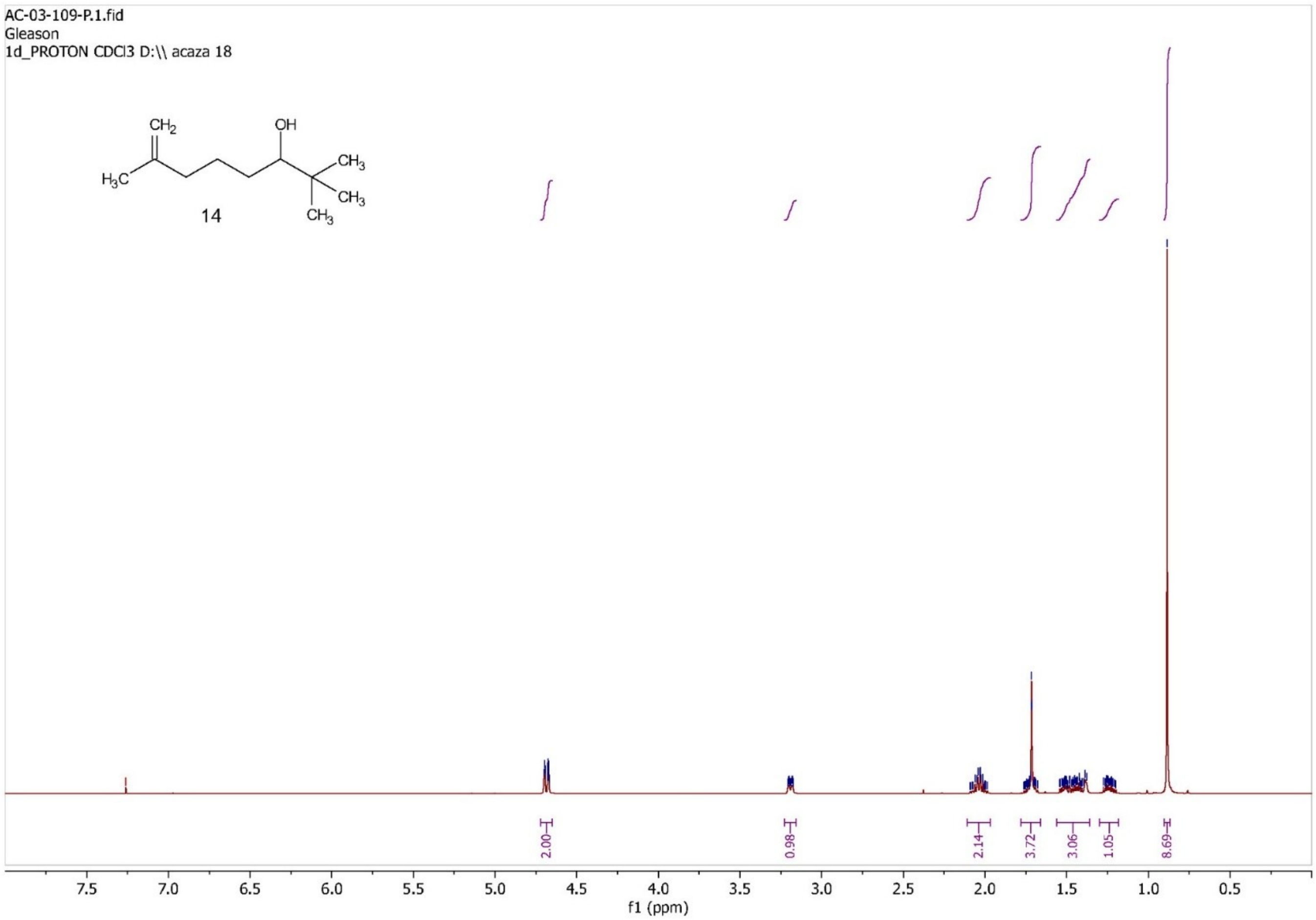

^{13}C NMR of **14** (126 MHz, $CDCl_3$)

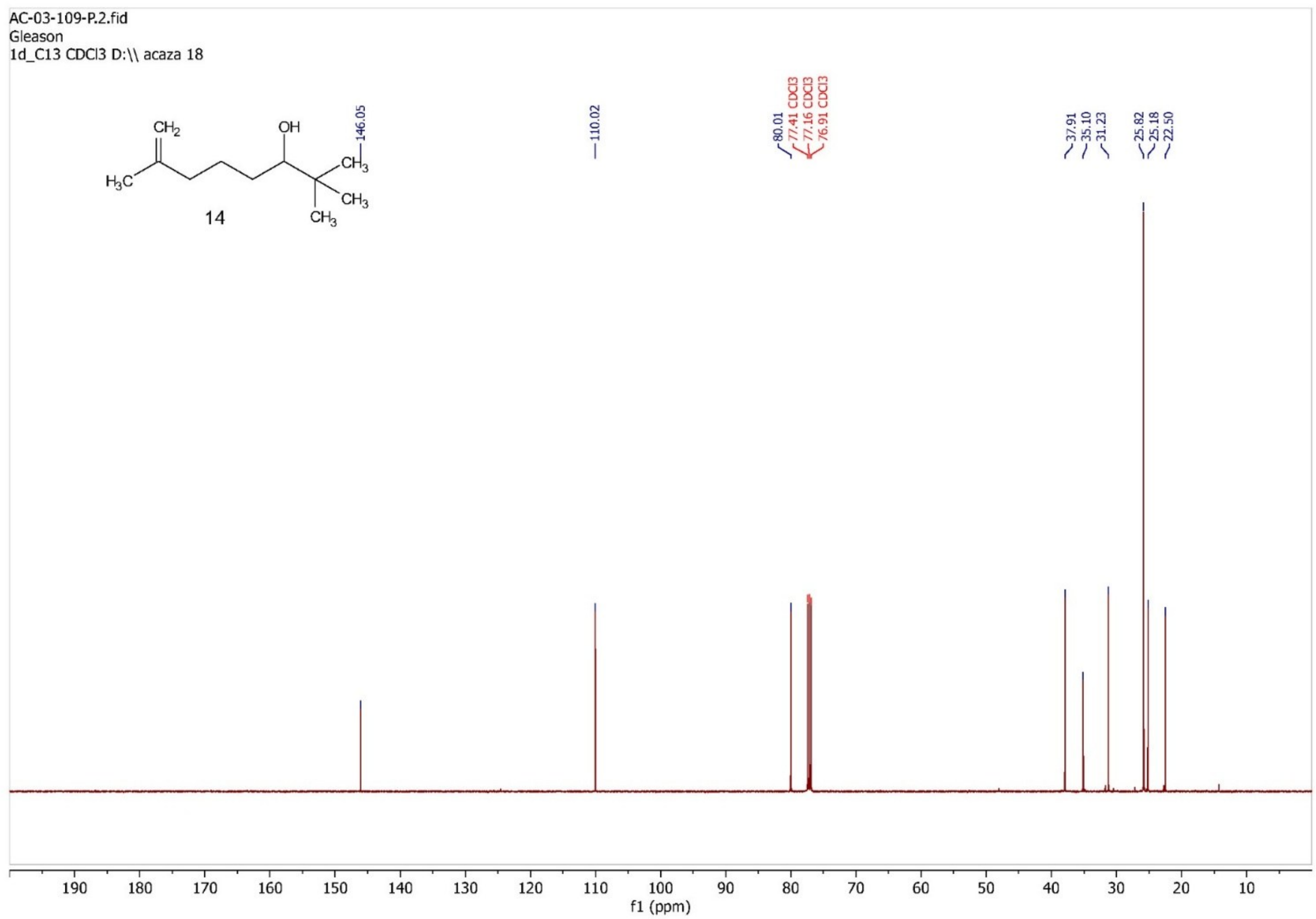

HRMS of **14**

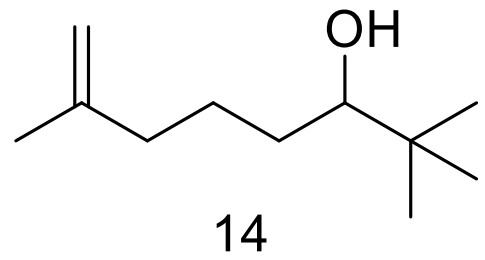

14

Mass Spectrum SmartFormula Report

Analysis Info

Analysis Name	D:\Data\Gleason\2023-01-05 Gleason-Caza AC-03-109 ESI +ve.d	Acquisition Date	1/5/2023 12:02:44 PM
Method	Tune_pos_Low_Na_Formate_100-1000.m	Operator	Alex
Sample Name	2023-01-05 Gleason-Caza AC-03-109 ESI +ve	Instrument	maXis impact 282001.00044
Comment			

Acquisition Parameter

Source Type	ESI	Ion Polarity	Positive	Set Nebulizer	0.6 Bar
Focus	Not active	Set Capillary	3500 V	Set Dry Heater	180 °C
Scan Begin	100 m/z	Set End Plate Offset	-500 V	Set Dry Gas	4.0 l/min
Scan End	1000 m/z	Set Charging Voltage	2000 V	Set Divert Valve	Waste
		Set Corona	0 nA	Set APCI Heater	0 °C

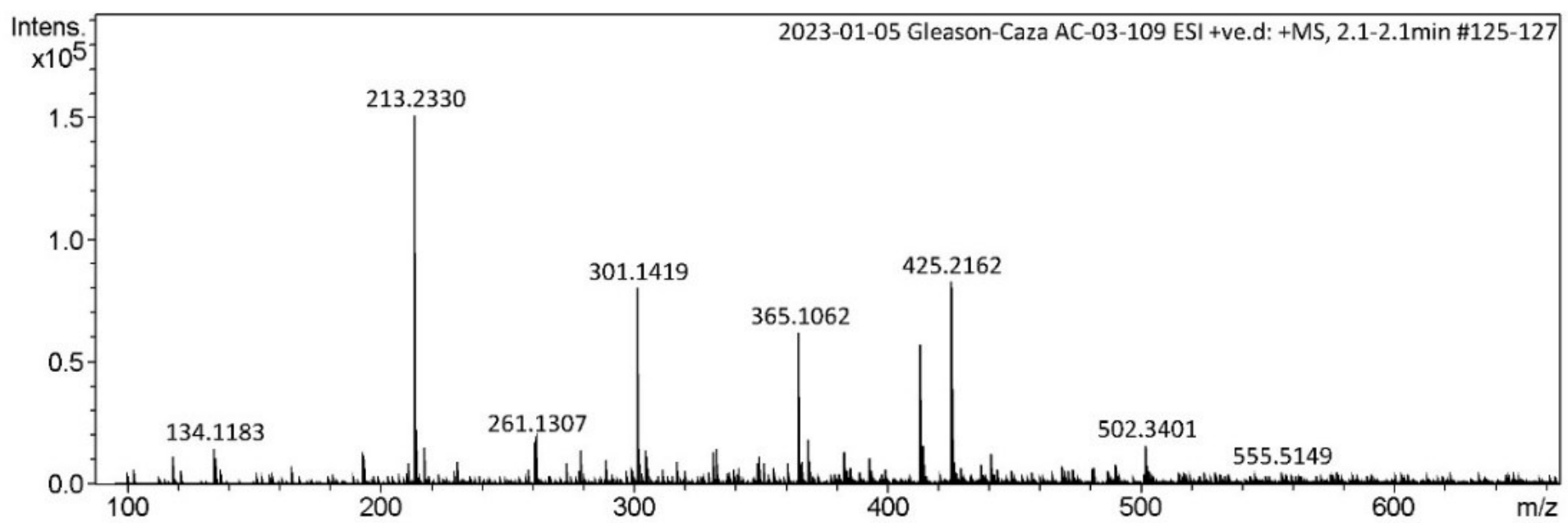

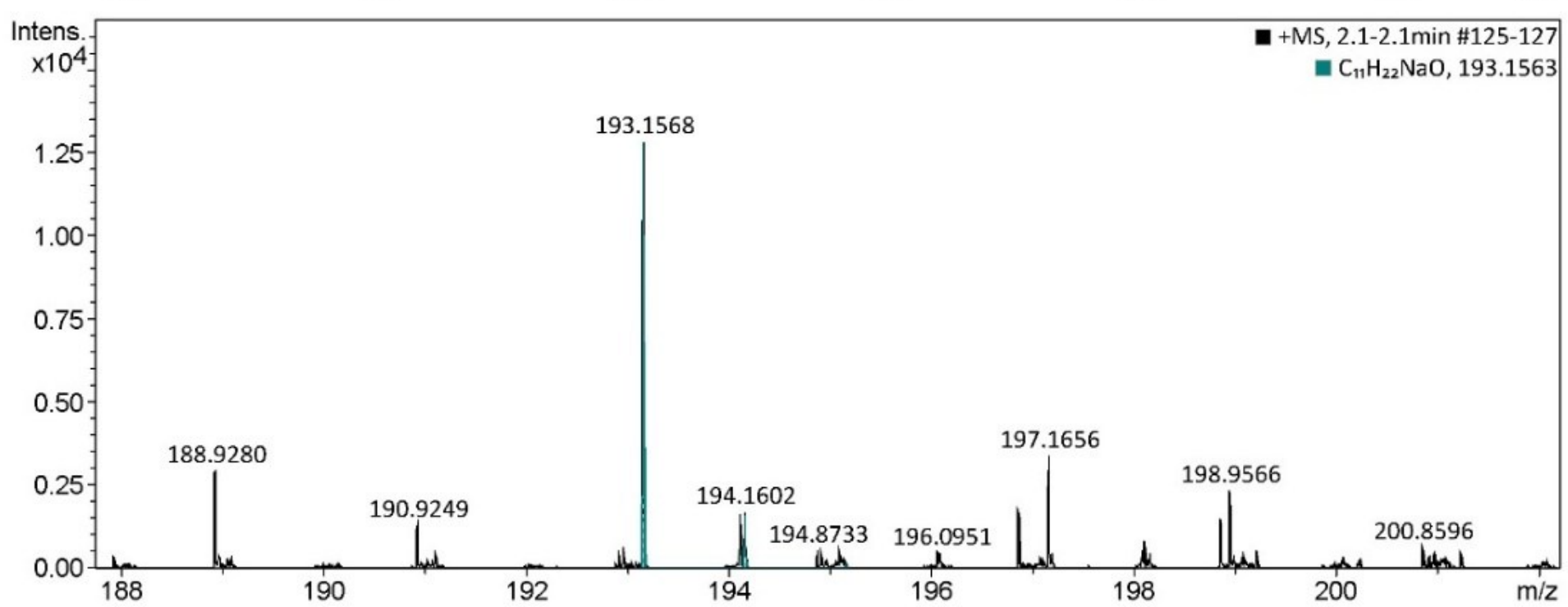

Meas. m/z	#	Ion Formula	m/z	err [ppm]	mSigma	# mSigma	Score	rdb	e⁻ Conf	N-Rule
193.1568	1	C11H22NaO	193.1563	-2.5	5.5	1	100.00	0.5	even	ok

2023-01-05 Gleason-Caza AC-03-109 ESI +ve.d

Bruker Compass DataAnalysis 4.2 printed: 1/5/2023 5:18:19 PM by: Alex Page 1 of 1

^{1}H NMR of **15** (500 MHz, $CDCl_3$)

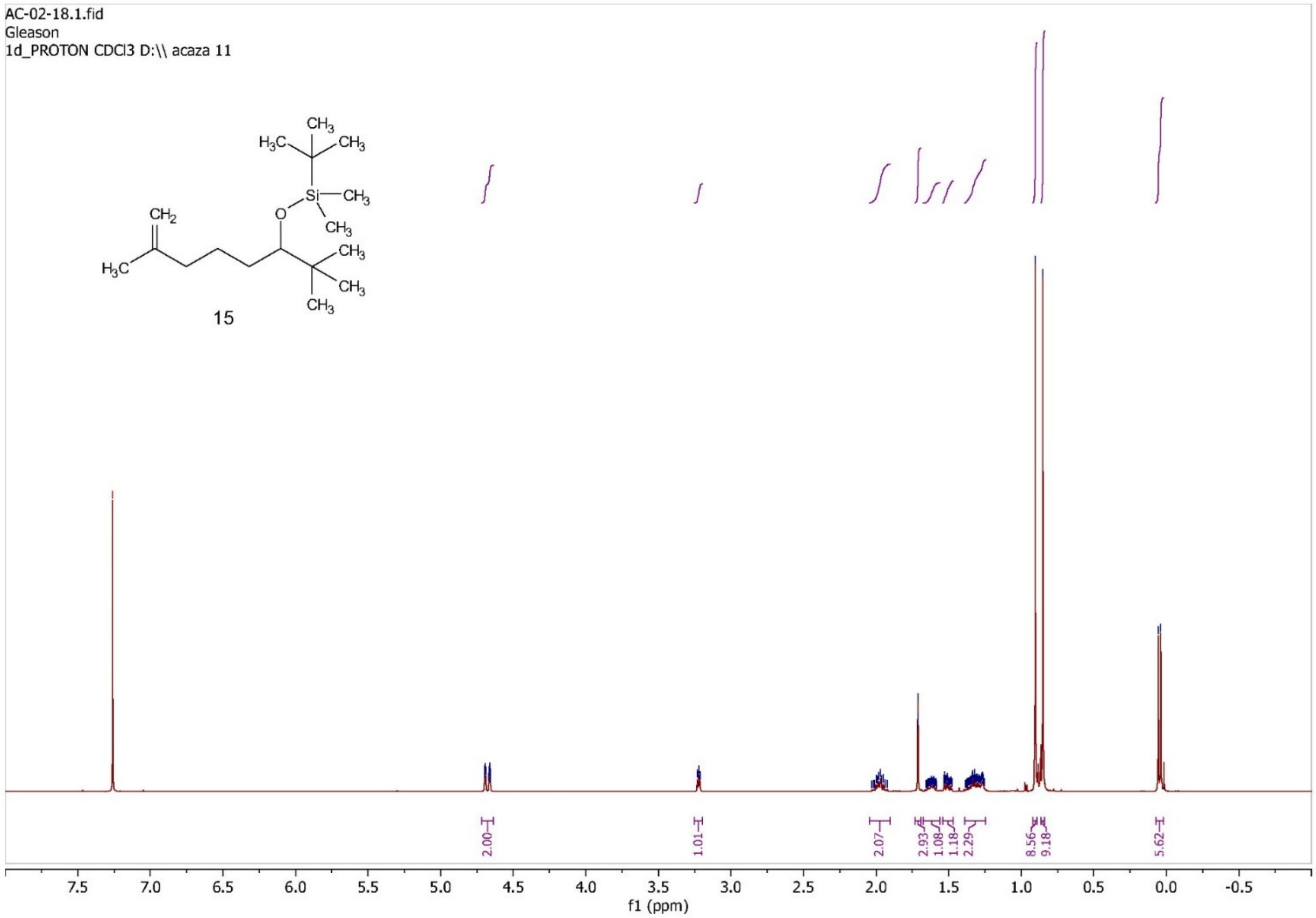

^{13}C NMR of **15** (126 MHz, $CDCl_3$)

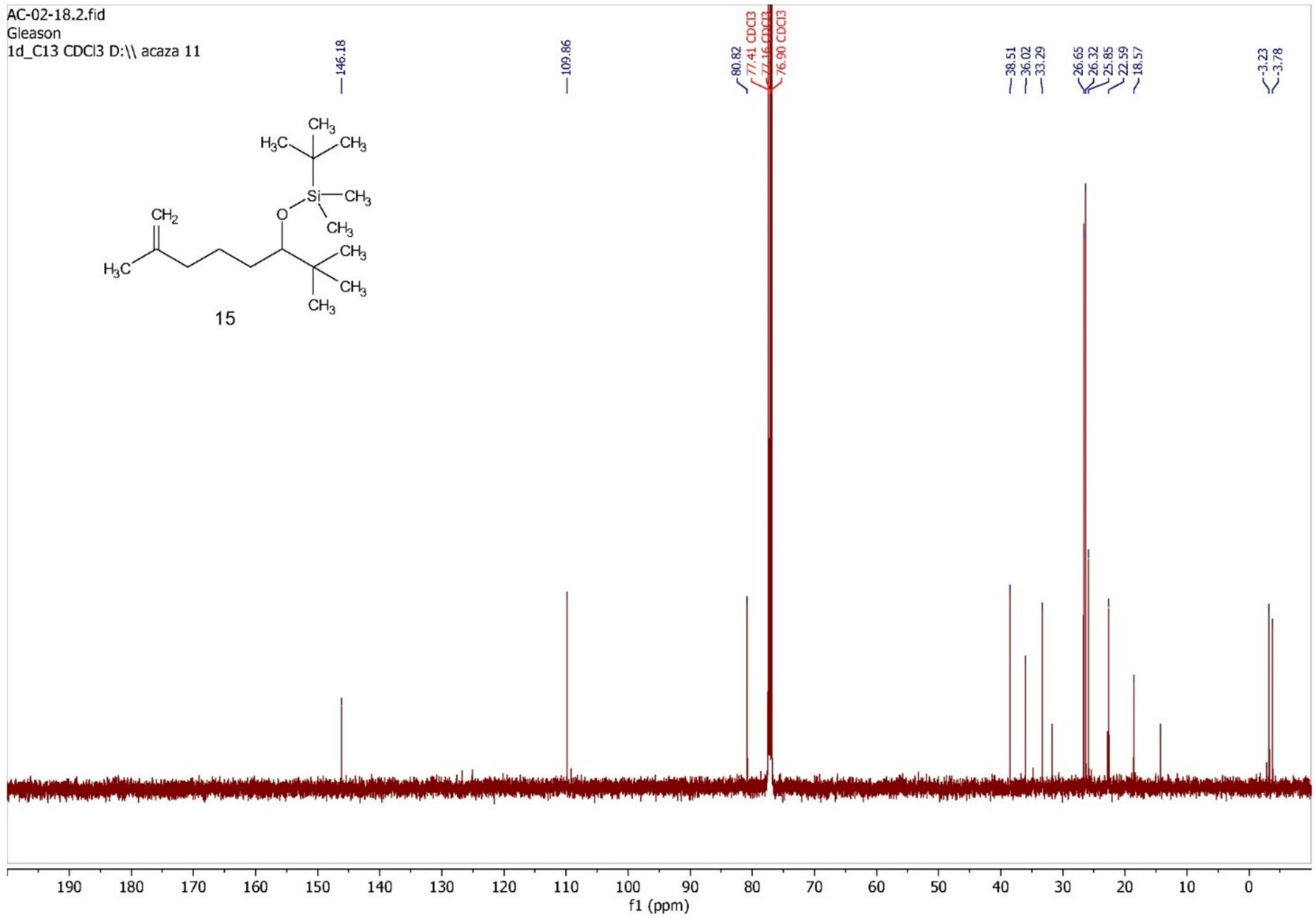

HRMS of **15**

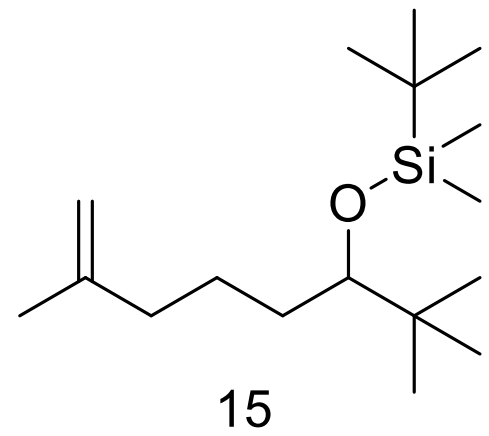

15

Mass Spectrum SmartFormula Report

Analysis Info

Analysis Name	D:\Data\Gleason\2023-01-05 Gleason-Caza AC-03-110 ESI +ve.d	Acquisition Date	1/5/2023 11:56:56 AM
Method	Tune_pos_Low_Na_Formate_100-1000.m	Operator	Alex
Sample Name	2023-01-05 Gleason-Caza AC-03-110 ESI +ve	Instrument	maXis impact 282001.00044
Comment			

Acquisition Parameter

Source Type	ESI	Ion Polarity	Positive	Set Nebulizer	0.6 Bar
Focus	Not active	Set Capillary	3500 V	Set Dry Heater	180 °C
Scan Begin	100 m/z	Set End Plate Offset	-500 V	Set Dry Gas	4.0 l/min
Scan End	1000 m/z	Set Charging Voltage	2000 V	Set Divert Valve	Waste
		Set Corona	0 nA	Set APCI Heater	0 °C

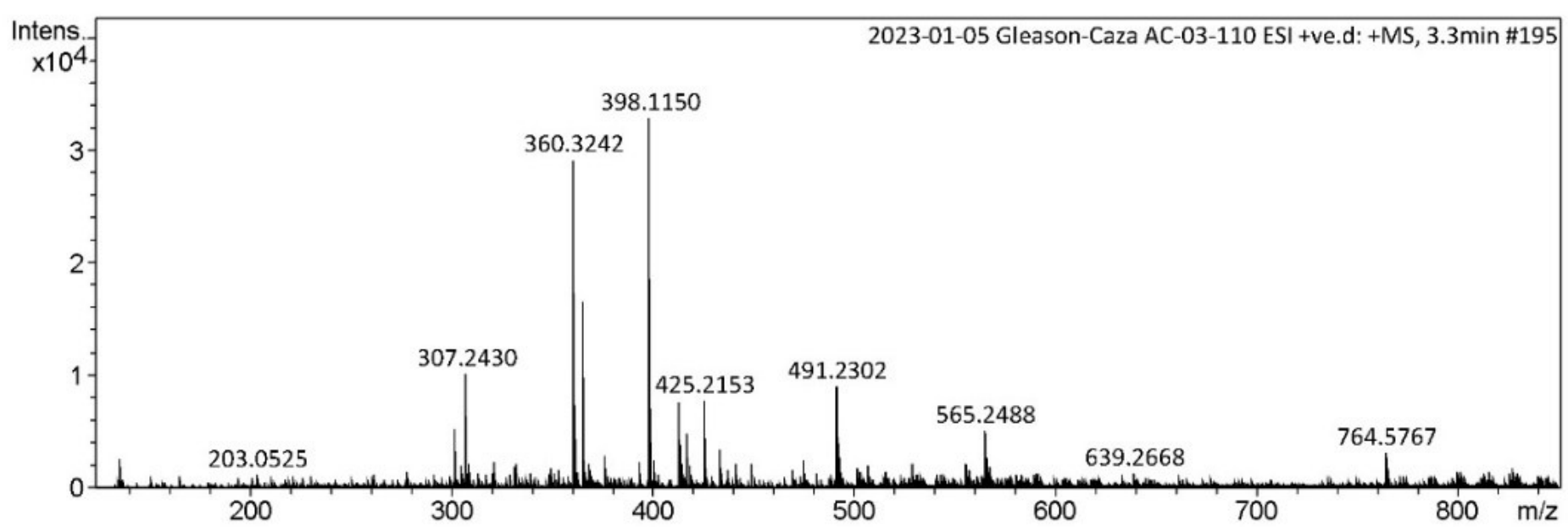

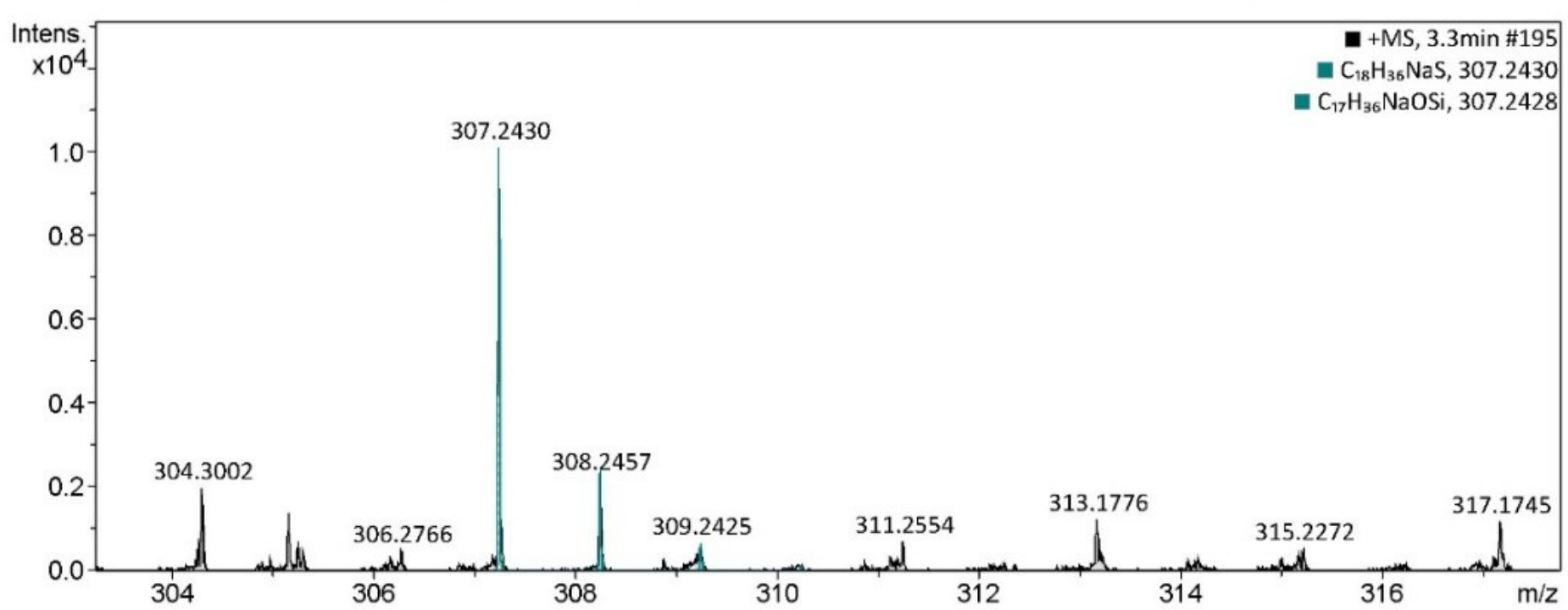

Meas. m/z	#	Ion Formula	m/z	err [ppm]	mSigma	# mSigma	Score	rdb	e⁻ Conf	N-Rule
307.2430	1	C17H36NaOSi	307.2428	-0.8	11.0	1	100.00	0.5	even	ok

2023-01-05 Gleason-Caza AC-03-110 ESI +ve.d

Bruker Compass DataAnalysis 4.2 printed: 1/5/2023 4:57:53 PM by: Alex Page 1 of 1

^{1}H NMR of **16** (500 MHz, $CDCl_3$)

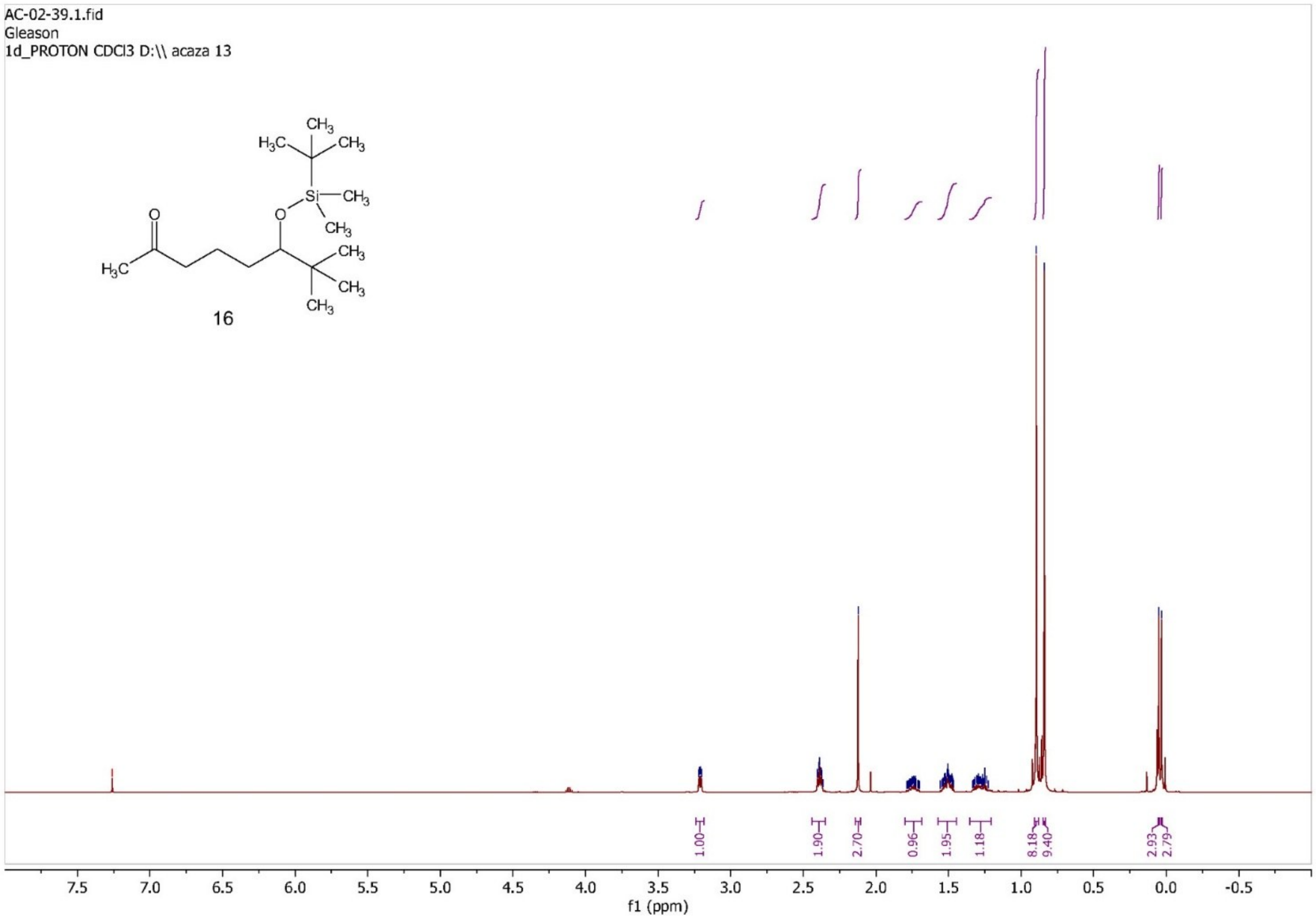

^{13}C NMR of **16** (126 MHz, $CDCl_3$)

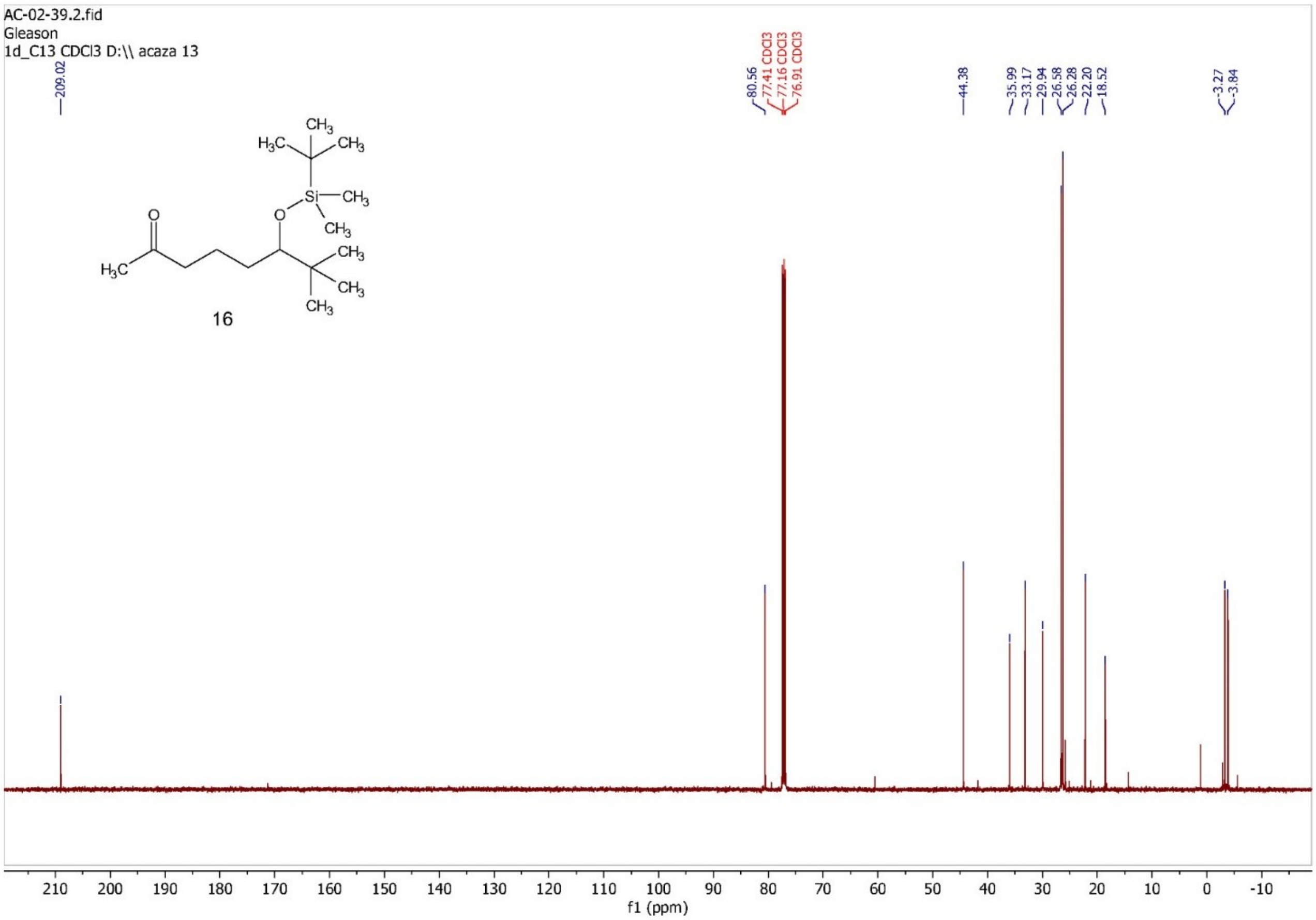

HRMS of **16**

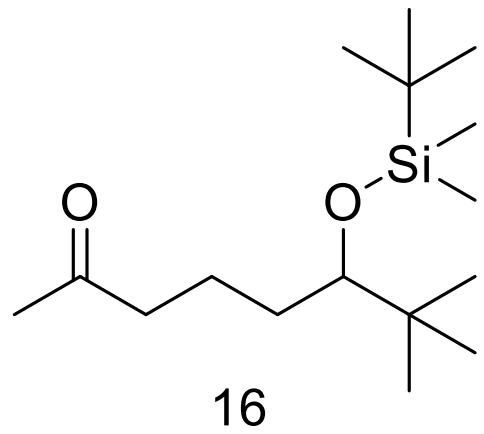

16

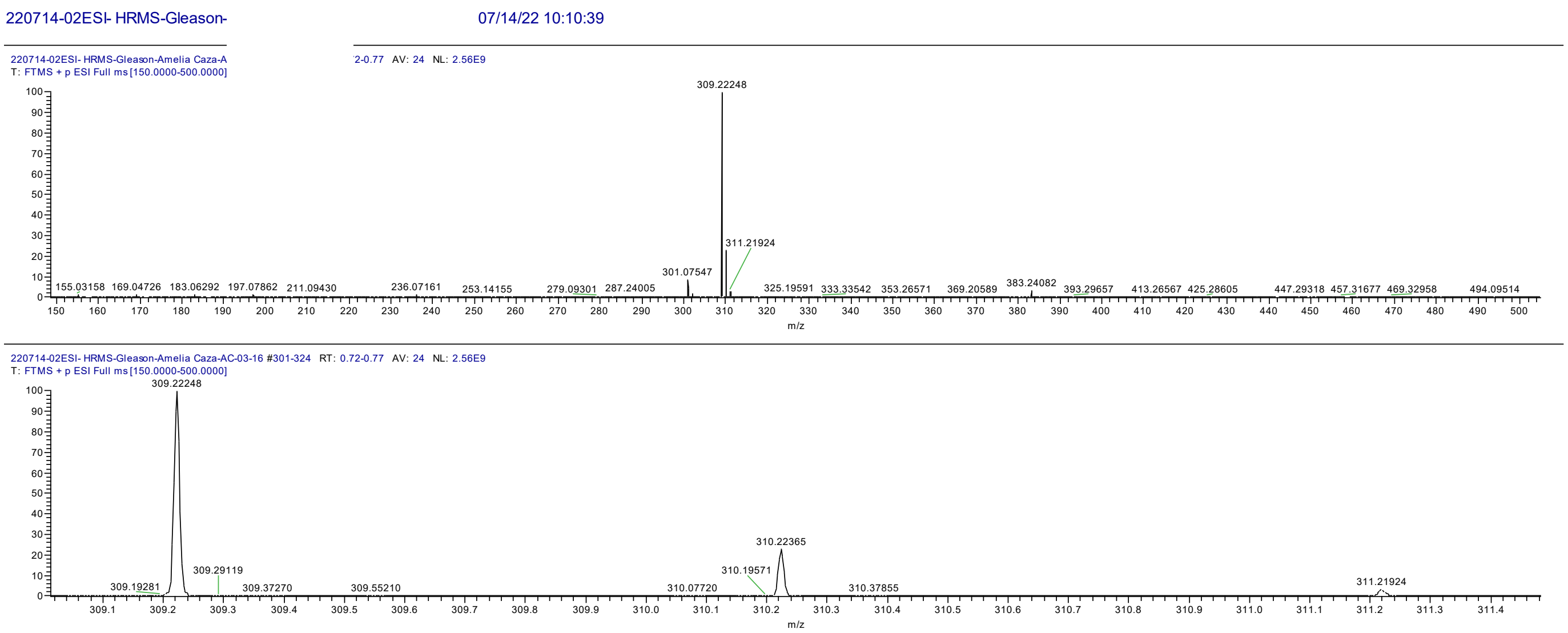

220714-02ESI- HRMS-Gleason-Amelia Caza-AC-03-16#301-324 RT: 0.72-0.77 AV: 24
T: FTMS + p ESI Full ms [150.0000-500.0000]
m/z= 309.20881-309.23168

m/z	Intensity	Relative	Resolution	Charge	Theo. Mass	Delta (ppm)	RDB equiv.	Composition
309.22248	2579961856.0	100.00	30496.36	1.00	309.22203	1.47	0.5	$C_{16}H_{34}O_2NaSi$

^{1}H NMR of **17a** (500 MHz, $CDCl_3$)

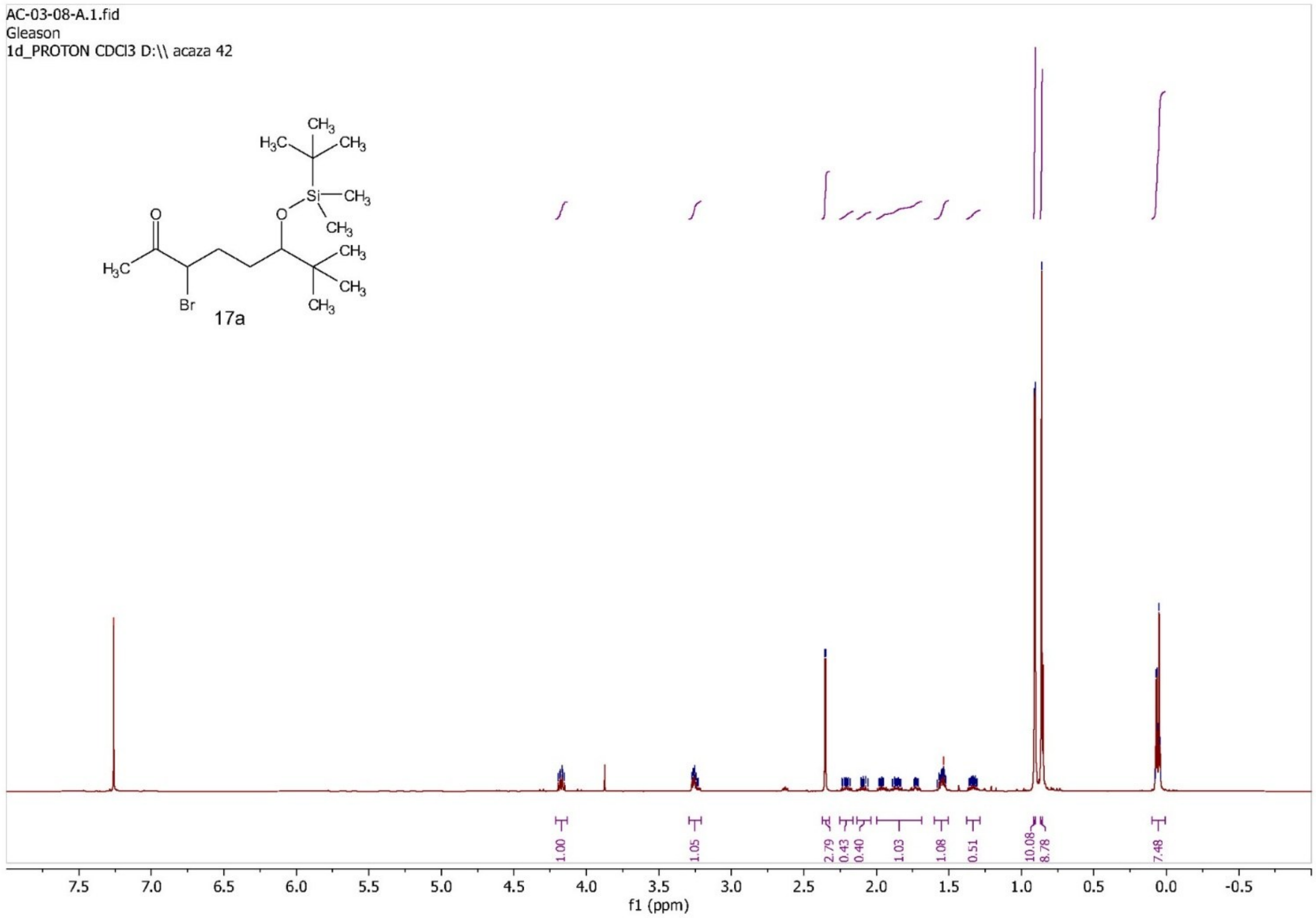

^{13}C NMR of **17a** (126 MHz, $CDCl_3$)

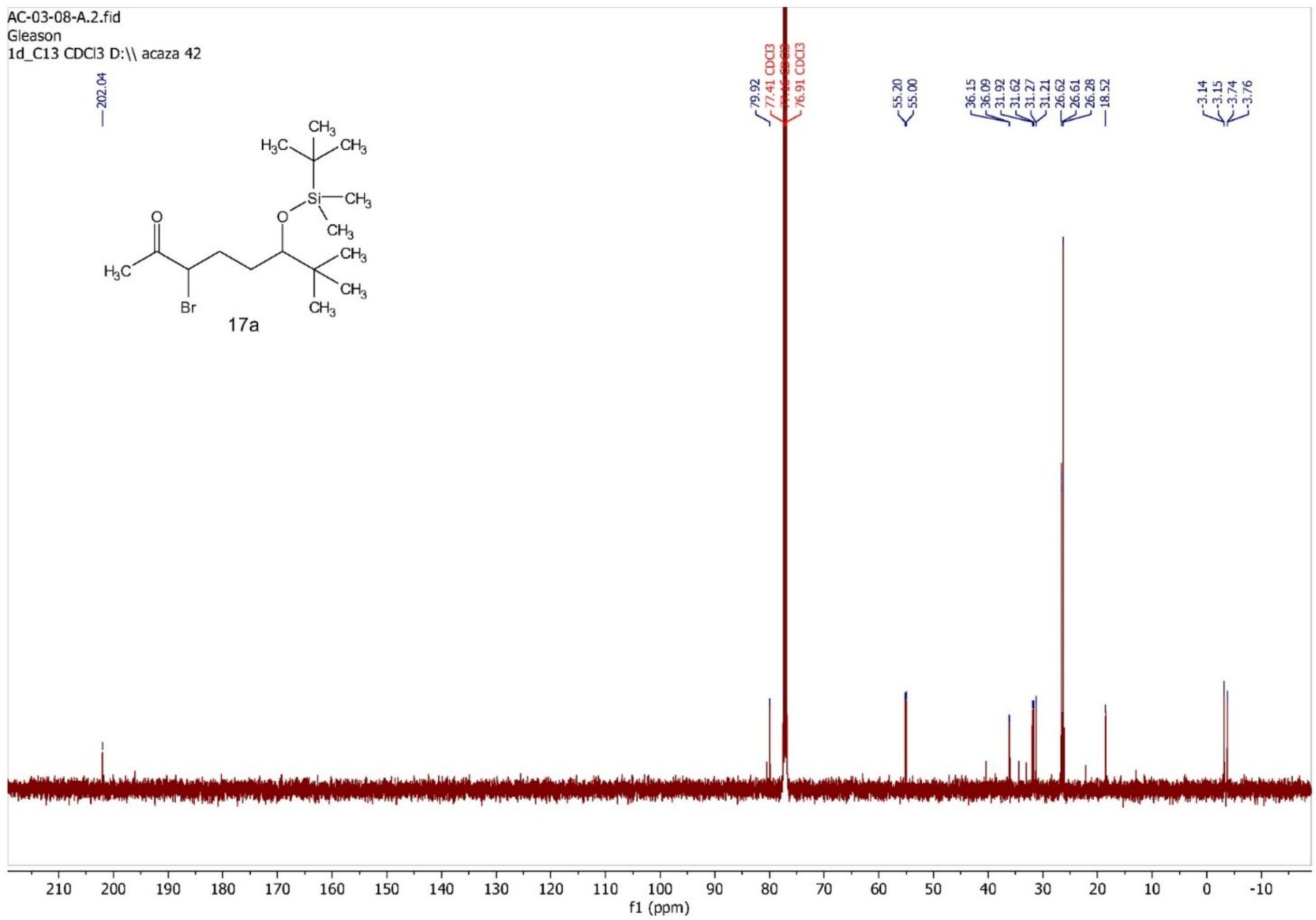

HRMS of **17a**

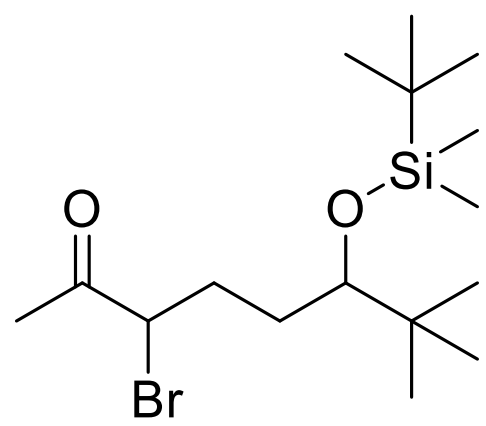

17a

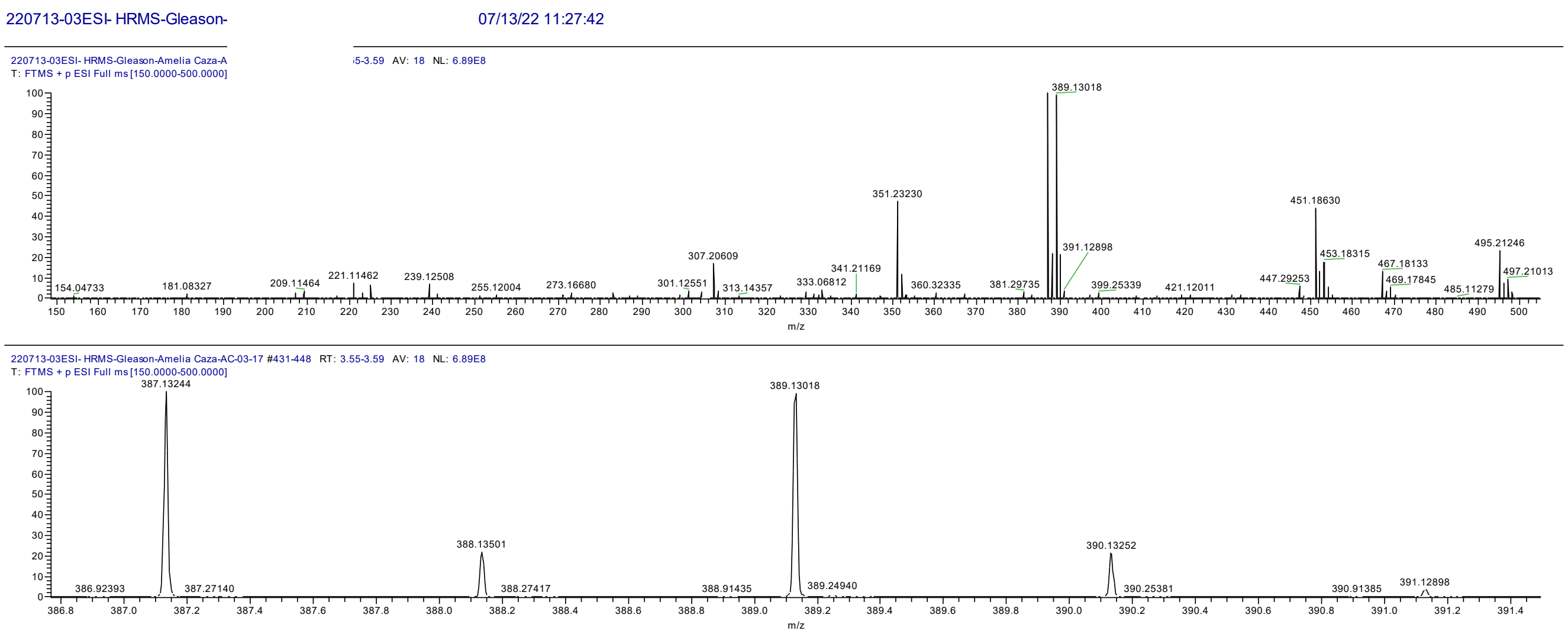

220713-03ESI- HRMS-Gleason-Amelia Caza-AC-03-17#431-448 RT: 3.55-3.59 AV: 18
T: FTMS + p ESI Full ms [150.0000-500.0000]
m/z= 387.11936-387.14439

m/z	Intensity	Relative	Resolution	Charge	Theo. Mass	Delta (ppm)	RDB equiv.	Composition
387.13244	694367424.0	100.00	28118.37	1.00	387.13254	-0.25	0.5	$C_{16}H_{33}O_2BrNaSi$
					387.13409	-4.26	0.5	$C_{16}H_{30}O_3BrF_2$

^{1}H NMR of **8** (500 MHz, $CDCl_3$)

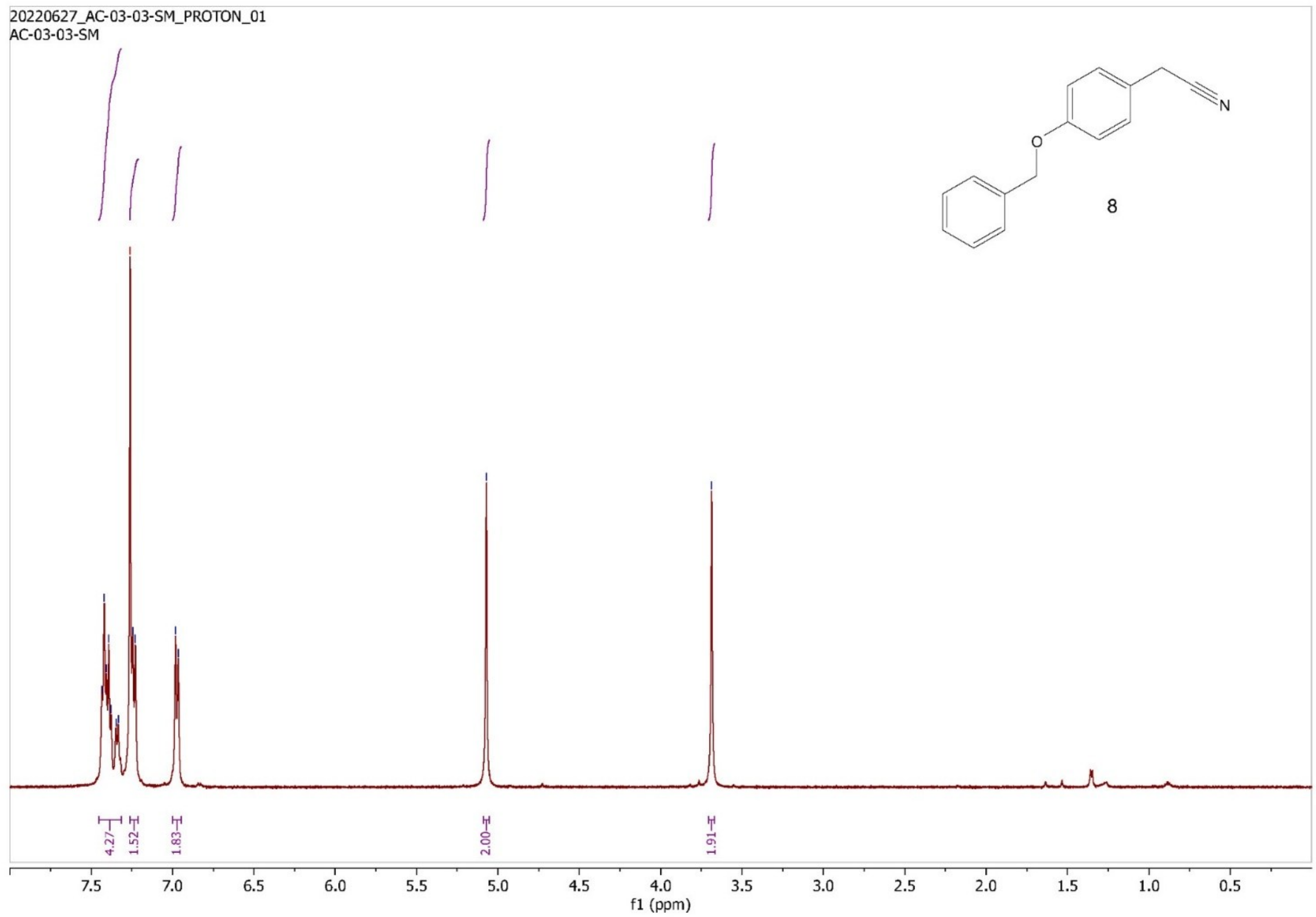

^{1}H NMR of **9** (500 MHz, $CDCl_3$)

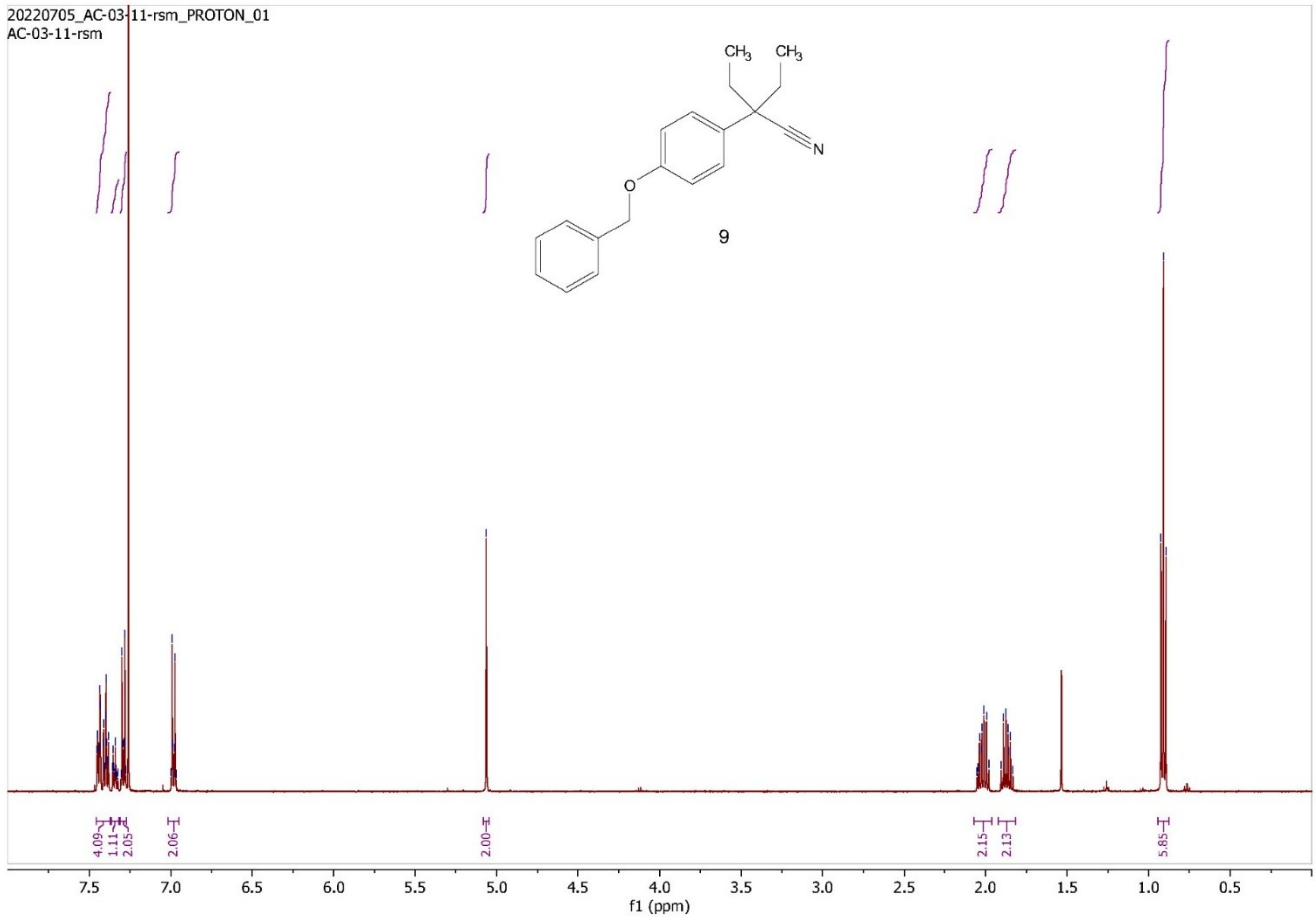

^{13}C NMR of **9** (126 MHz, $CDCl_3$)

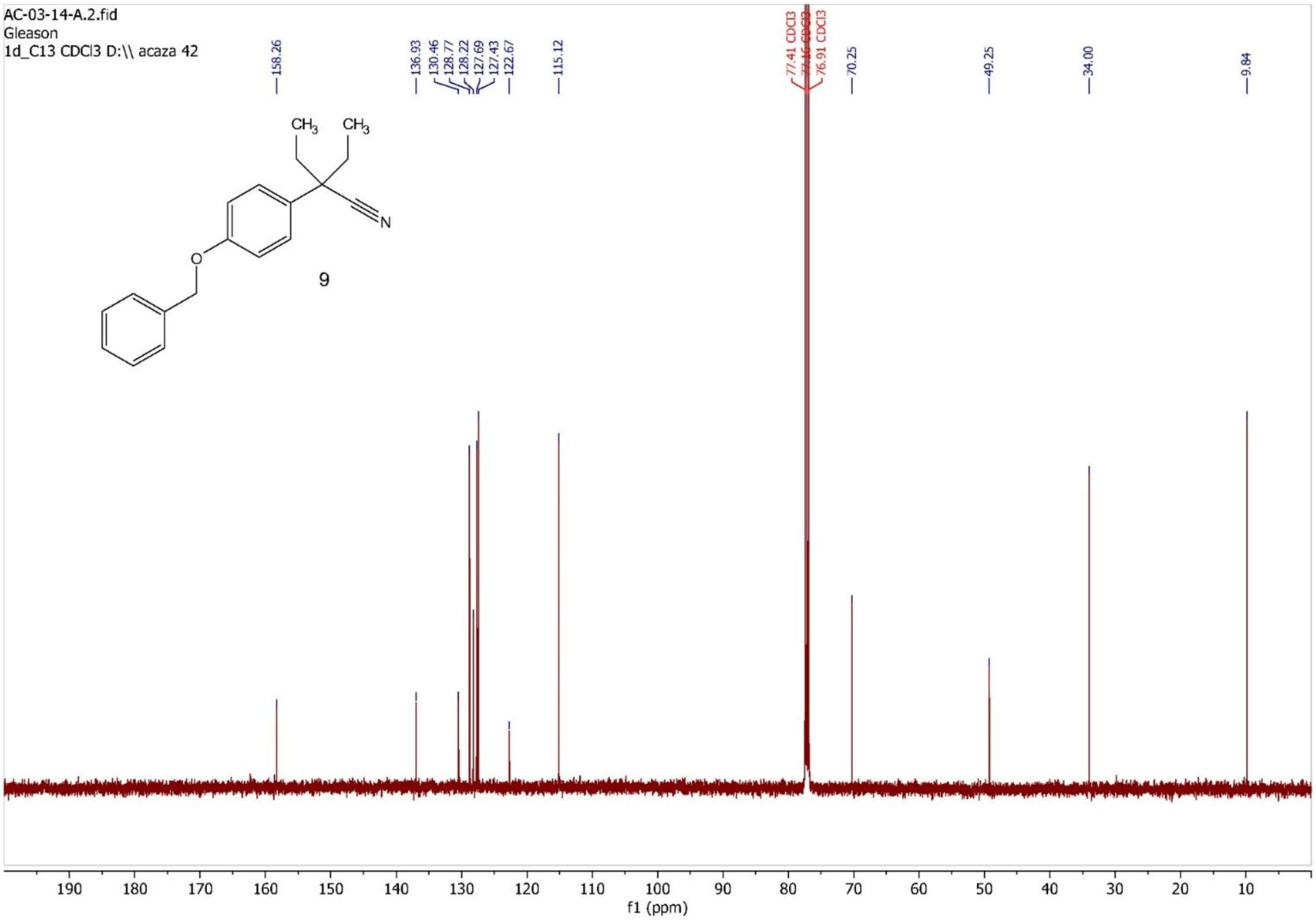

HRMS of **9**

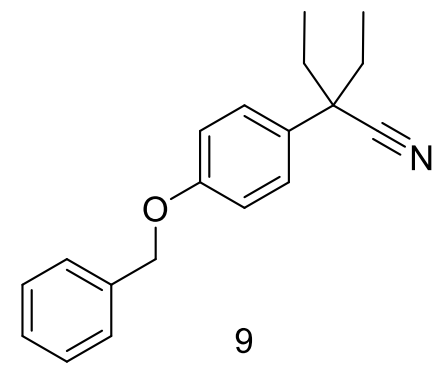

Mass Spectrum SmartFormula Report

Analysis Info

Analysis Name	D:\Data\Gleason\2022-06-29 Gleason-Caza AC-03-03 ESI +ve.d	Acquisition Date	6/29/2022 12:41:07 PM
Method	Tune_pos_Low_Na_Formate_100-1000.m	Operator	Alex
Sample Name	2022-06-29 Gleason-Caza AC-03-03 ESI +ve	Instrument	maXis impact 282001.00044
Comment			

Acquisition Parameter

Source Type	ESI	Ion Polarity	Positive	Set Nebulizer	0.6 Bar
Focus	Not active	Set Capillary	3500 V	Set Dry Heater	180 °C
Scan Begin	100 m/z	Set End Plate Offset	-500 V	Set Dry Gas	4.0 l/min
Scan End	1000 m/z	Set Charging Voltage	2000 V	Set Divert Valve	Waste
		Set Corona	0 nA	Set APCI Heater	0 °C

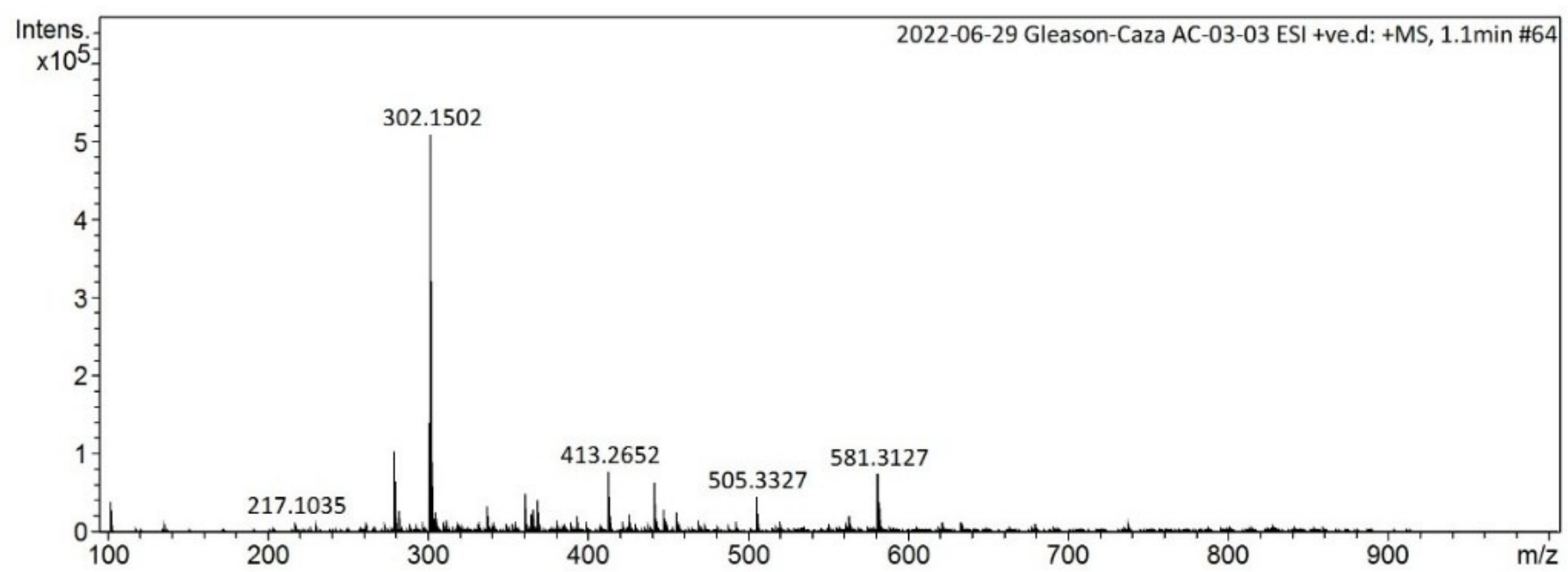

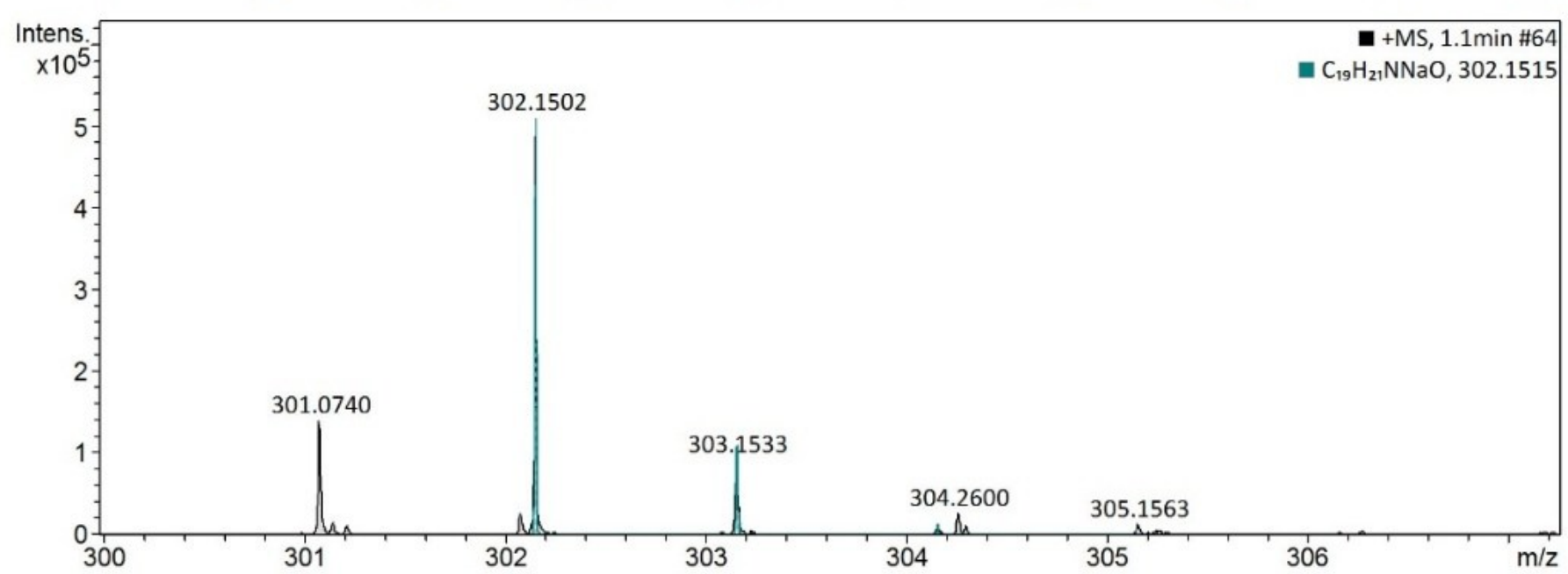

Meas. m/z	#	Ion Formula	m/z	err [ppm]	mSigma	# mSigma	Score	rdb	e⁻ Conf	N-Rule
302.1502	1	C16H20N3O3	302.1499	-0.8	6.3	1	100.00	8.5	even	ok
	1	C19H21NNaO	302.1515	4.6	20.4	1	100.00	9.5	even	ok

2022-06-29 Gleason-Caza AC-03-03 ESI +ve.d

Bruker Compass DataAnalysis 4.2 printed: 6/29/2022 6:07:18 PM by: Alex Page 1 of 1

^{1}H NMR of **10** (500 MHz, $CDCl_3$)

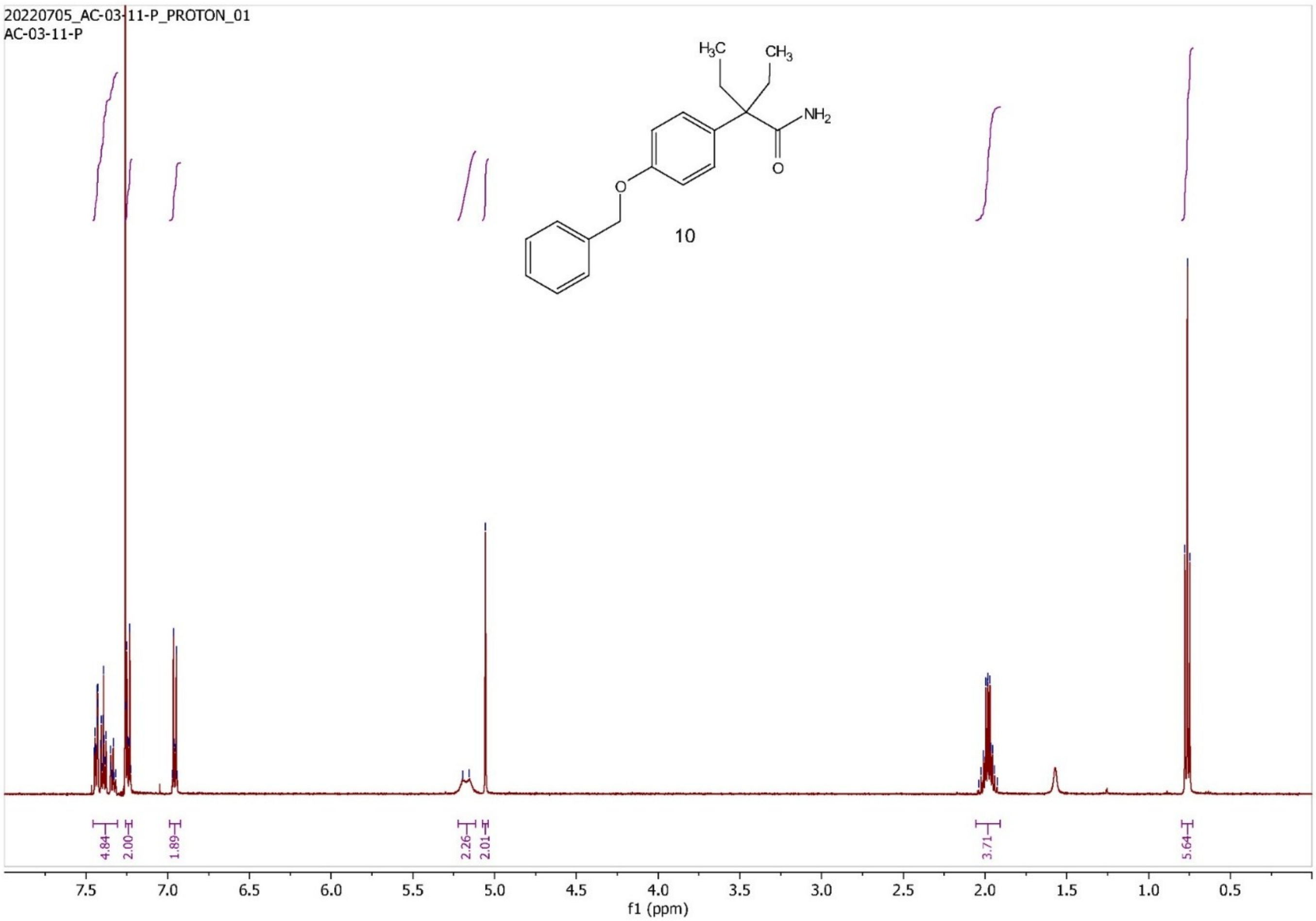

^{13}C NMR of **10** (126 MHz, $CDCl_3$)

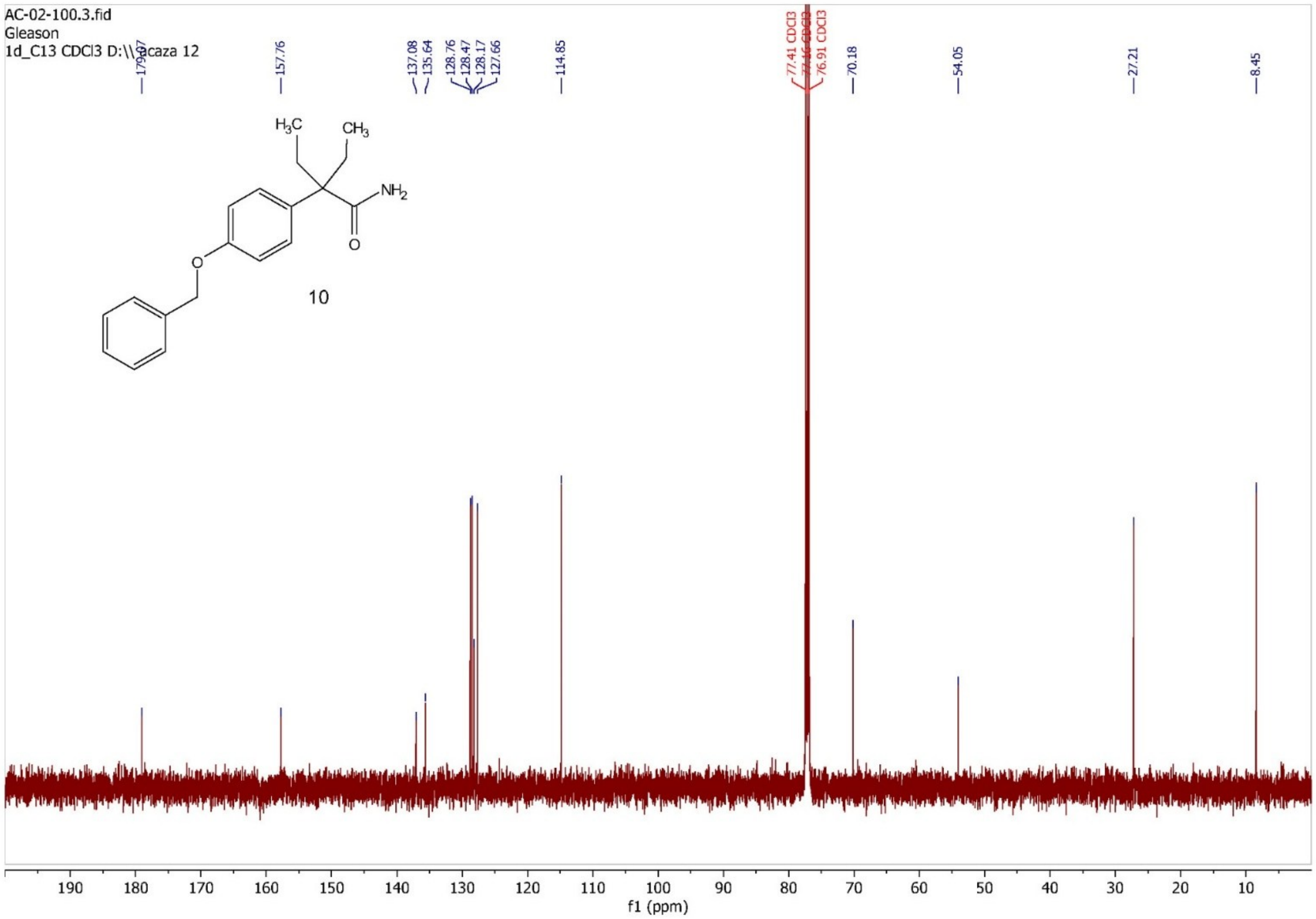

HRMS of **10**

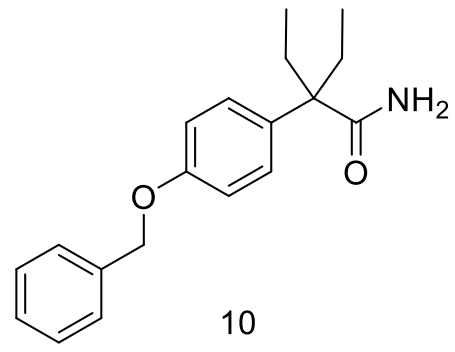

10

Mass Spectrum SmartFormula Report

Analysis Info

Analysis Name	D:\Data\Gleason\2022-06-29 Gleason-Caza AC-02-106 ESI +ve.d	Acquisition Date	6/29/2022 12:14:52 PM
Method	Tune_pos_Low_Na_Formate_100-1000.m	Operator	Alex
Sample Name	2022-06-29 Gleason-Caza AC-02-106 ESI +ve	Instrument	maXis impact 282001.00044
Comment			

Acquisition Parameter

Source Type	ESI	Ion Polarity	Positive	Set Nebulizer	0.6 Bar
Focus	Not active	Set Capillary	3500 V	Set Dry Heater	180 °C
Scan Begin	100 m/z	Set End Plate Offset	-500 V	Set Dry Gas	4.0 l/min
Scan End	1000 m/z	Set Charging Voltage	2000 V	Set Divert Valve	Waste
		Set Corona	0 nA	Set APCI Heater	0 °C

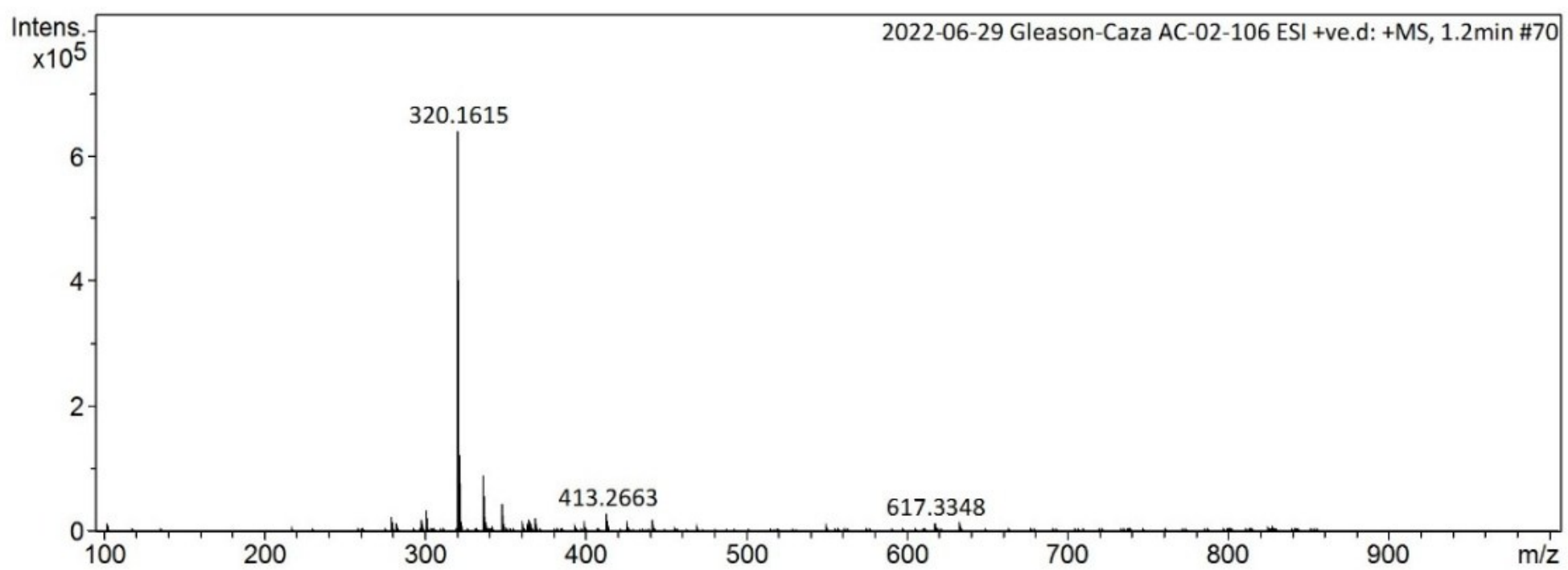

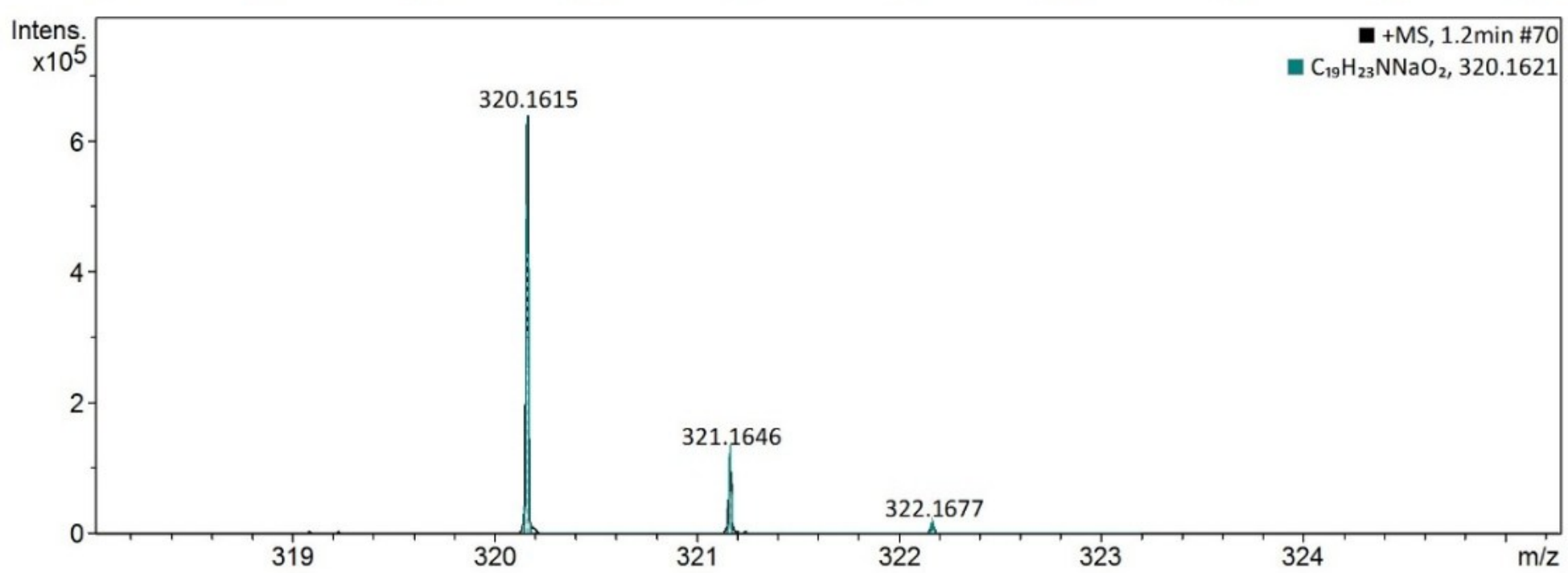

Meas. m/z	#	Ion Formula	m/z	err [ppm]	mSigma	# mSigma	Score	rdb	e⁻ Conf	N-Rule
320.1615	1	C17H18N7	320.1618	0.9	11.2	1	100.00	12.5	even	ok
	1	C19H23NNaO2	320.1621	1.8	12.1	1	93.65	8.5	even	ok
	2	C11H27N3NaO4S	320.1614	-0.2	32.5	2	100.00	-0.5	even	ok

2022-06-29 Gleason-Caza AC-02-106 ESI +ve.d

Bruker Compass DataAnalysis 4.2 printed: 6/29/2022 6:09:06 PM by: Alex Page 1 of 1

^{1}H NMR of **11** (500 MHz, $CDCl_3$)

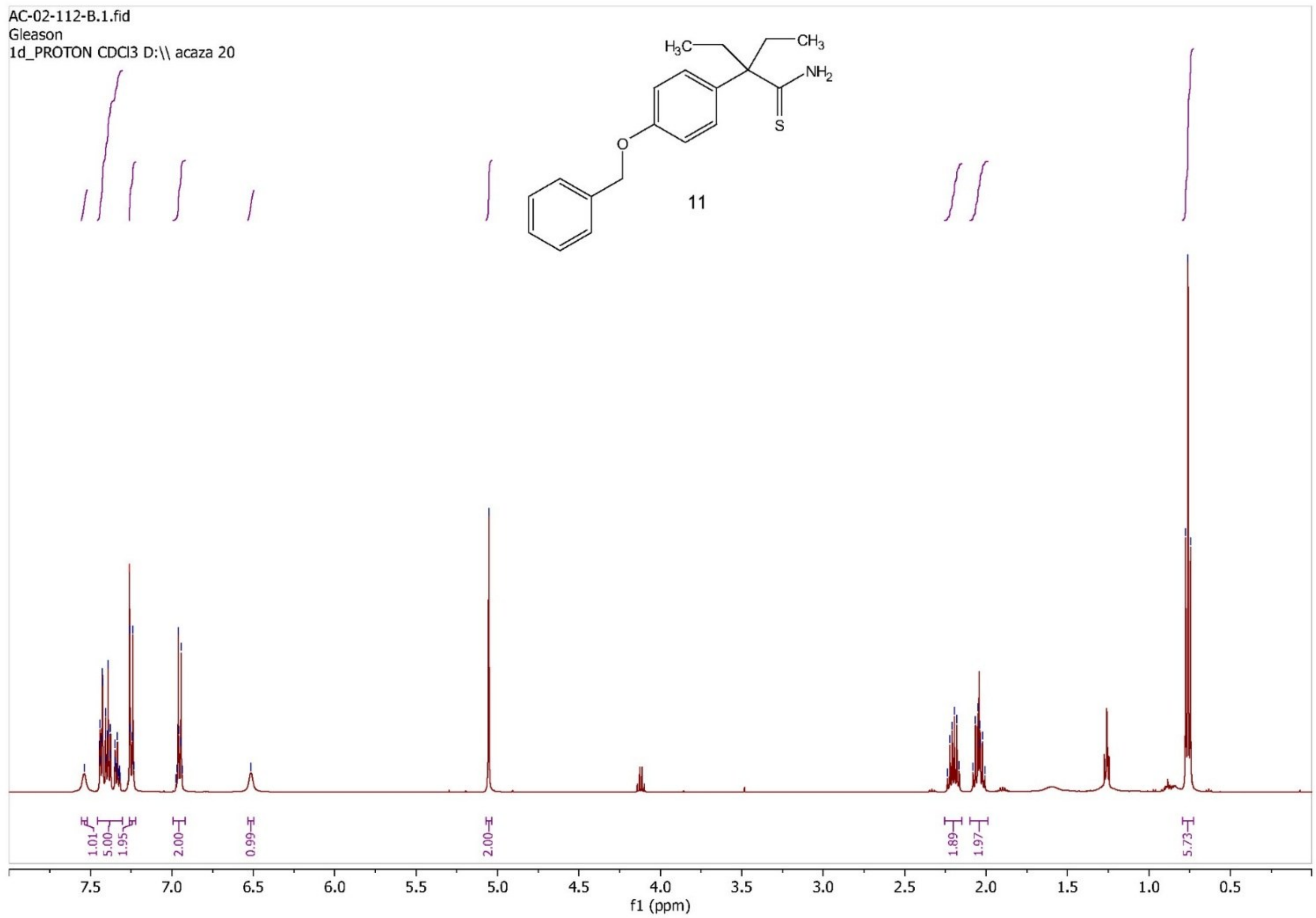

^{13}C NMR of **11** (126 MHz, $CDCl_3$)

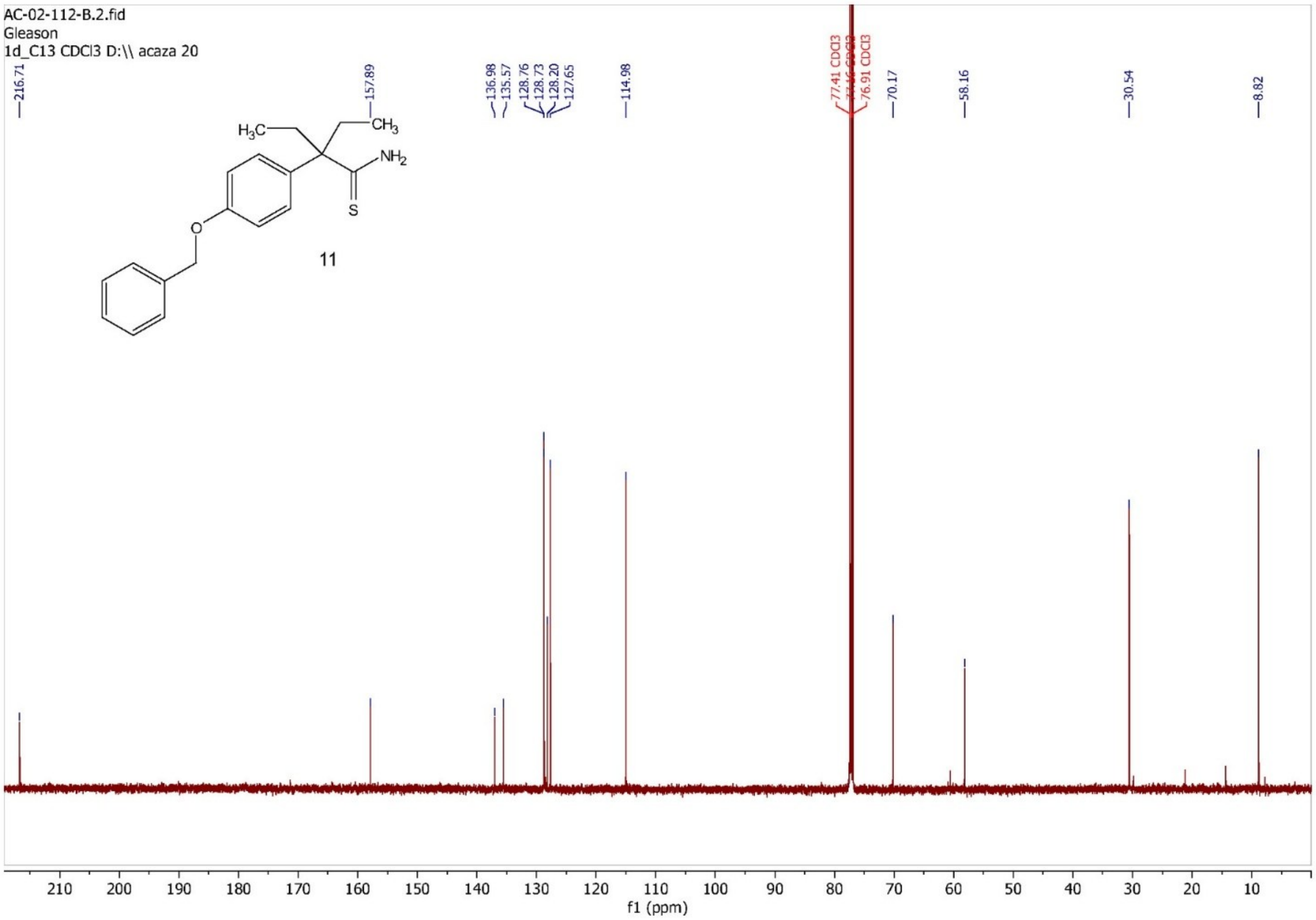

HRMS of **11**

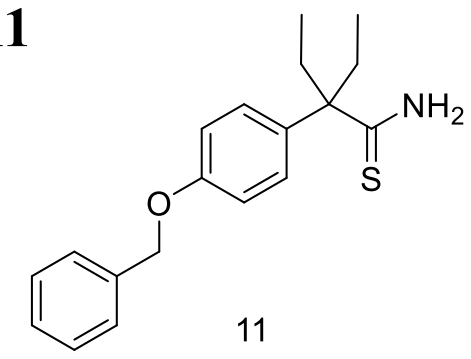

11

Mass Spectrum SmartFormula Report

Analysis Info

Analysis Name	D:\Data\Gleason\2022-06-29 Gleason-Caza AC-02-112 ESI +ve.d	Acquisition Date	6/29/2022 12:17:53 PM
Method	Tune_pos_Low_Na_Formate_100-1000.m	Operator	Alex
Sample Name	2022-06-29 Gleason-Caza AC-02-106 ESI +ve	Instrument	maXis impact 282001.00044
Comment			

Acquisition Parameter

Source Type	ESI	Ion Polarity	Positive	Set Nebulizer	0.6 Bar
Focus	Not active	Set Capillary	3500 V	Set Dry Heater	180 °C
Scan Begin	100 m/z	Set End Plate Offset	-500 V	Set Dry Gas	4.0 l/min
Scan End	1000 m/z	Set Charging Voltage	2000 V	Set Divert Valve	Waste
		Set Corona	0 nA	Set APCI Heater	0 °C

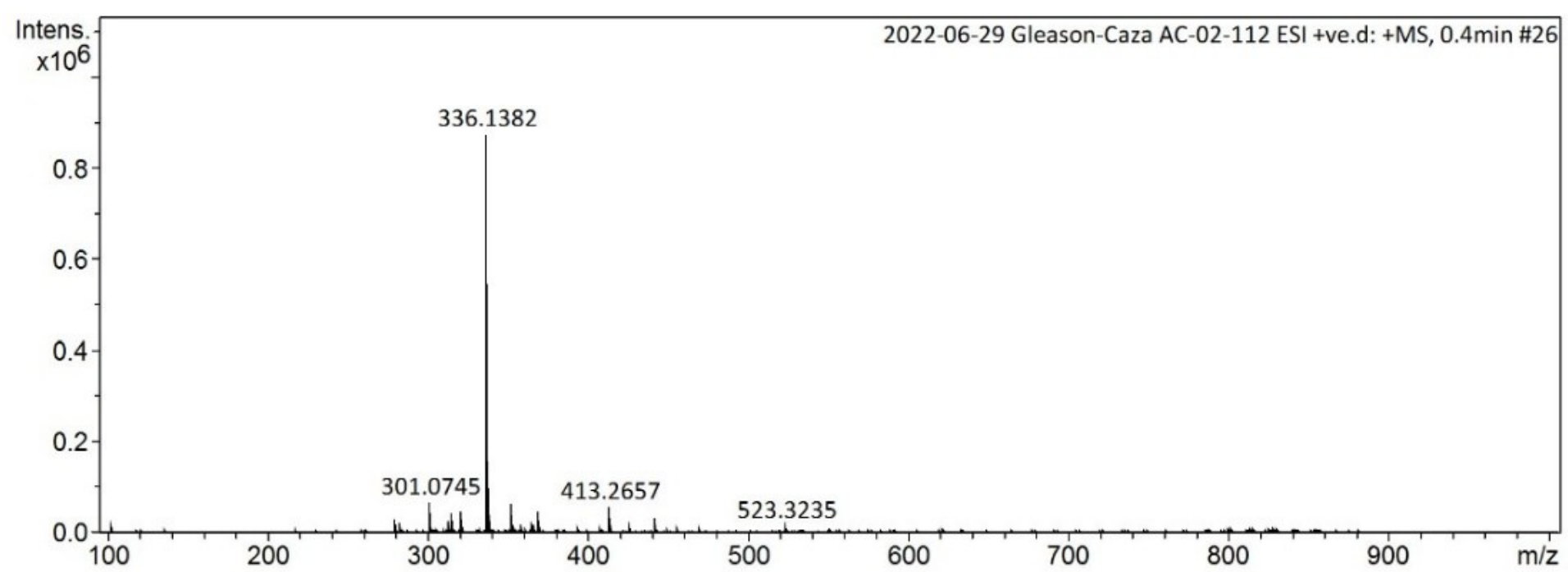

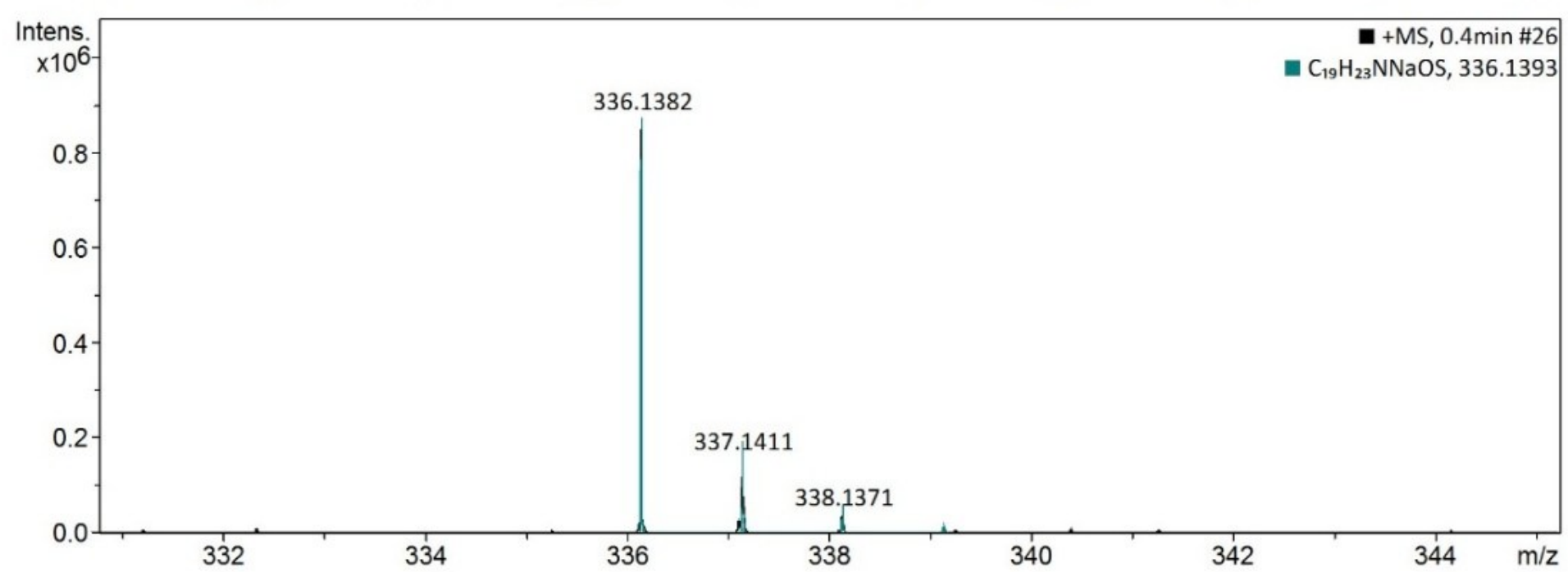

Meas. m/z	#	Ion Formula	m/z	err [ppm]	mSigma	# mSigma	Score	rdb	e⁻ Conf	N-Rule
336.1382	1	C16H22N3O3S	336.1376	-1.7	14.1	1	100.00	7.5	even	ok
	2	C24H18NO	336.1383	0.2	50.2	2	38.51	16.5	even	ok
	1	C19H23NNaOS	336.1393	3.1	23.6	1	100.00	8.5	even	ok
	2	C11H19N7NaO4	336.1391	2.5	24.0	2	82.54	5.5	even	ok
	3	C10H23N3NaO8	336.1377	-1.5	34.0	3	77.77	0.5	even	ok

2022-06-29 Gleason-Caza AC-02-112 ESI +ve.d

Bruker Compass DataAnalysis 4.2 printed: 6/29/2022 6:08:23 PM by: Alex Page 1 of 1

^{1}H NMR of **18** (500 MHz, $CDCl_3$)

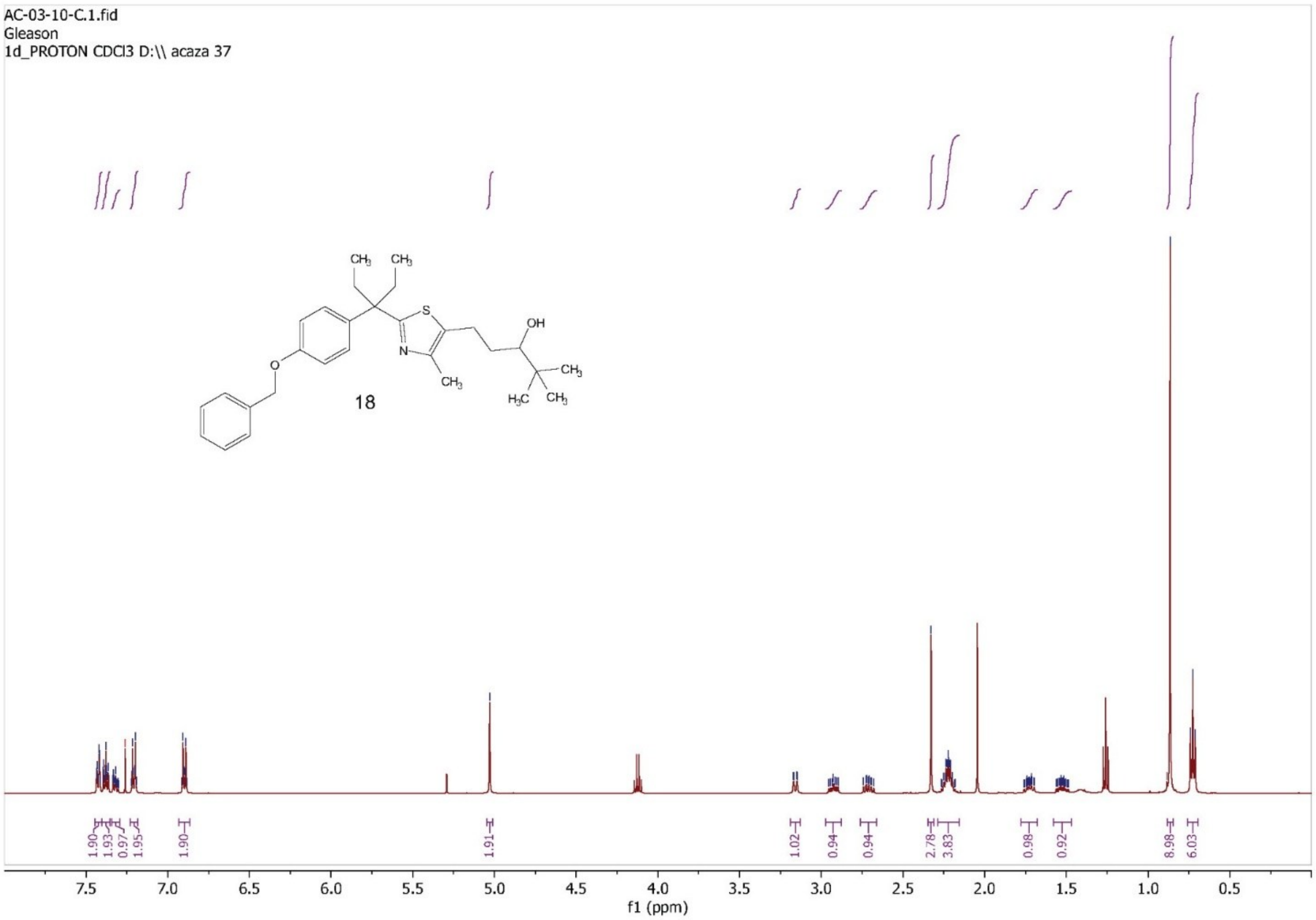

^{13}C NMR of **18** (126 MHz, $CDCl_3$)

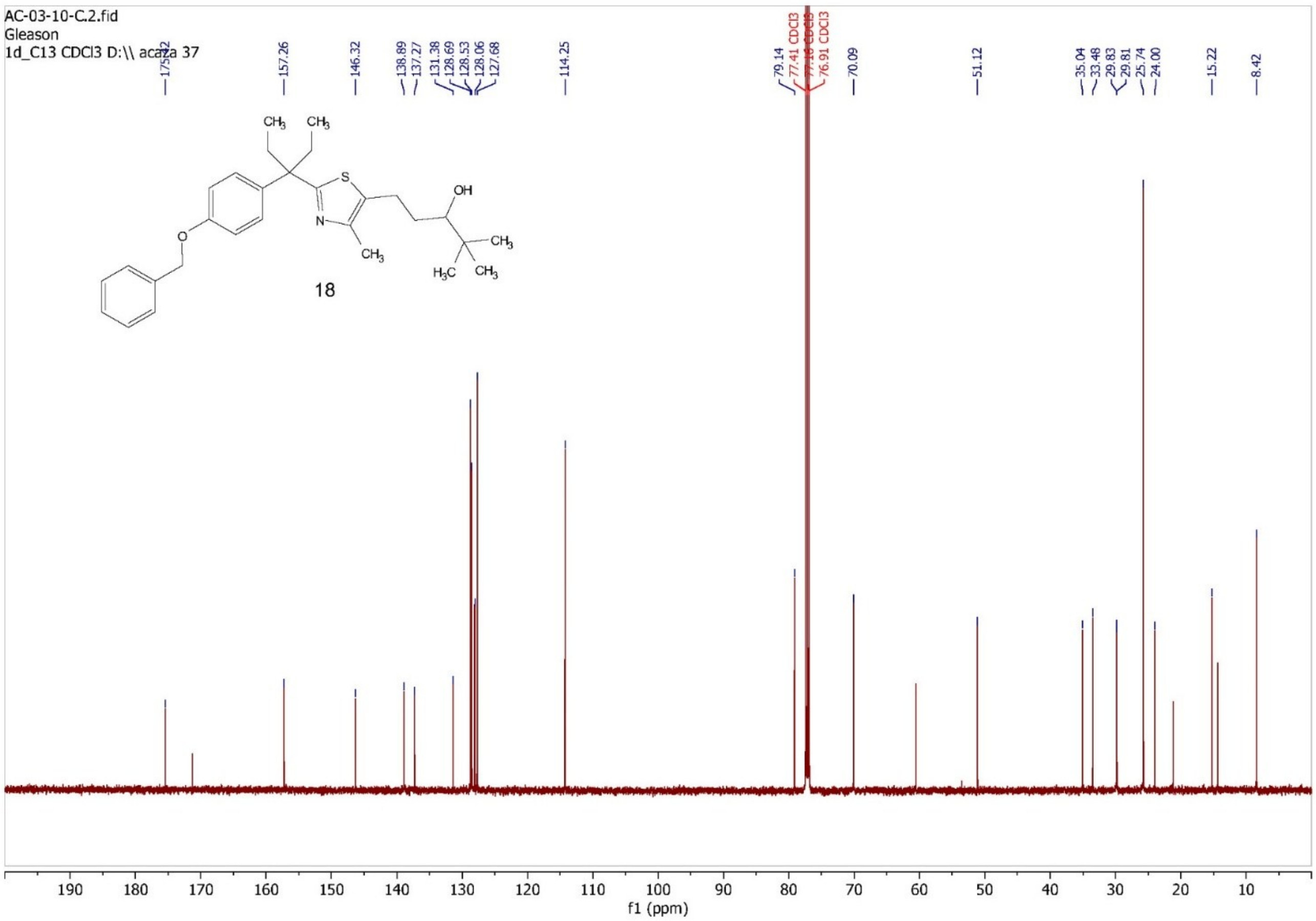

HRMS of **18**

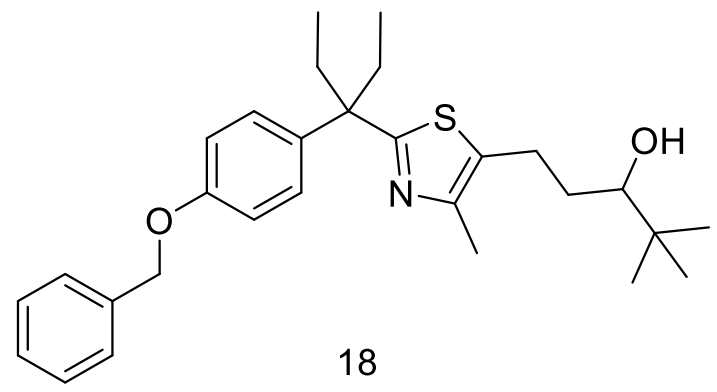

18

Mass Spectrum SmartFormula Report

Analysis Info

Analysis Name	D:\Data\Gleason\2022-06-30 Gleason-Caza AC-02-115 ESI +ve.d
Method	Tune_pos_Low_Na_Formate_100-1000.m
Sample Name	2022-06-30 Gleason-Caza AC-02-115 ESI +ve
Comment	
Acquisition Date	6/30/2022 11:36:02 AM
Operator	Alex
Instrument	maXis impact 282001.00044

Acquisition Parameter

Source Type	ESI	Ion Polarity	Positive	Set Nebulizer	0.6 Bar
Focus	Not active	Set Capillary	3500 V	Set Dry Heater	180 °C
Scan Begin	100 m/z	Set End Plate Offset	-500 V	Set Dry Gas	4.0 l/min
Scan End	1000 m/z	Set Charging Voltage	2000 V	Set Divert Valve	Waste
		Set Corona	0 nA	Set APCI Heater	0 °C

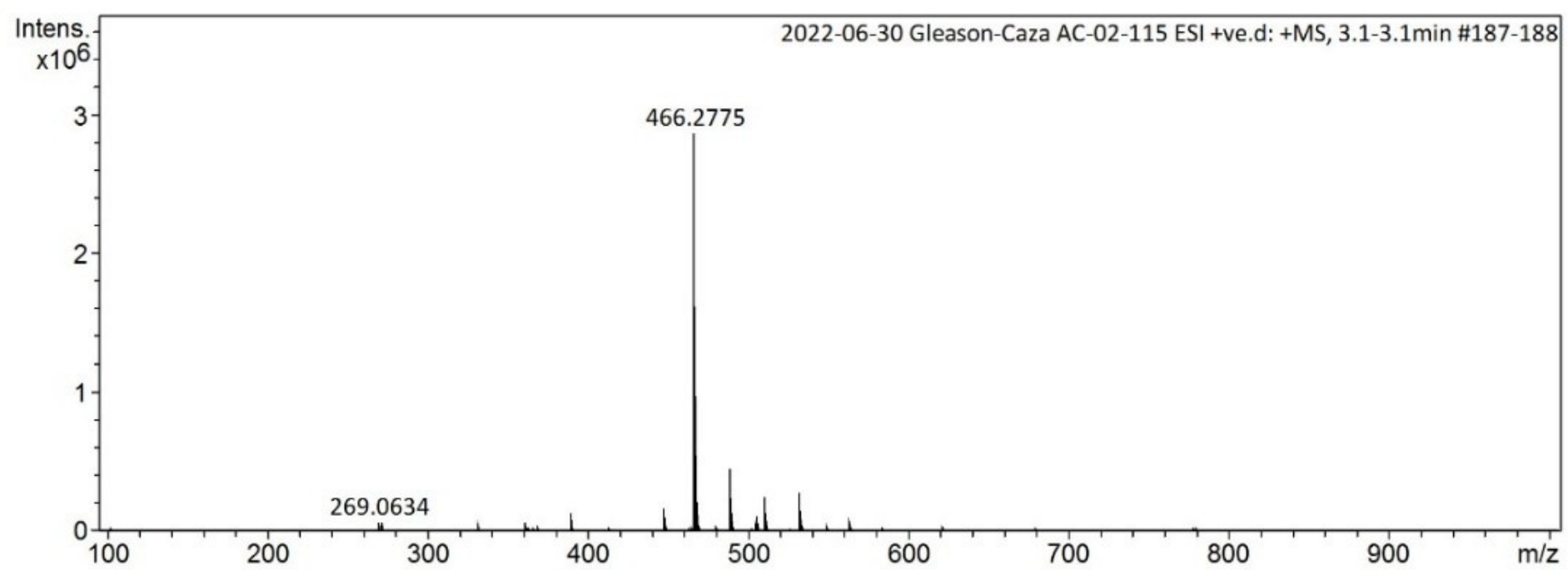

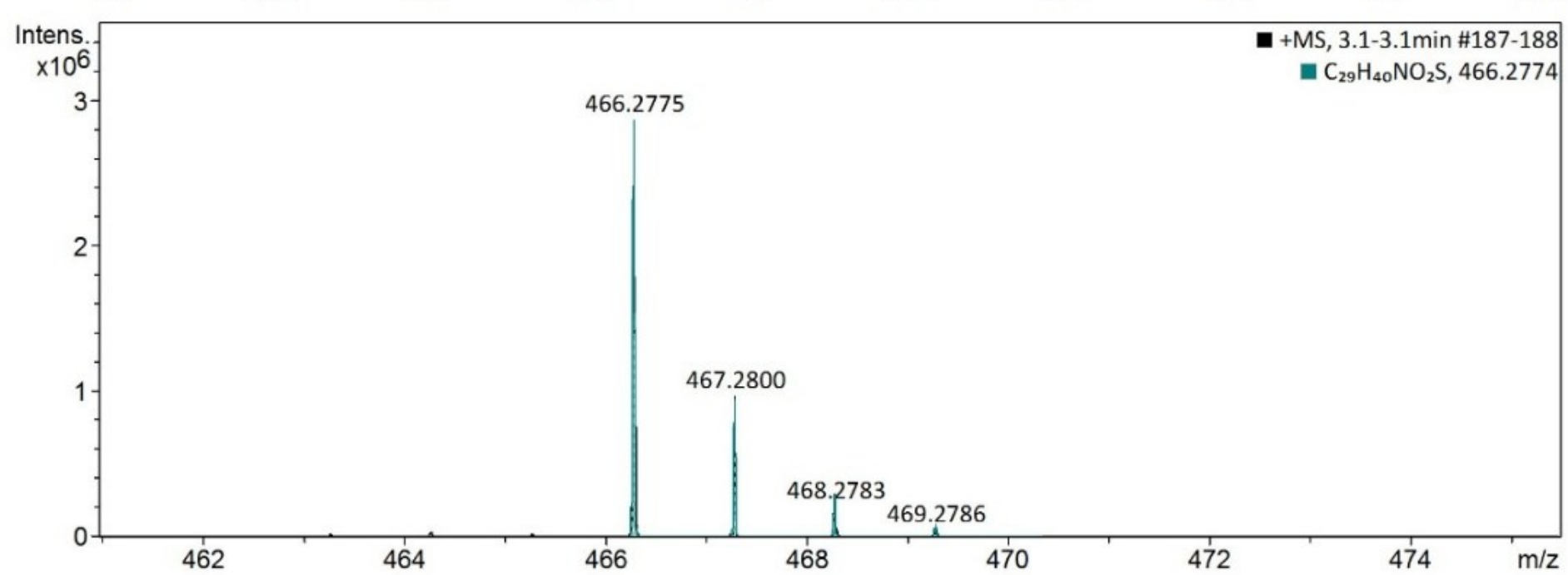

Meas. m/z	#	Ion Formula	m/z	err [ppm]	mSigma	# mSigma	Score	rdb	e⁻ Conf	N-Rule
466.2775	1	C29H40NO2S	466.2774	-0.2	15.3	1	100.00	10.5	even	ok

^{1}H NMR of **19** (500 MHz, $CDCl_3$)

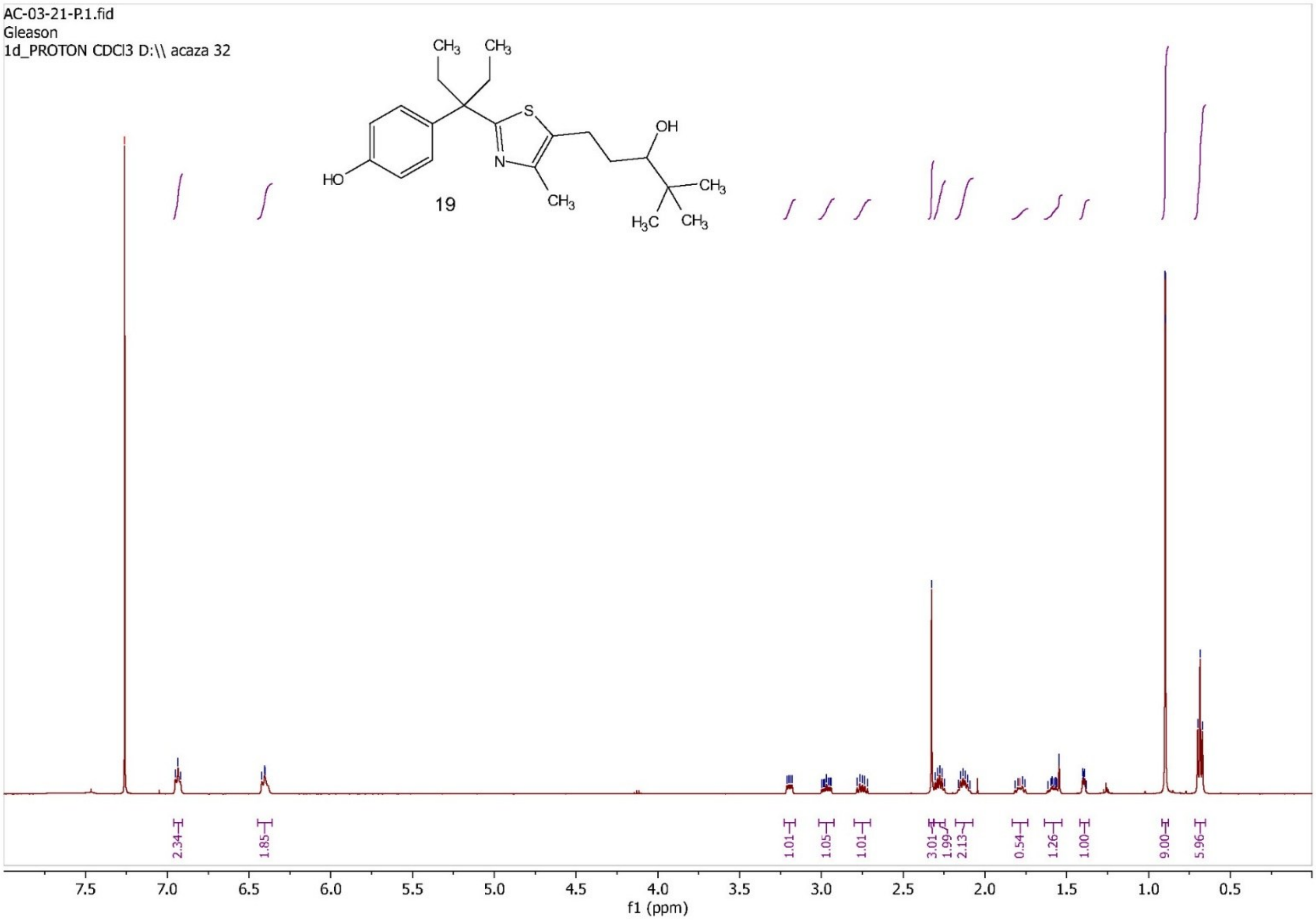

^{13}C NMR of **19** (126 MHz, $CDCl_3$)

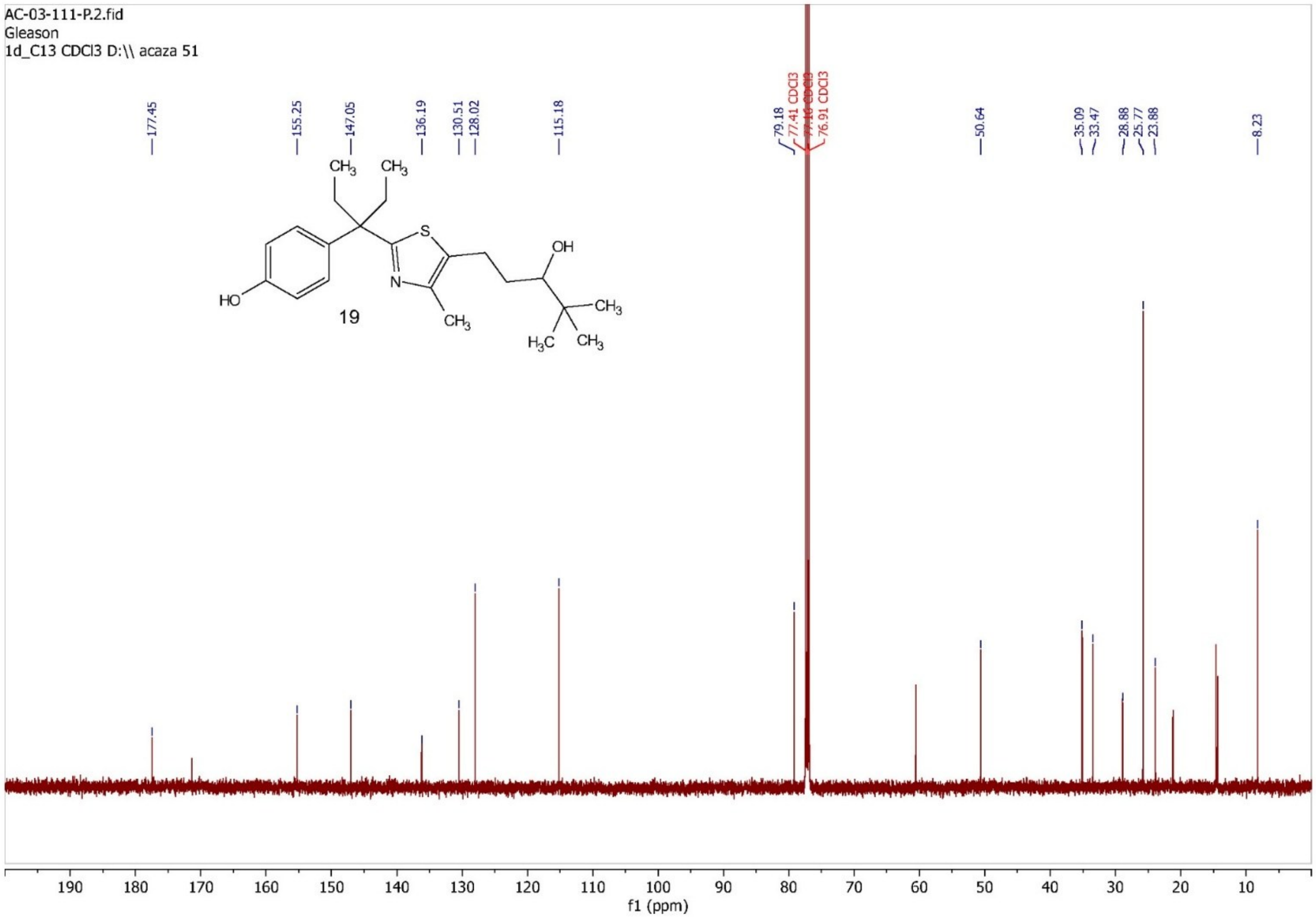

HRMS of **19**

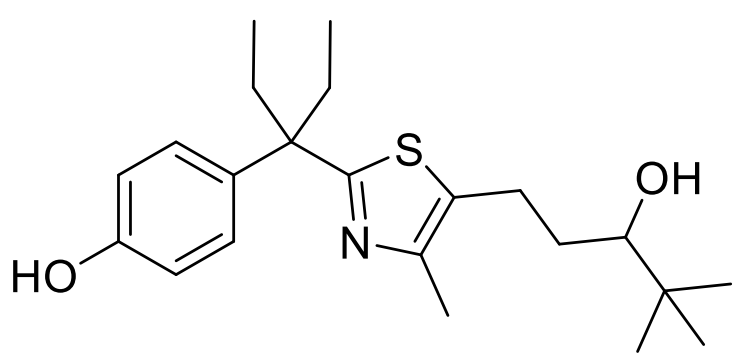

19

220913-04ESIinfhf- HRMS-Glea 09/13/22 12:02:43

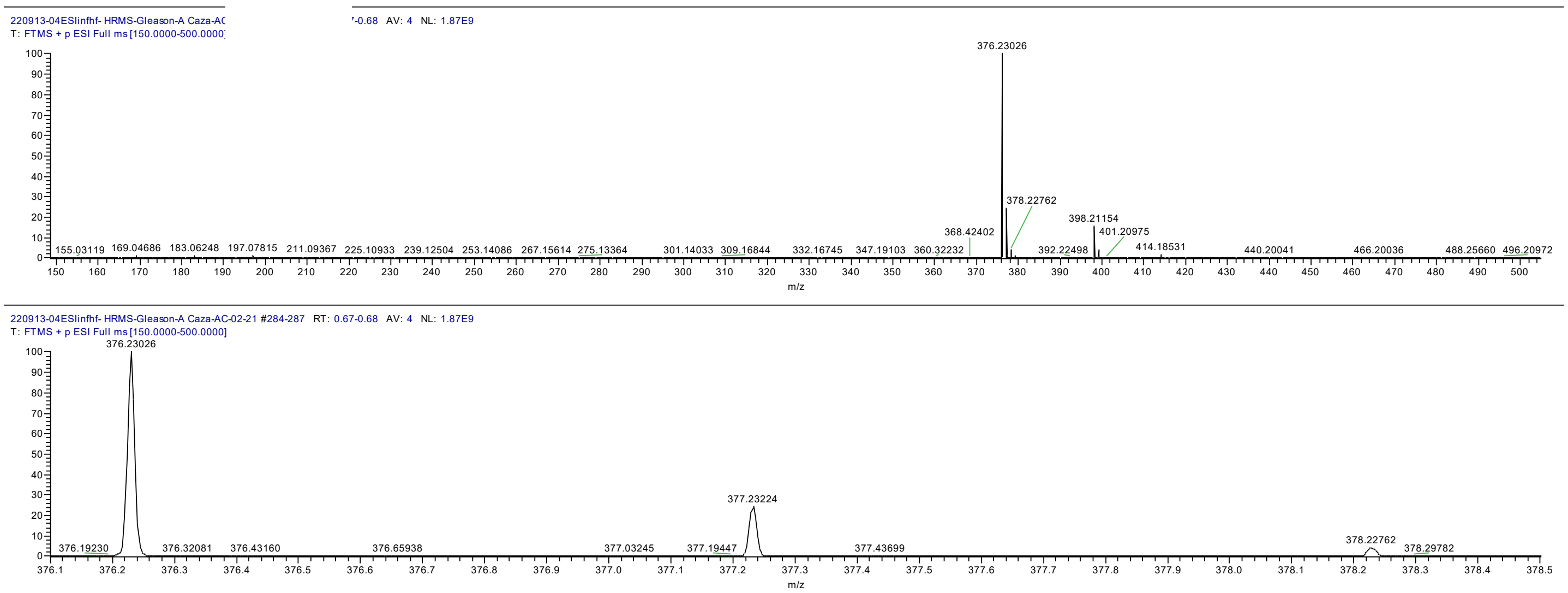

220913-04ESIinfhf- HRMS-Gleason-A Caza-AC-02-21#284-287 RT: 0.67-0.68 AV: 4
T: FTMS + p ESI Full ms [150.0000-500.0000]
m/z= 376.21084-376.24739

m/z	Intensity	Relative	Resolution	Charge	Theo. Mass	Delta (ppm)	RDB equiv.	Composition
376.23026	1875428736.0	100.00	28400.75	1.00	376.23048	-0.58	6.5	$C_{22}H_{34}O_2NS$

^{1}H NMR of **20a** (500 MHz, $CDCl_3$)

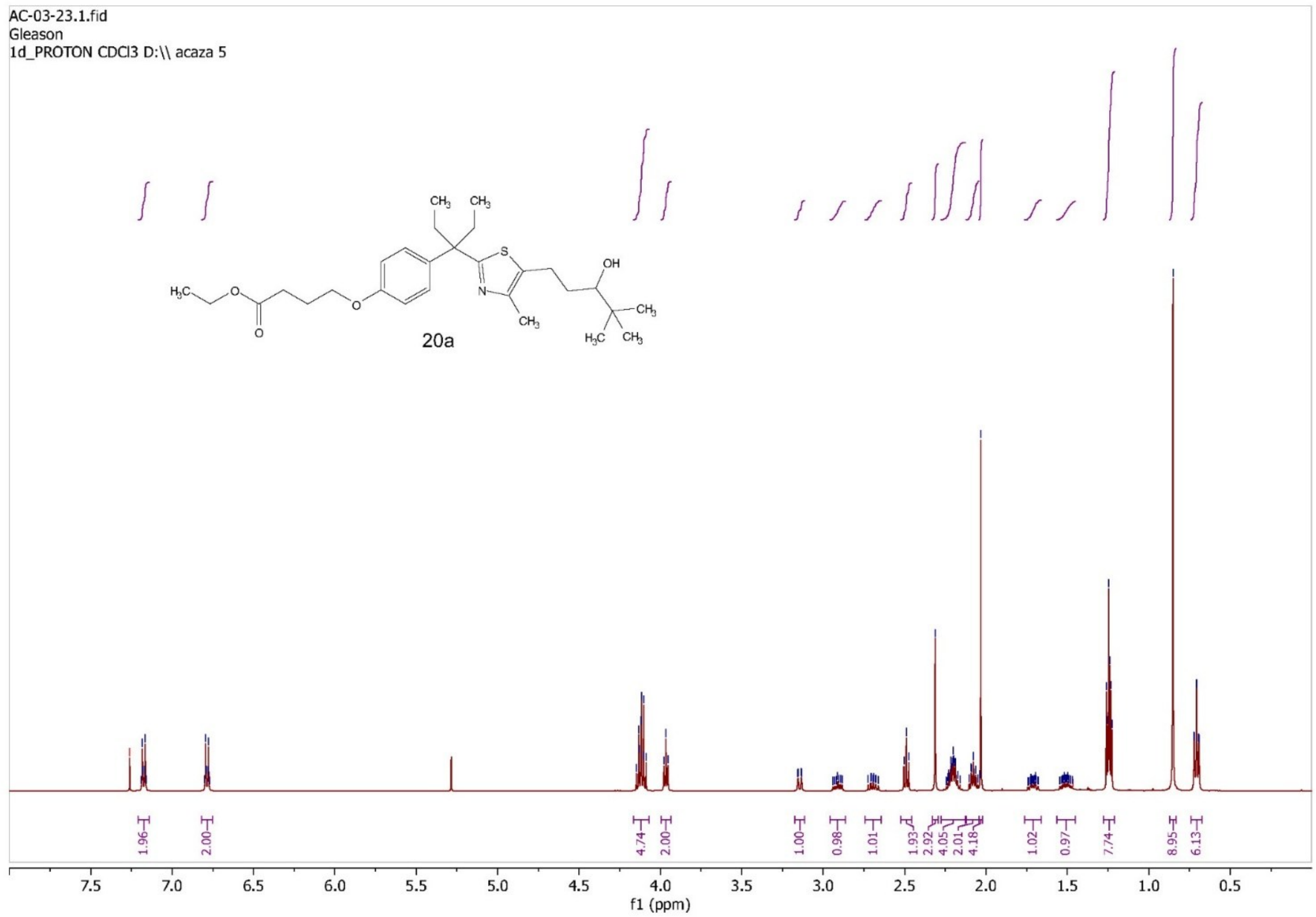

^{13}C NMR of **20a** (126 MHz, $CDCl_3$)

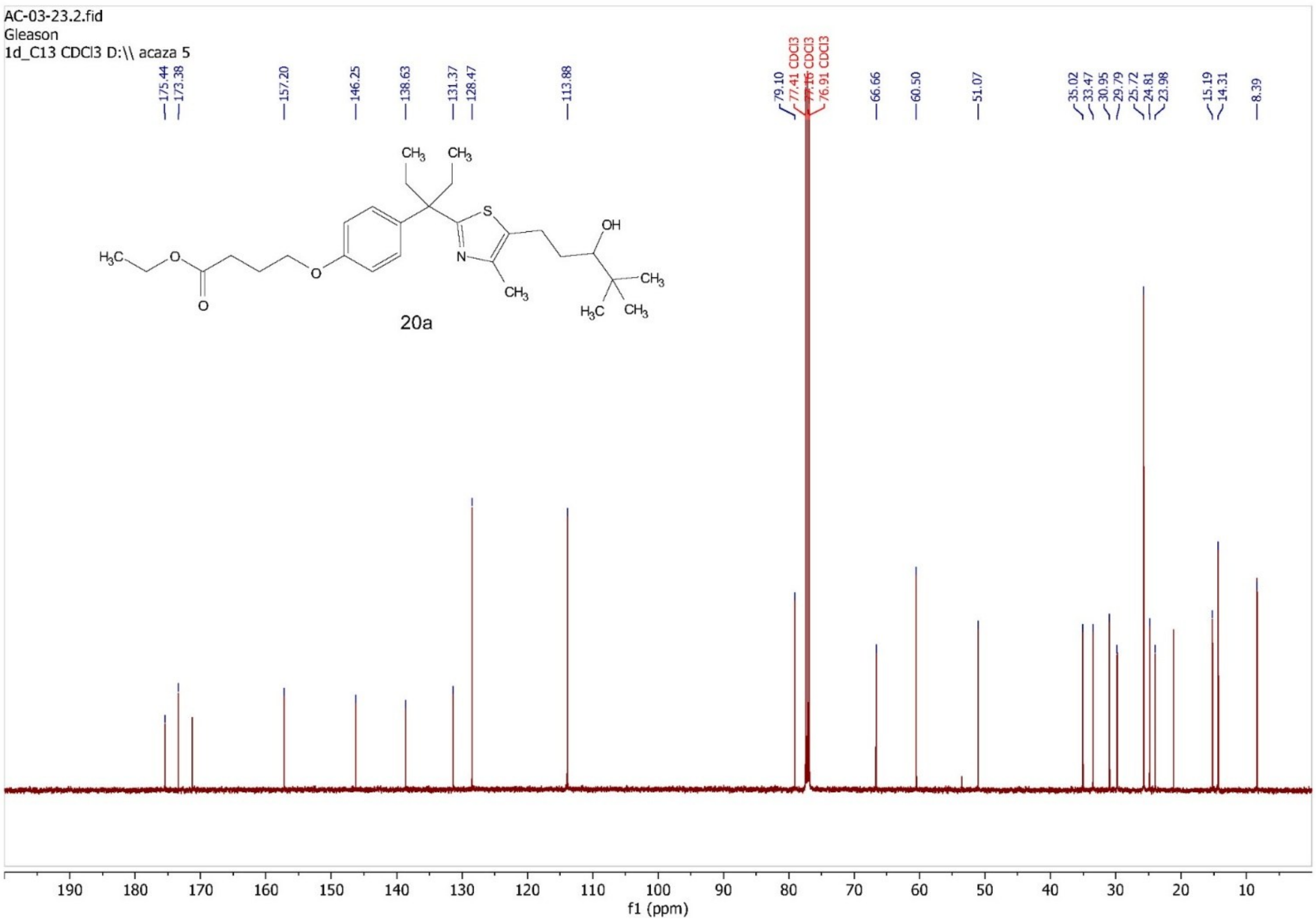

HRMS of **20a**

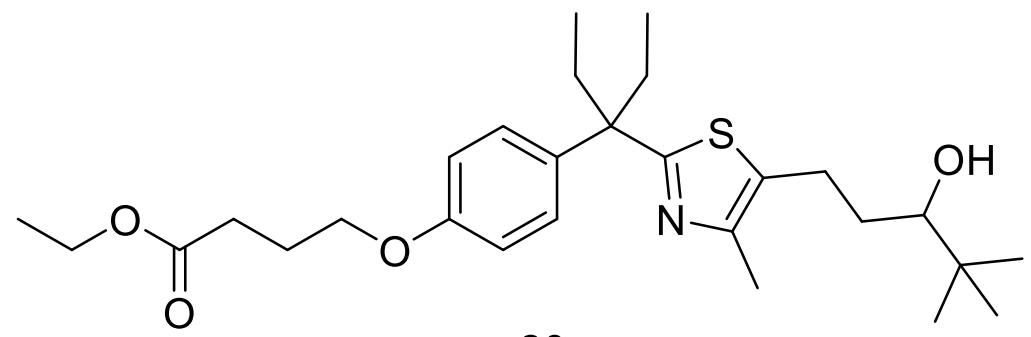

20a

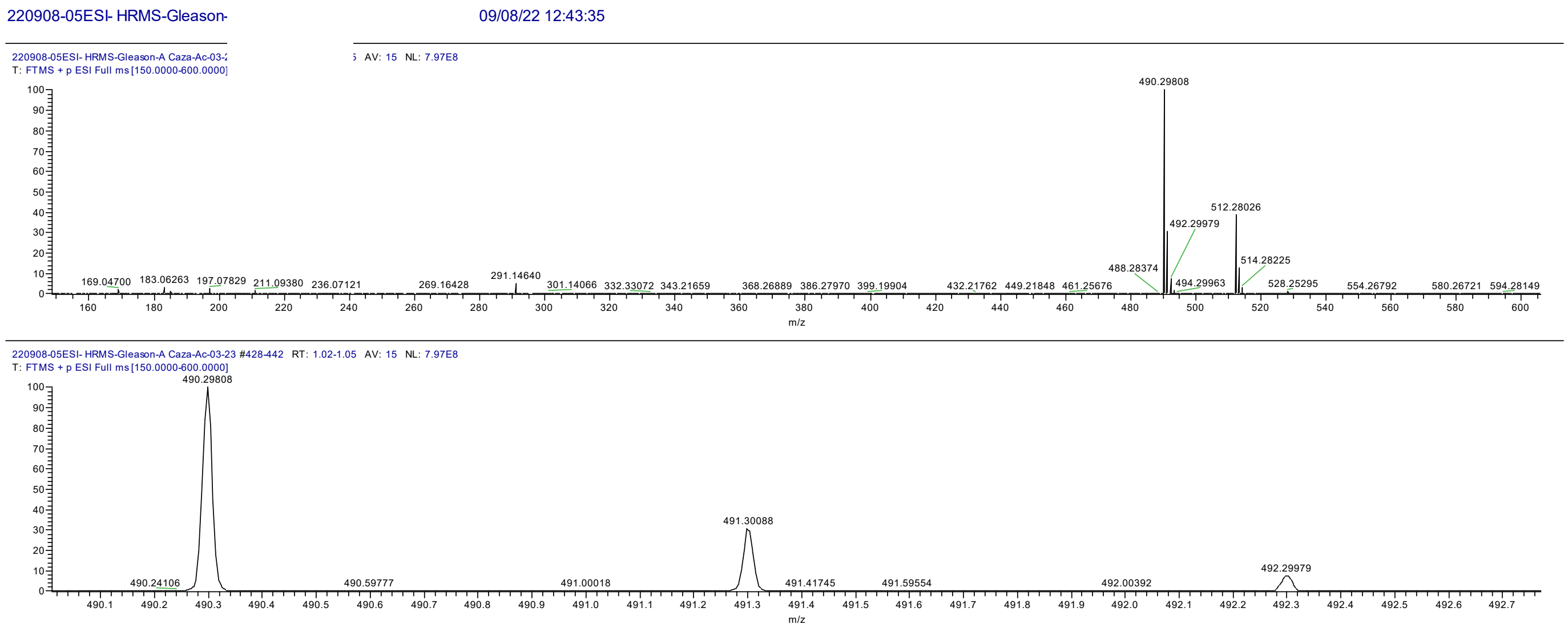

220908-05ESI- HRMS-Gleason-A Caza-Ac-03-23#428-442 RT: 1.02-1.05 AV: 15
T: FTMS + p ESI Full ms [150.0000-600.0000]
m/z= 490.27235-490.31437

m/z	Intensity	Relative	Resolution	Charge	Theo. Mass	Delta (ppm)	RDB equiv.	Composition
490.29808	796927808.0	100.00	24460.82	1.00	490.29856	-0.97	7.5	$C_{28} H_{44} O_4 N S$

^{1}H NMR of **20b** (500 MHz, $CDCl_3$)

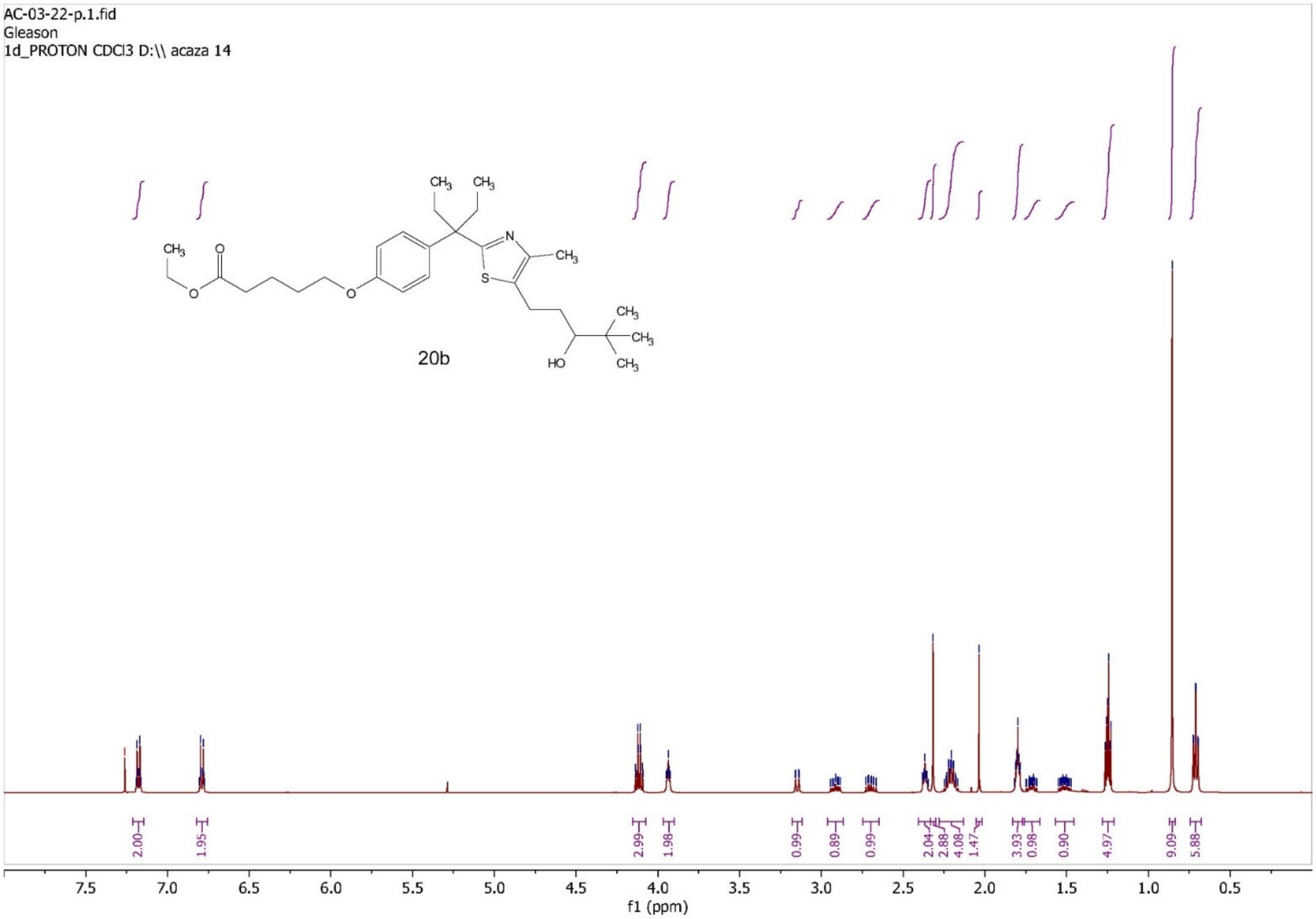

^{13}C NMR of **20b** (126 MHz, $CDCl_3$)

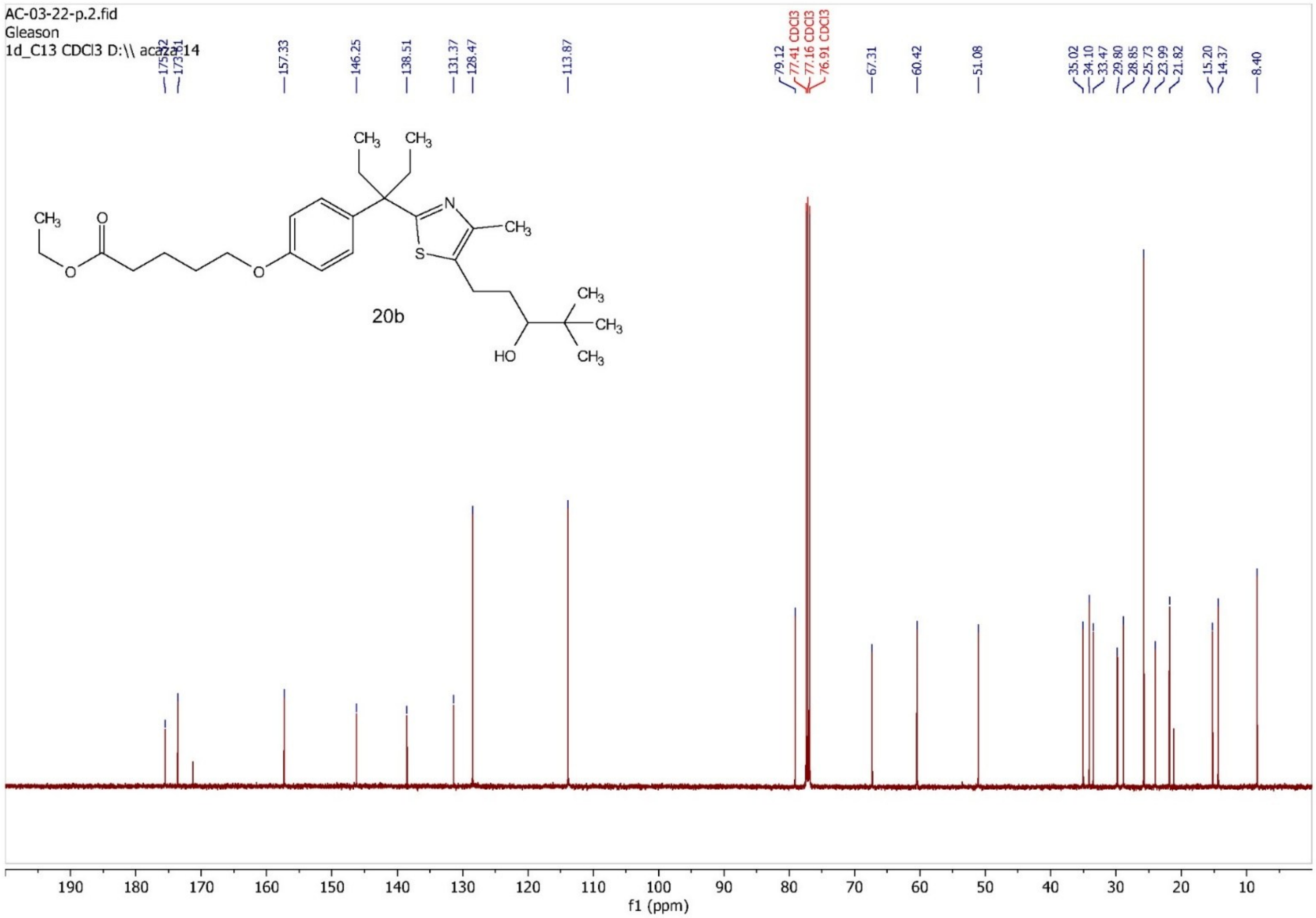

HRMS of **20b**

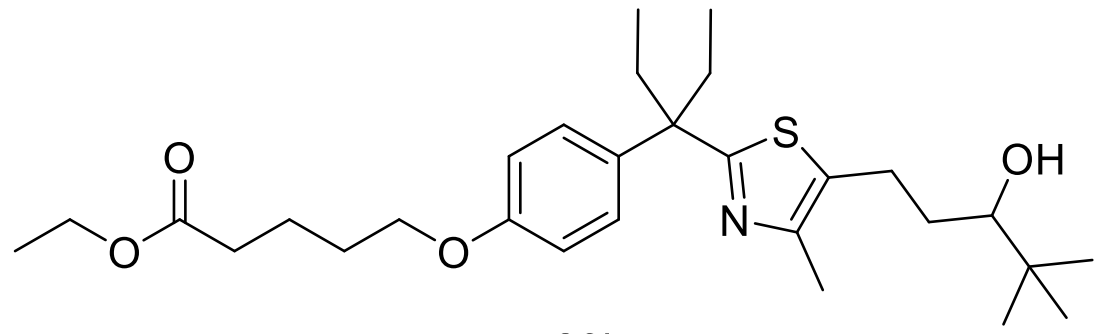

20b

220908-07ESI- HRMS-Gleason-   09/08/22 12:55:12

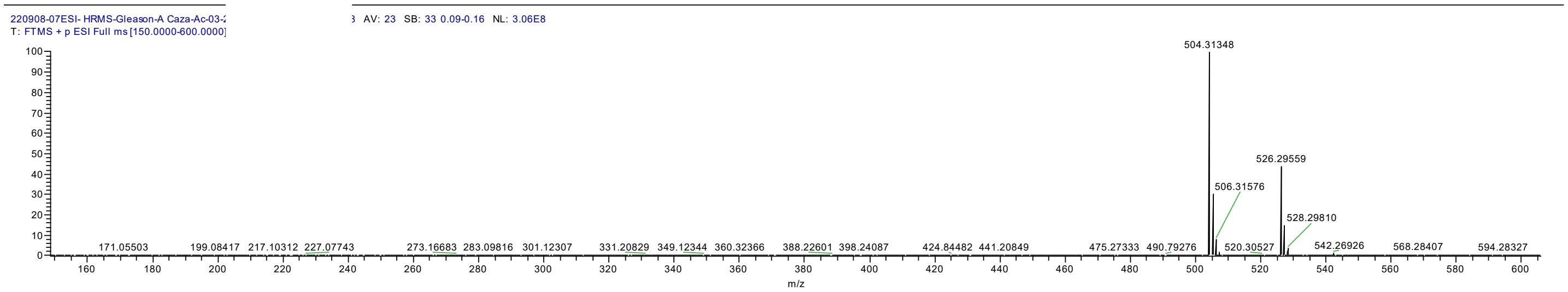

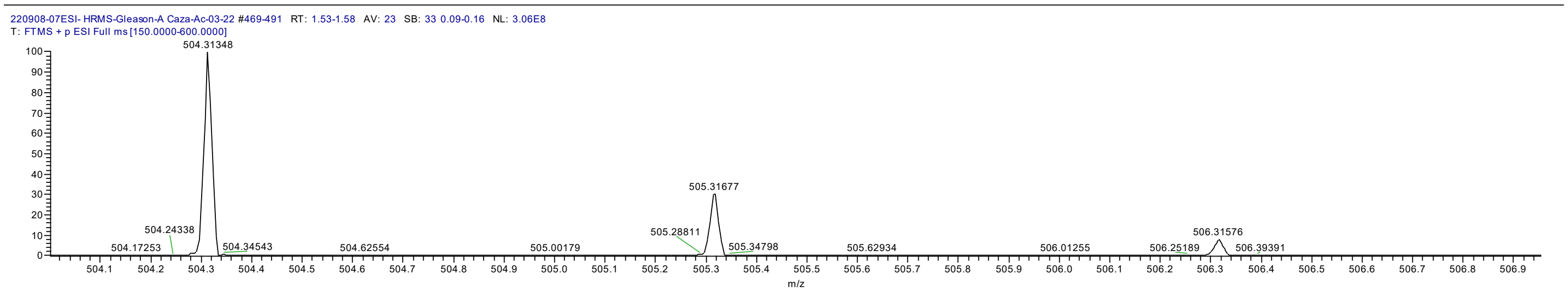

220908-07ESI- HRMS-Gleason-A Caza-Ac-03-22#469-491 RT: 1.53-1.58 AV: 23
SB: 33 0.09-0.16
T: FTMS + p ESI Full ms [150.0000-600.0000]
m/z= 504.28587-504.32885

m/z	Intensity	Relative	Resolution	Charge	Theo. Mass	Delta (ppm)	RDB equiv.	Composition
504.31348	306233440.0	100.00	28354.57	1.00	504.31421	-1.43	7.5	$C_{29}H_{46}O_4NS$

^{1}H NMR of **20c** (500 MHz, $CDCl_3$)

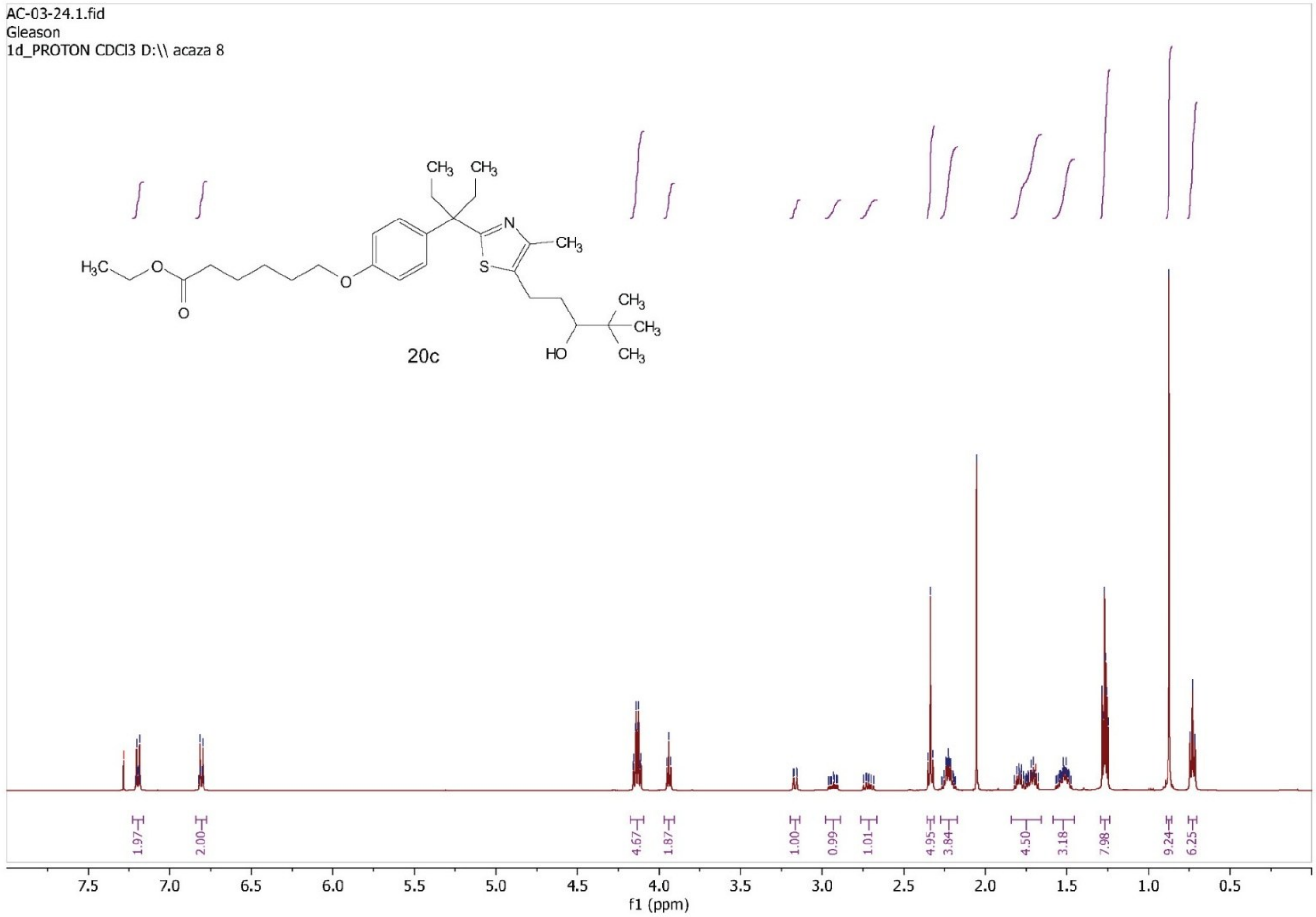

^{13}C NMR of **20c** (126 MHz, $CDCl_3$)

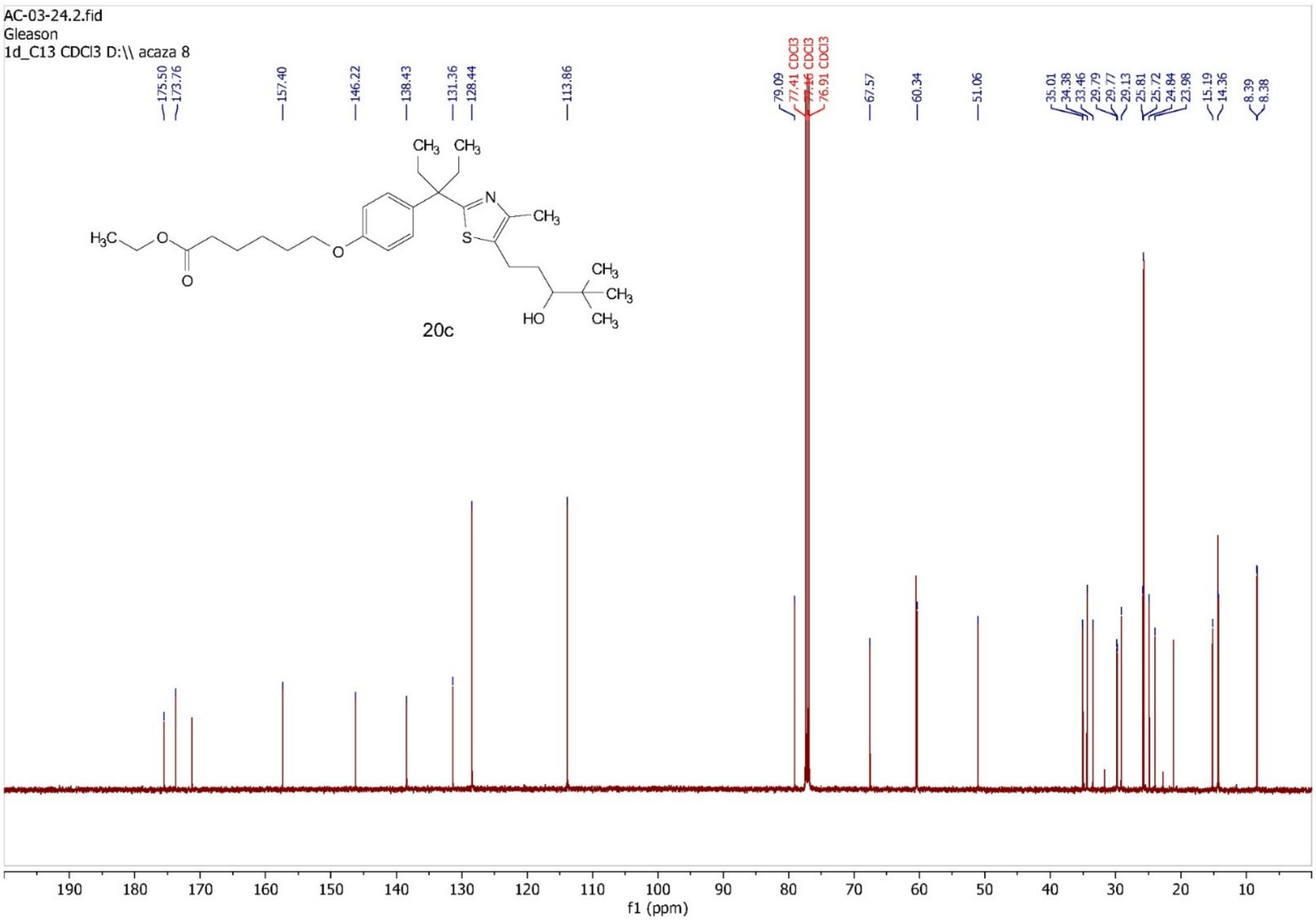

HRMS of **20c**

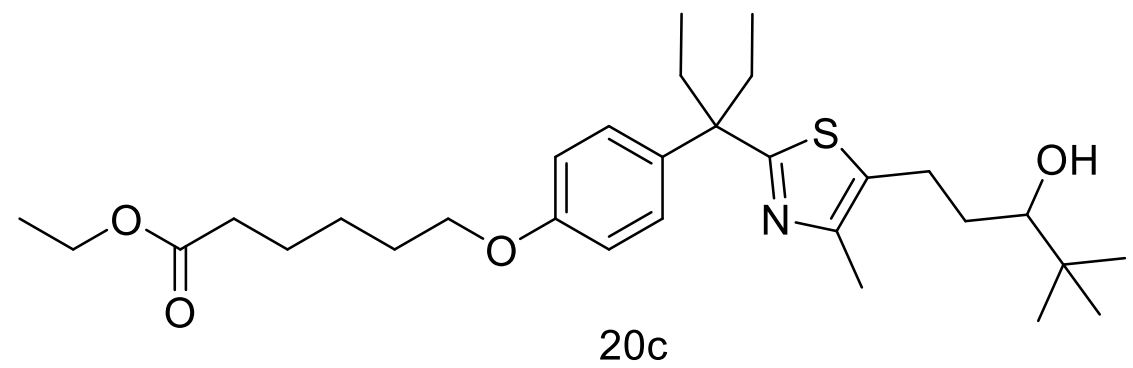

20c

220908-06ESI- HRMS-Gleason- 09/08/22 12:49:06

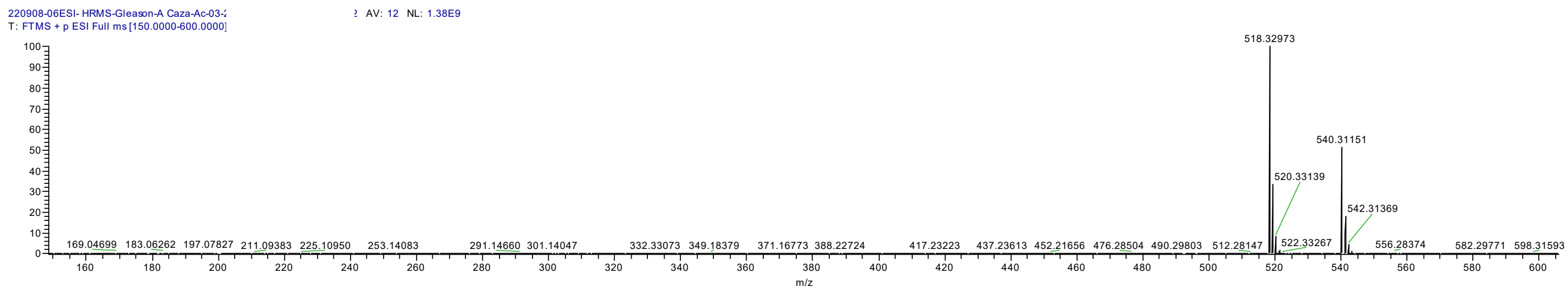

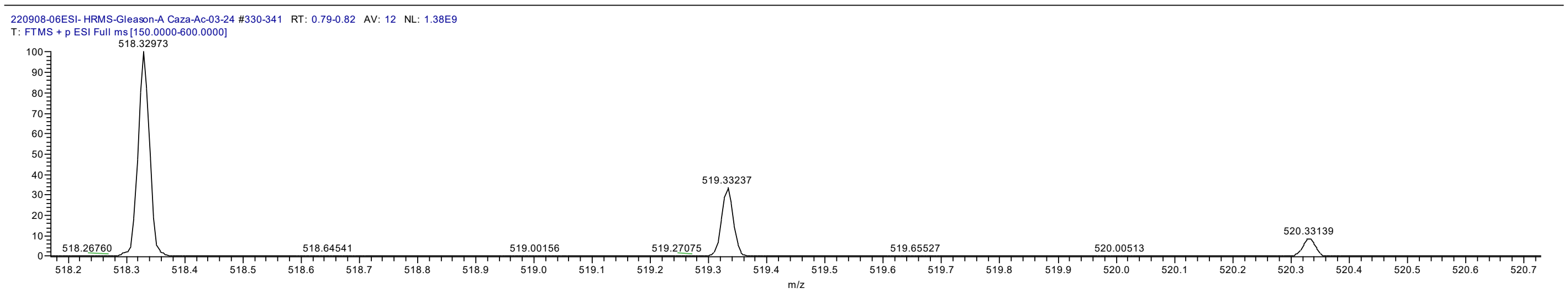

220908-06ESI- HRMS-Gleason-A Caza-Ac-03-24#330-341 RT: 0.79-0.82 AV: 12
T: FTMS + p ESI Full ms [150.0000-600.0000]
m/z= 518.30145-518.35394

m/z	Intensity	Relative	Resolution	Charge	Theo. Mass	Delta (ppm)	RDB equiv.	Composition
518.32973	1379234432.0	100.00	24043.25	1.00	518.32986	-0.24	7.5	$C_{30}H_{48}O_4NS$

^{1}H NMR of **7a** (500 MHz, DMSO-d6)

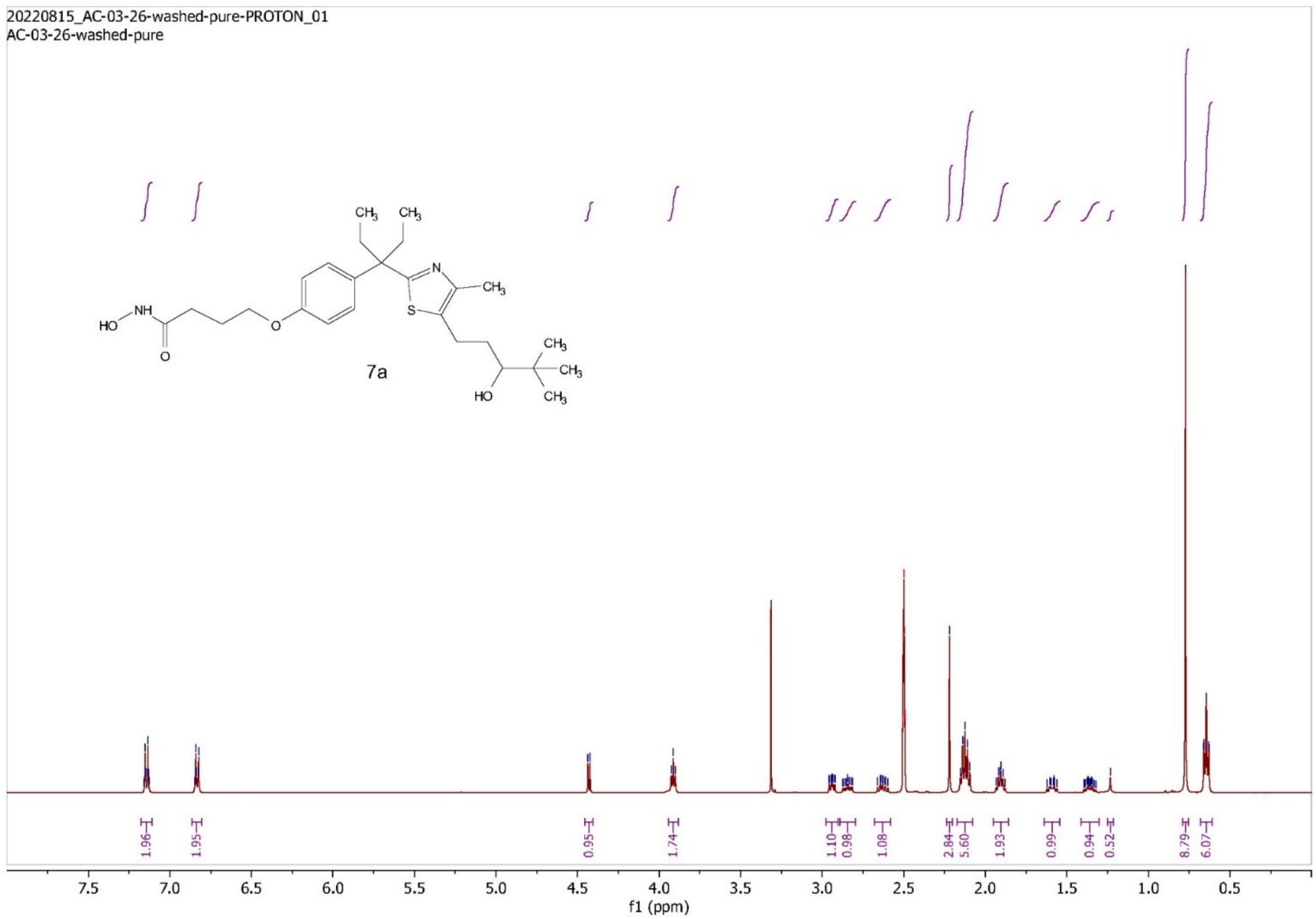

^{13}C NMR of **7a** (126 MHz, DMSO-d6)

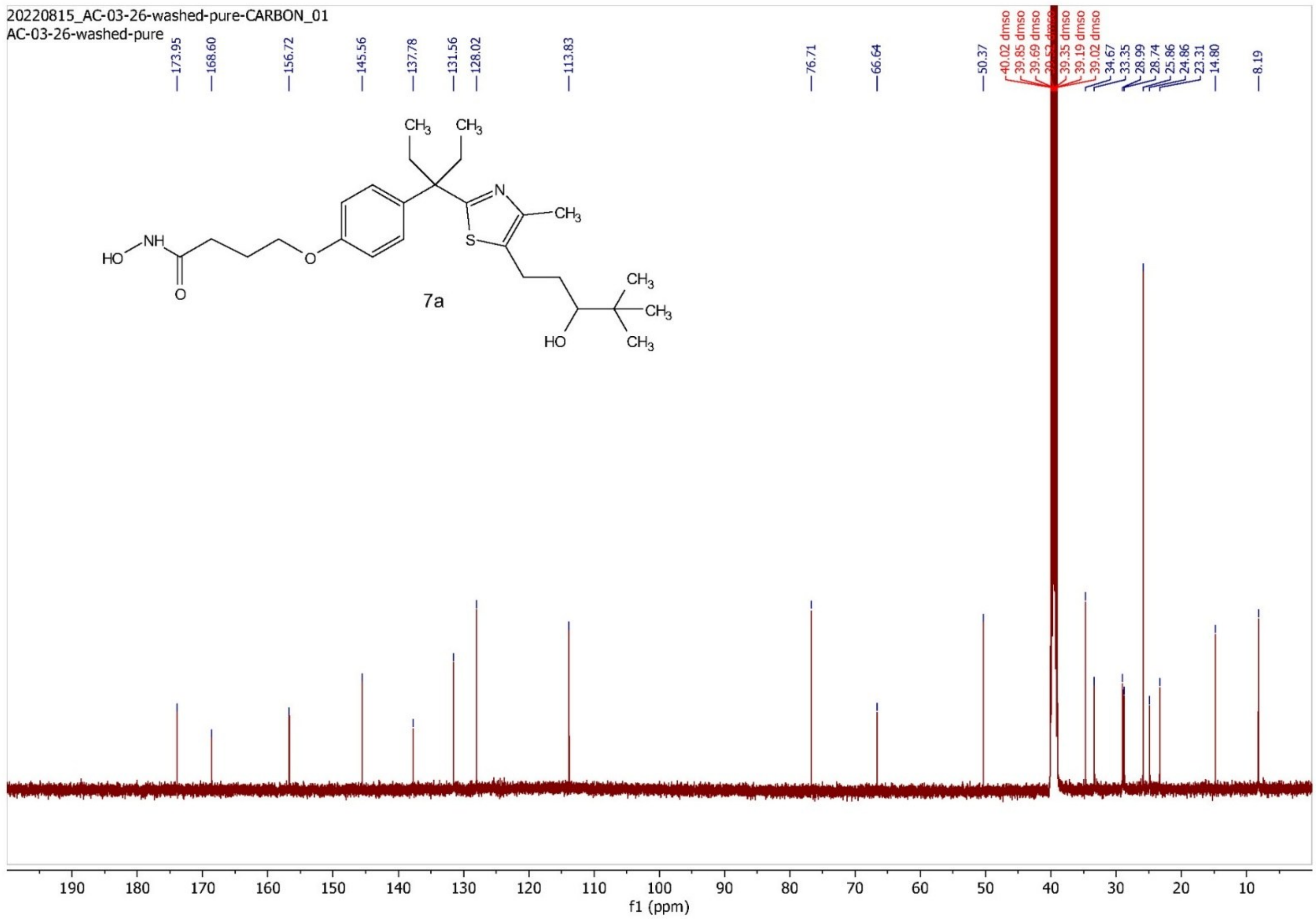

HRMS of **7a**

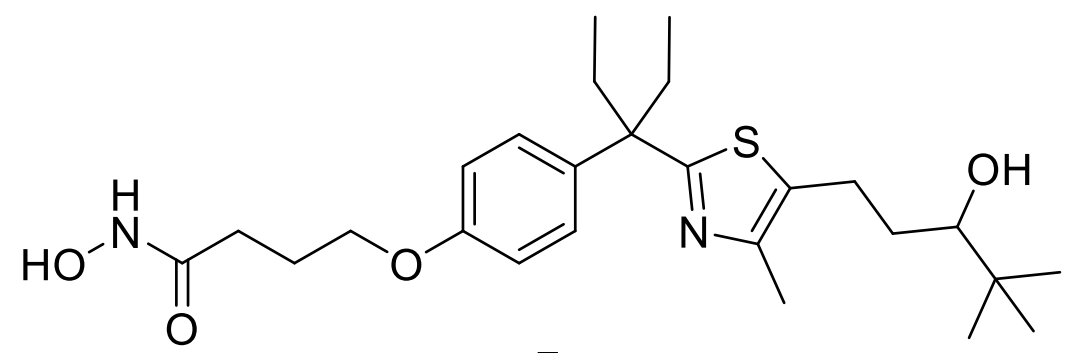

7a

22080-01ESI- HRMS-Gleason-A 08/02/22 07:38:02

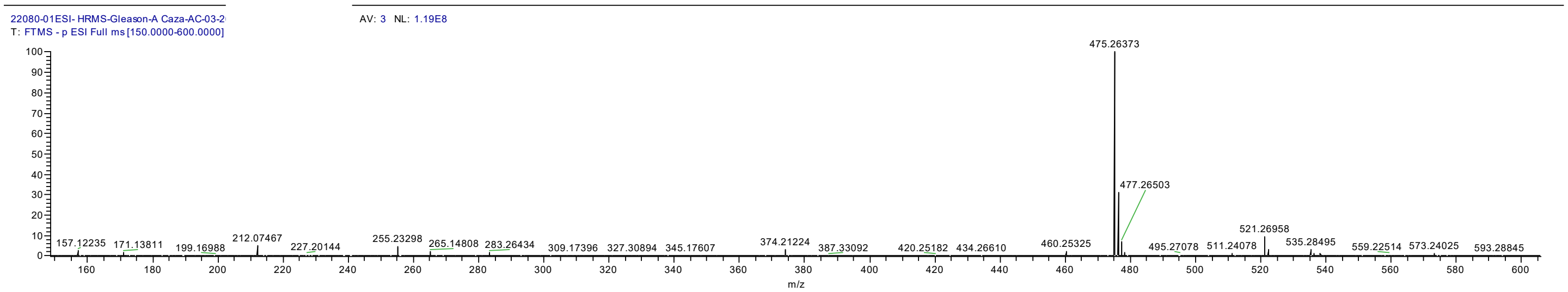

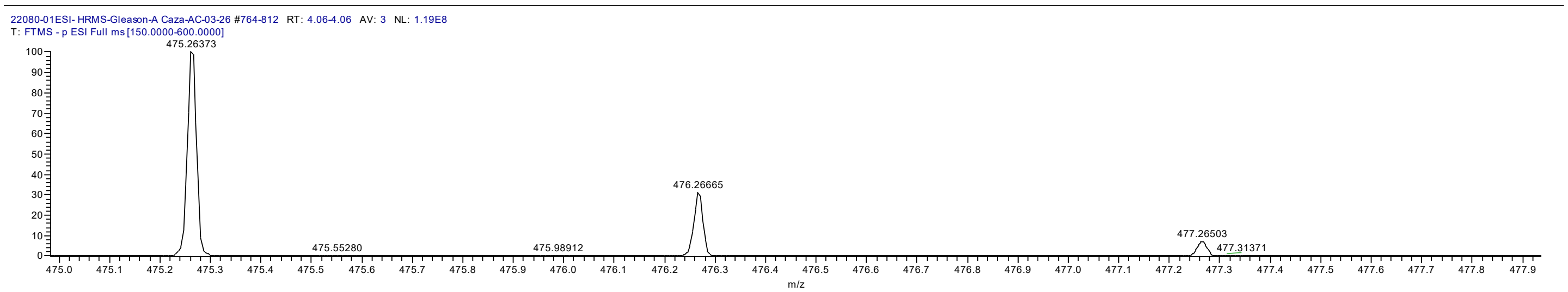

22080-01ESI- HRMS-Gleason-A Caza-AC-03-26#810-812 RT: 4.06-4.06 AV: 3
T: FTMS - p ESI Full ms [150.0000-600.0000]
m/z= 475.24202-475.27848

m/z	Intensity	Relative	Resolution	Charge	Theo. Mass	Delta (ppm)	RDB equiv.	Composition
475.26373	123322912.0	100.00	23883.57	1.00	475.26360	0.28	8.5	$C_{26}H_{39}O_4N_2S$
					475.26543	-3.57	3.5	$C_{25}H_{44}O_4ClS$

^{1}H NMR of **7b** (500 MHz, DMSO-d6)

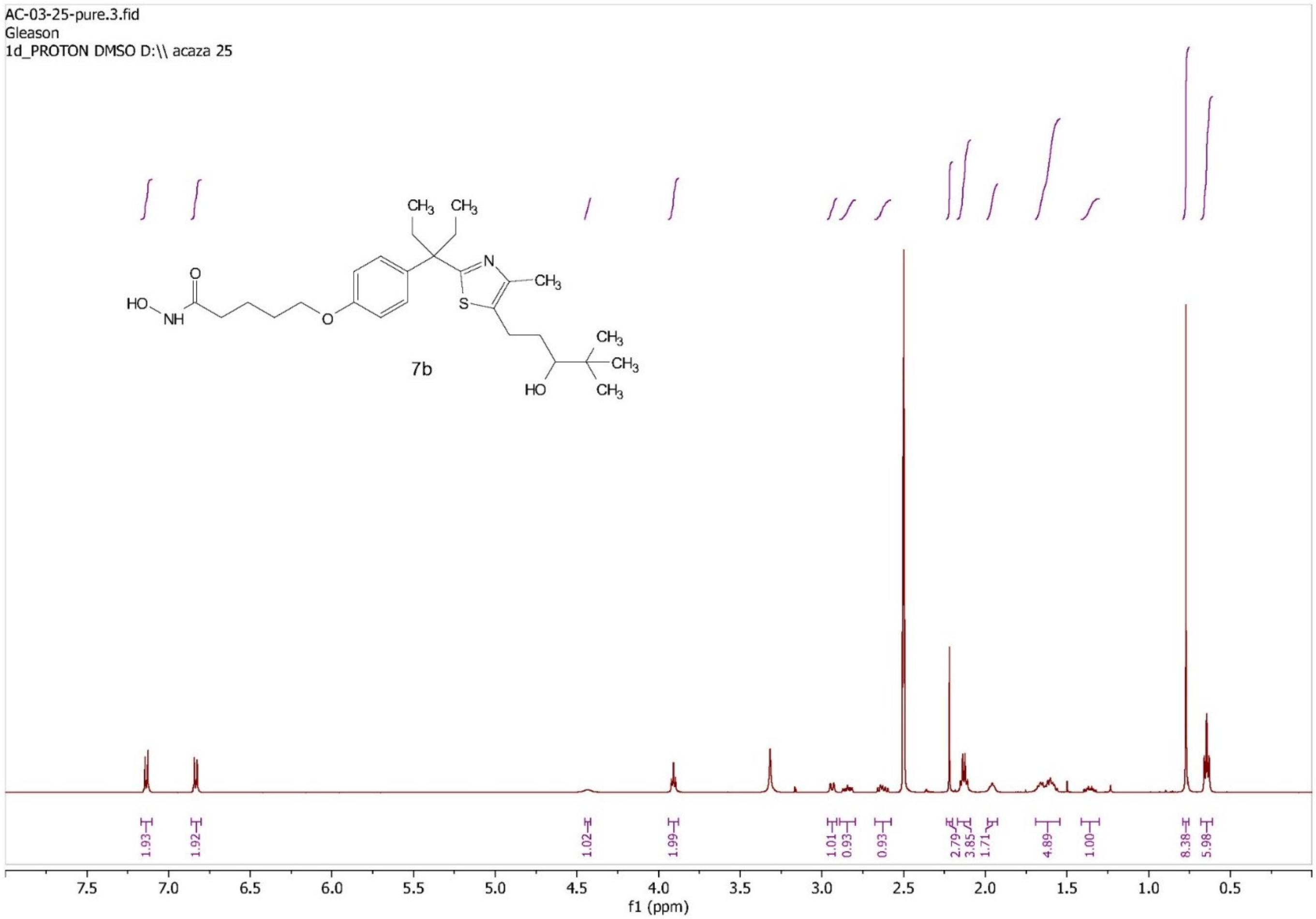

^{13}C NMR of **7b** (126 MHz, DMSO-d6)

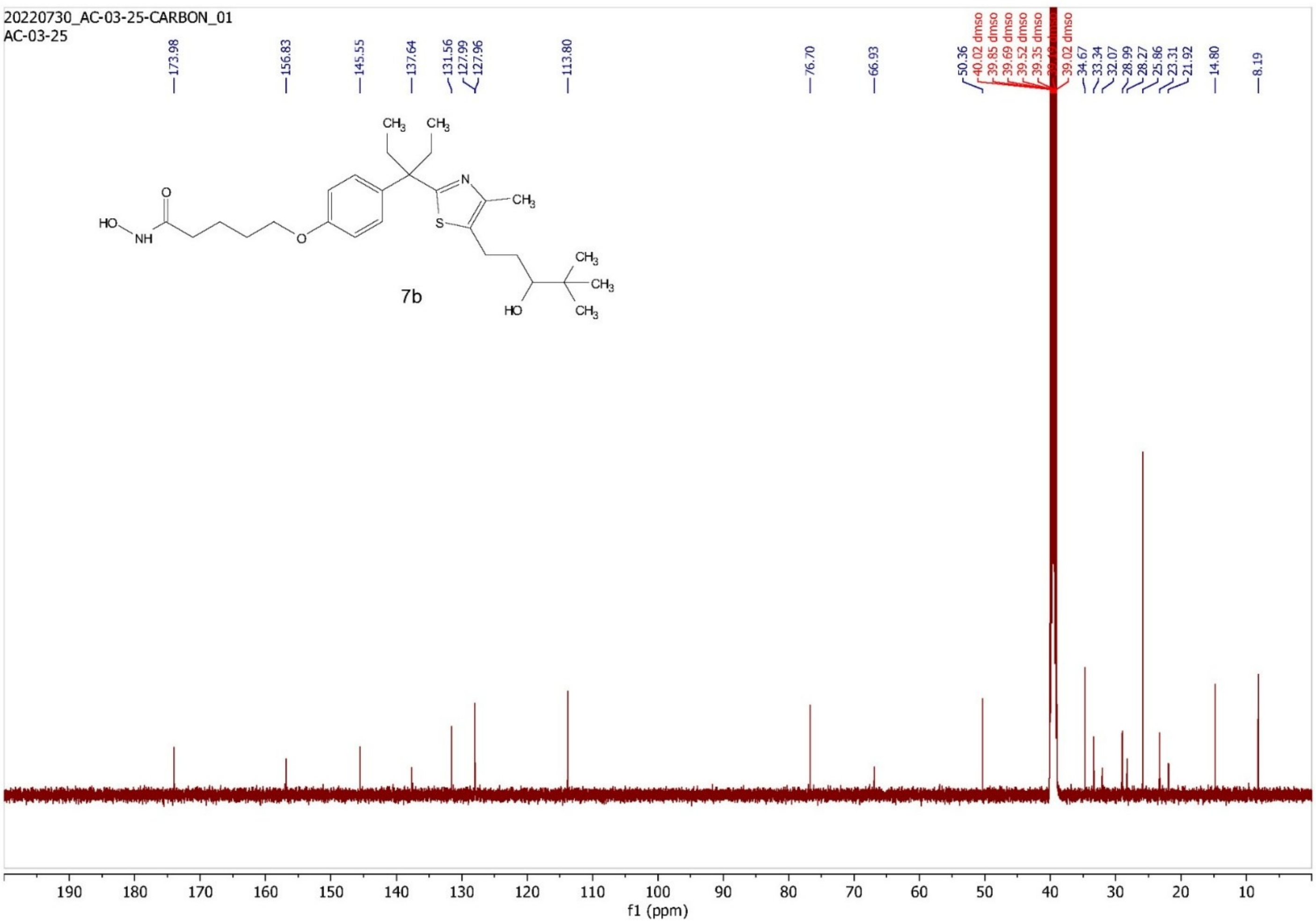

HMBC of **7b** (DMSO-d6)

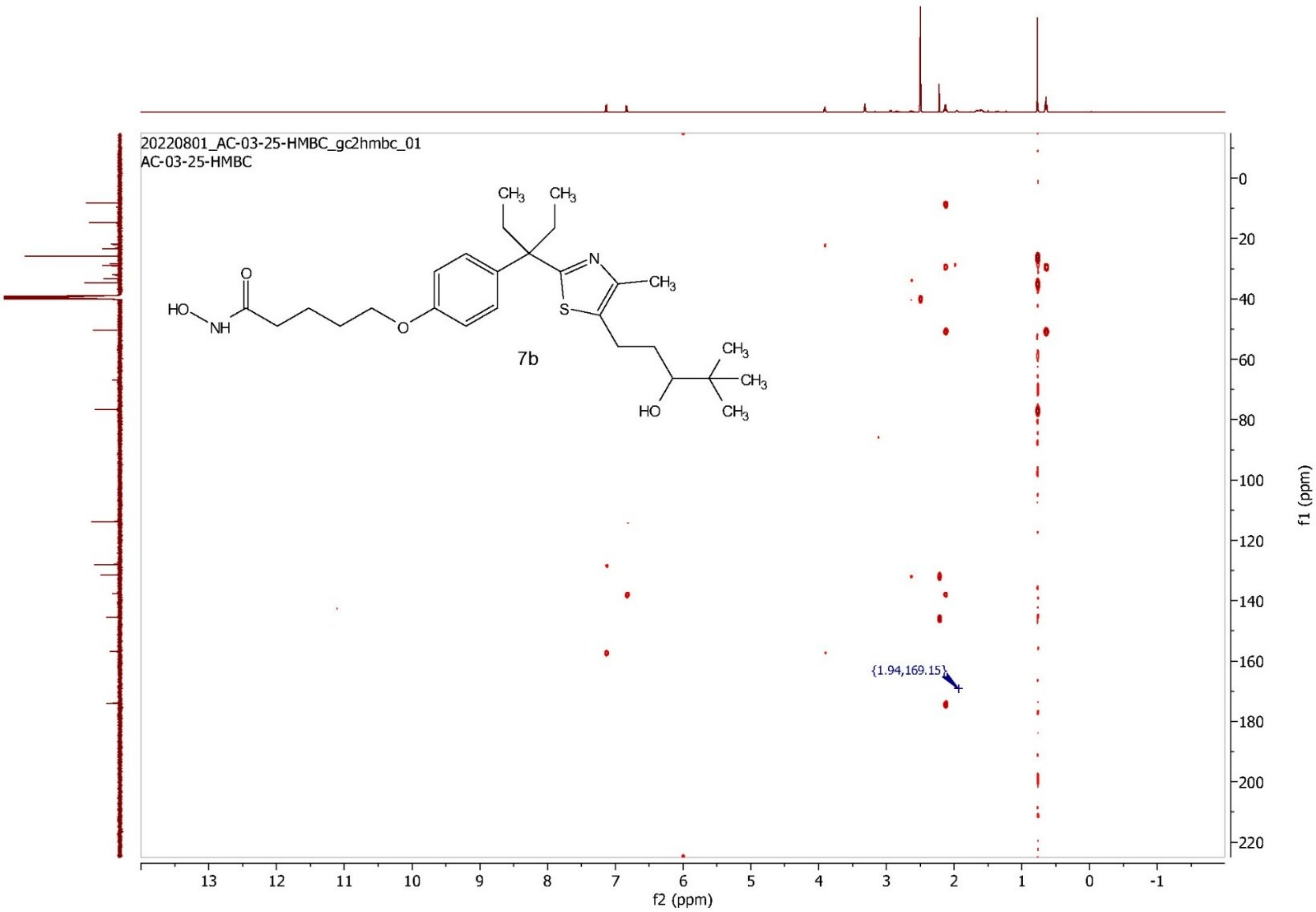

HRMS of **7b**

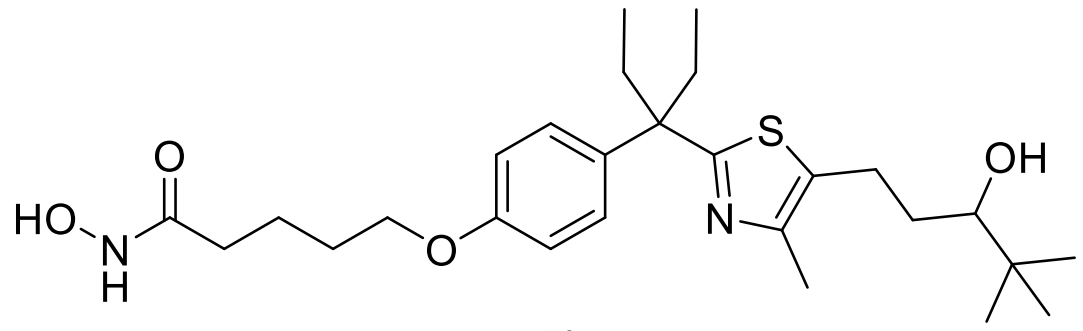

7b

220729-08ESI- HRMS-Gleason- 07/29/22 11:30:19

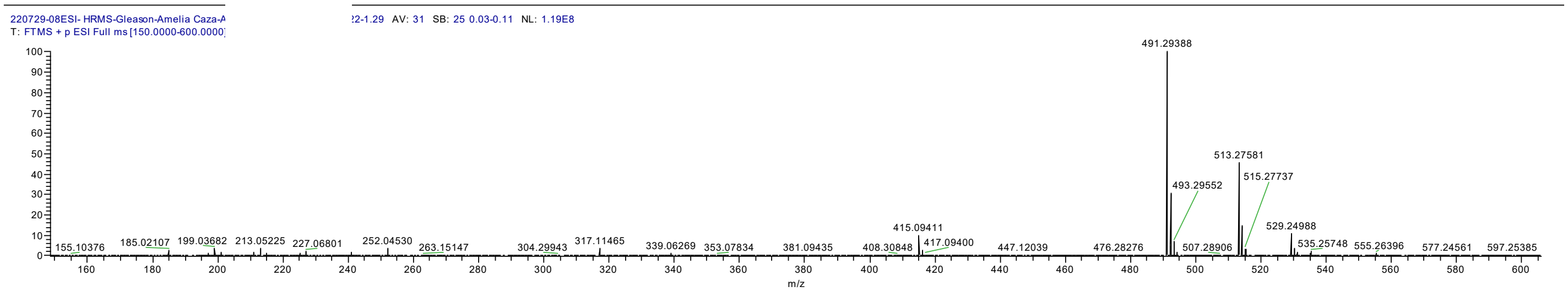

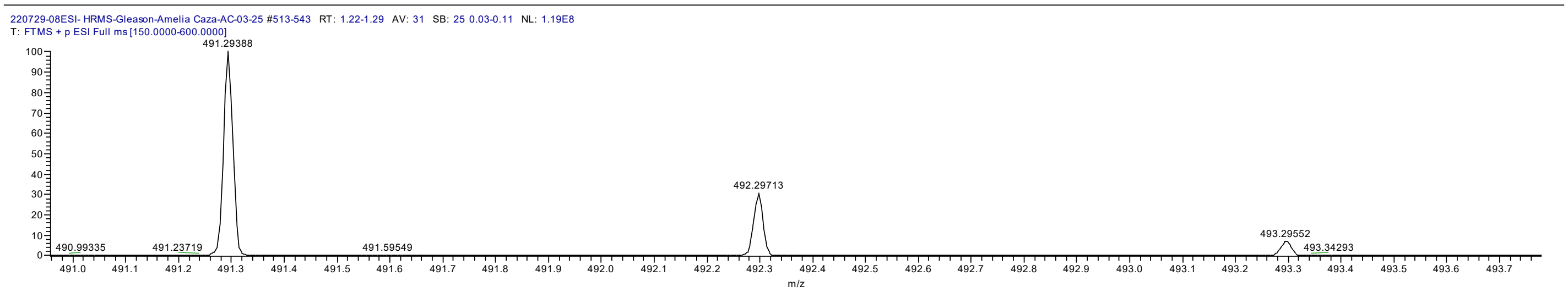

220729-08ESI- HRMS-Gleason-Amelia Caza-AC-03-25#513-543 RT: 1.22-1.29 AV: 31
SB: 25 0.03-0.11
T: FTMS + p ESI Full ms [150.0000-600.0000]
m/z= 491.26646-491.31127

m/z	Intensity	Relative	Resolution	Charge	Theo. Mass	Delta (ppm)	RDB equiv.	Composition
491.29388	118774824.0	100.00	25500.22	1.00	491.29380	0.15	7.5	$C_{27}H_{43}O_4N_2S$

^{1}H NMR of **7c** (500 MHz, DMSO-d6)

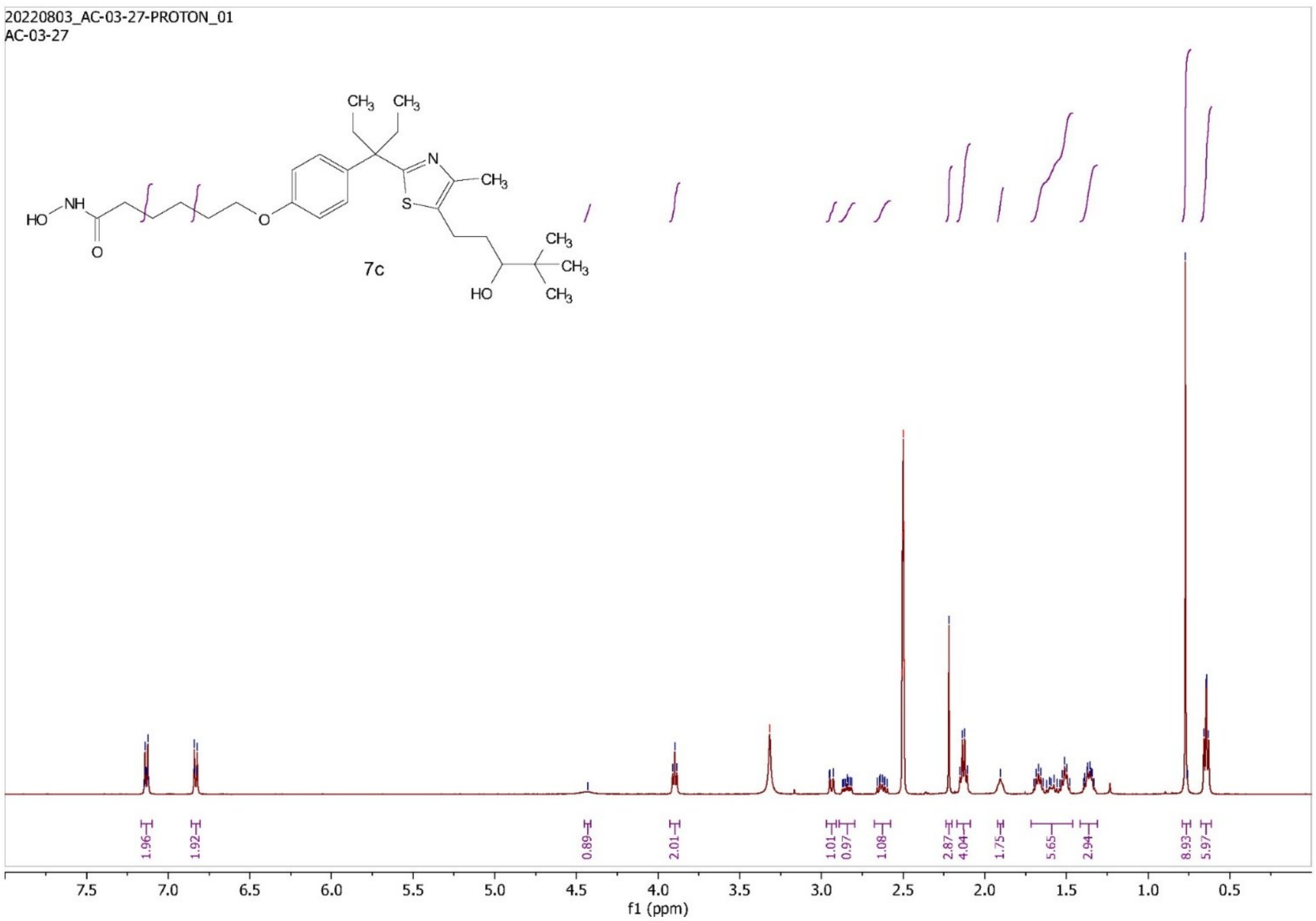

^{13}C NMR of **7c** (126 MHz, DMSO-d6)

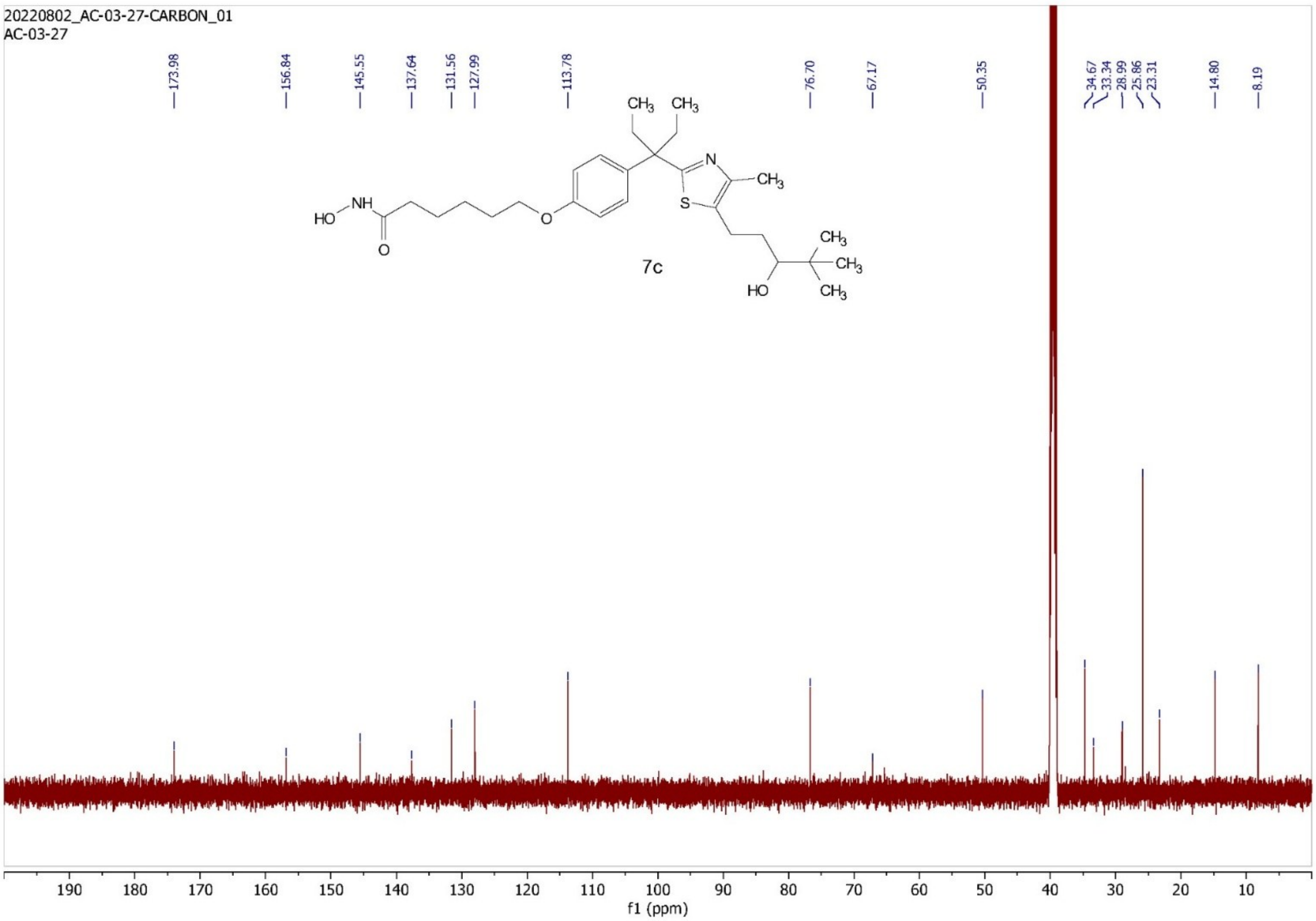

HMBC of **7c** (DMSO-d6)

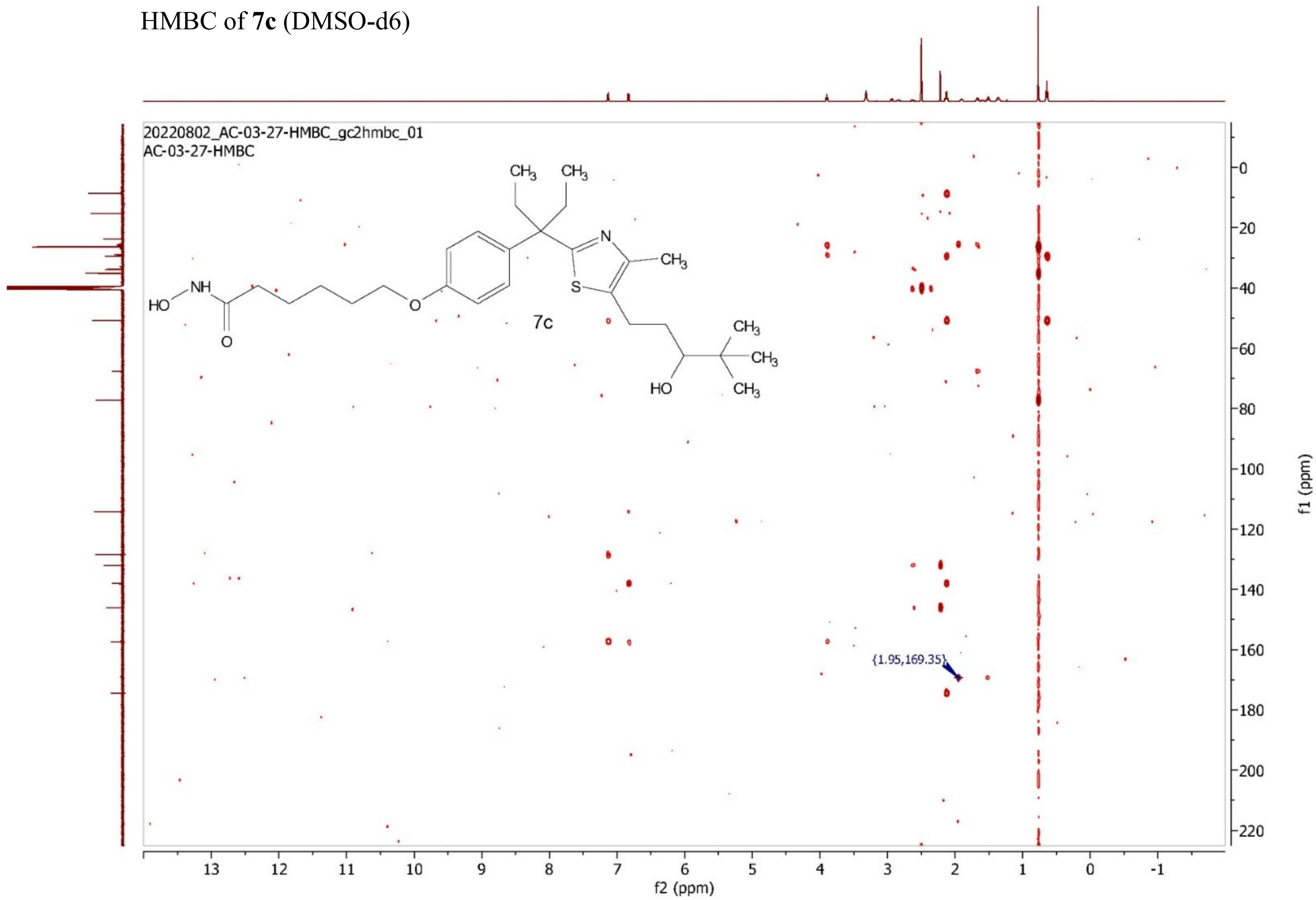

HRMS of **7c**

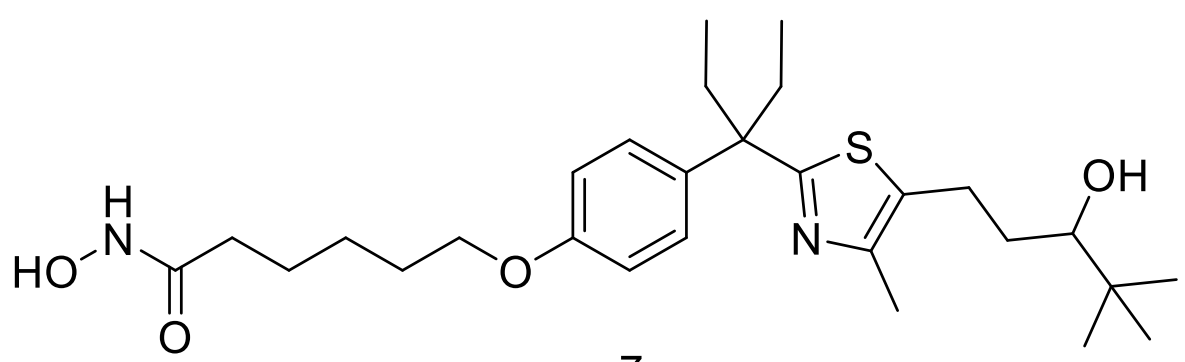

7c

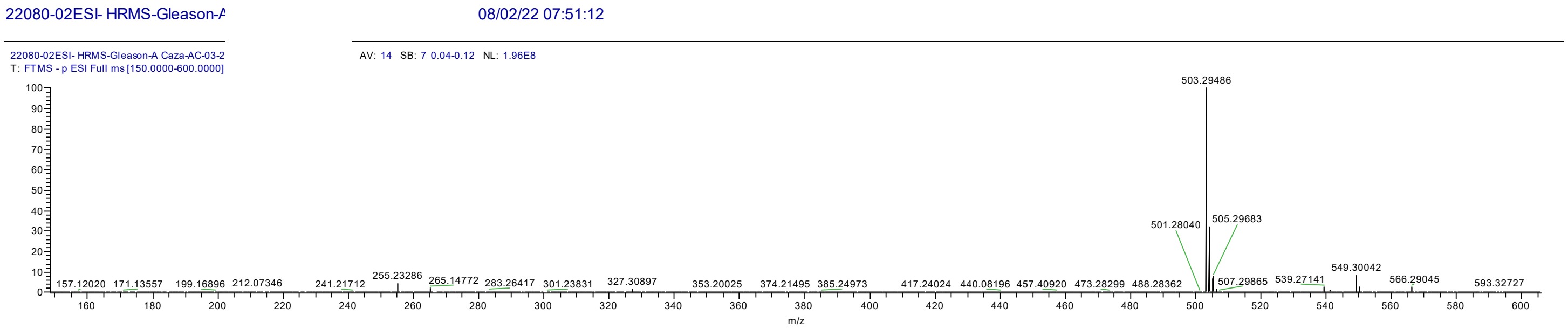

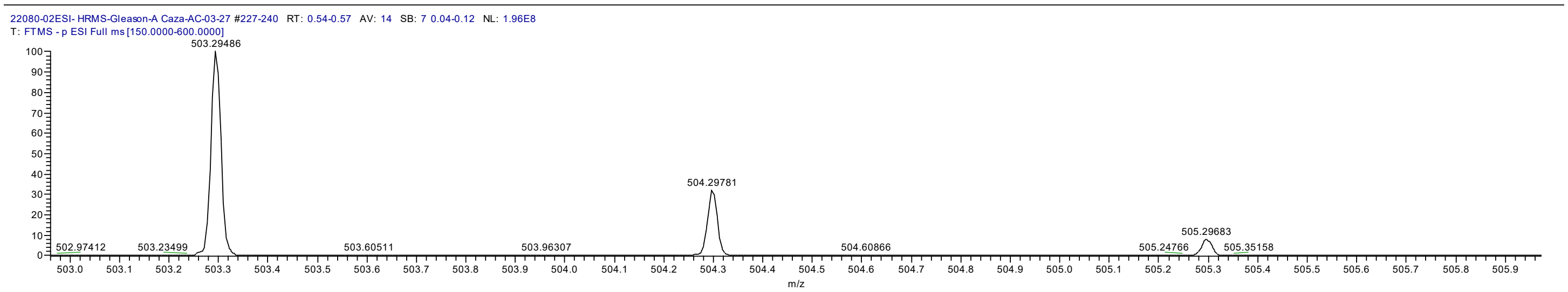

22080-02ESI- HRMS-Gleason-A Caza-AC-03-27#227-240 RT: 0.54-0.57 AV: 14
SB: 7 0.04-0.12
T: FTMS - p ESI Full ms [150.0000-600.0000]
m/z= 503.27475-503.31660

m/z	Intensity	Relative	Resolution	Charge	Theo. Mass	Delta (ppm)	RDB equiv.	Composition
503.29486	196807264.0	100.00	23461.51	1.00	503.29490	-0.09	8.5	$C_{28}H_{43}O_4N_2S$
					503.29673	-3.72	3.5	$C_{27}H_{48}O_4ClS$

HPLC reports of 7a (AC-340), 7b (AC-339), and 7c (AC-341)

HPLC report of **7a**

Acquisition Experiment Report   MassLynx 4.1 SCN805   Page 1 of 5

File: C:\MASSLYNX\DEFAULT.PRO\DATA\AC-03-26-PURE.RAW

Printed: Monday, June 30, 2025 13:01:45 Eastern Daylight Time

---

Header

```
Acquired File Name:   AC-03-26-pure
Acquired Date:        15-Aug-2022
Acquired Time:        12:14:15
Job Code:             AC-03-26-pure
Task Code:
User Name:            Ryan
Laboratory Name:
Instrument:           W3100#MBA486
Conditions:
Submitter:            Ryan
SampleID:             AC-03-26-pure
Bottle Number:        74
Description:          AC-03-26-pure

Instrument Calibration:
Calibration File: C:\MassLynx\IntelliStart\Results\Unit Mass Resolution\Calibration_20210304
Parameters
MS1 Static:       None
MS1 Scanning:     None
MS1 Scan Speed Compensation:   None
MS2 Static:       None
MS2 Scanning:     None
MS2 Scan Speed Compensation:   None
Calibration Time:
Calibration Date:
Coefficients
MS1 Static:       None
MS2 Static:       None
Function 1:       None
Function 2:       None
Function 3:       None

Parameters for C:\MassLynx\DEFAULT.PRO\ACQUDB\POS_NEG_100_1000_15min_waste_2 min.EXP

Data Processing:
SIR / MRM Chromatogram Spike Removal    ON
SIR / MRM Smoothing                OFF
Smoothing window size (scans)      3
Number of smooths                  2

Prescan Statistics:
Initial Average Intensity          7.0222
Initial Average Std Dev            1.7903
Bunch Zero Level                   0.0040
Bunch Std Dev                      0.0105
Bunch Threshold                    0.0838
Spike Removal Std Dev              1.7055

Method Events:
Initial Stop Flow:      No Change
Initial Switch 2:       No Change
Initial Switch 3:       No Change
Initial Switch 4:       No Change
```

Create PDF files without this message by purchasing novaPDF printer (http://www.novapdf.com)

```
Initial Infusion:        No Change
Initial Flow State:      Waste
Initial Flow Rate:       5
Initial Reservoir:       No Action
API Probe Delay Temp:    20
Initial Refill:          No Action
Timed Events Enabled
Event   Time    Name            Action
1       0       Flow State              Waste
2       120     Flow State              LC


Instrument Parameters - Function 1:
Parameter File - C:\MassLynx\Default.pro\Acqudb\Standard_Tune_POS_NEG.IPR
Polarity        ES+
Calibration     Dynamic 1
Capillary (kV)  3.00    2.82
Cone (V)        25.00   9.52
Extractor (V)   3.00    1.22
RF (V)  0.10
Source Temperature (°C) 150     148
Desolvation Temperature (°C)    475     468
Cone Gas Flow (L/Hr)    80      80
Desolvation Gas Flow (L/Hr)     950     977
LM 1 Resolution 8.40
HM 1 Resolution 15.00
Ion Energy 1    0.70
Gain    1.00
Multiplier      -547.97
Active Reservoir        B

Instrument Parameters - Function 2:
Parameter File - C:\MassLynx\Default.pro\Acqudb\Standard_Tune_POS_NEG.IPR
Polarity        ES-
Calibration     Dynamic 1
Capillary (kV)  2.80    2.82
Cone (V)        25.00   9.52
Extractor (V)   3.00    1.22
RF (V)  0.10
Source Temperature (°C) 150     148
Desolvation Temperature (°C)    475     468
Cone Gas Flow (L/Hr)    80      80
Desolvation Gas Flow (L/Hr)     1000    977
LM 1 Resolution 8.40
HM 1 Resolution 15.00
Ion Energy 1    0.70
Gain    1.00
Multiplier      -547.97
Active Reservoir        B


Engineers Settings:
Low Mass Position               518
High Mass Position              426
Low Mass Resolution             517
High Mass Resolution            276
Resolution Linearity            830
High Mass DC Balance            0
```

Create PDF files without this message by purchasing novaPDF printer (http://www.novapdf.com)

DC Polarity Positive
HM RF Lens Correction + 0
HM RF Lens Correction - 0

Inter-scan delays:
Automatic Mode
MS Delay Table

	R	delay
<=	1.000	0.005
<=	3.000	0.010
<=	7.000	0.015
<=	17.000	0.020
>	17.000	0.025

ACE Experimental Record

Inlet Method File: c:\masslynx\default.pro\acqudb\5_95mecn_19_min_fa

--------------------- Run method parameters ----------------

Waters Alliance 2695 HPLC Pump Initial Conditions

Solvents
Degasser NormalStroke Volume 130.0 µlFinal Flow Settings being held at End of Run
B% 5.0 MeCN + 0.1% Formic
C% 0.0 Solvent C
D% 0.0 Solvent D
Flow (ml/min) 1.000
Flow Ramp 2.00
Stop Time (mins) 5.0
Column Temperature (°C) 35.0
Column Temperature Limit (°C) 5.0
Min Pressure (Bar) 0.0
Max Pressure (Bar) 300.0
Pre-column Volume (µl) 0.00
Column Position Column 1Pre-column Volume (µl) 0.00

Waters Alliance 2695 HPLC Pump Gradient Timetable

The gradient Timetable contains 6 entries which are :

		Time	A%	B%	C%	D%	Flow	Curve
0.00	95.0	5.0	0.0	0.0	1.000	1		
1.00	95.0	5.0	0.0	0.0	1.000	6		
13.00	0.0	100.0	0.0	0.0	1.000	6		
14.50	0.0	100.0	0.0	0.0	1.000	1		
15.00	95.0	5.0	0.0	0.0	1.000	1		
18.50	95.0	5.0	0.0	0.0	0.100	11		

Waters Alliance 2695 HPLC Pump External Event Timetable

No Entries in the Pump External Event Timetable.

Create PDF files without this message by purchasing novaPDF printer (http://www.novapdf.com)

Waters 2489

Run Time: 14.50 min
Wavelength Mode: Dual Wavelength
Lamp On: On
Channel A...
Comment:
Wavelength: 254 nm
Sampling Rate: 2 points/sec
Data Mode: Absorbance A
Time Constant: 1.0000 sec
Auto Zero On Wavelength Change: Maintain Baseline
Auto Zero On Inject Start: Yes
Channel B...
Comment:
Wavelength: 220 nm
Sampling Rate: 2 points/sec
Data Mode: Absorbance B
Time Constant: 1.0000 sec
Auto Zero On Wavelength Change: Maintain Baseline
Auto Zero On Inject Start: Yes
Analog 1...
Sensitivity: 2.000 AUFS
Chart Polarity: Positive (+)
Voltage Offset: 0 mV
Enable Chart Mark: Yes
Analog 2...
Sensitivity: 2.000 AUFS
Chart Polarity: Positive (+)
Voltage Offset: 0 mV
Enable Chart Mark: Yes
Run Events: Yes
Pulse Width: 1.0 sec
Rect Wave Period: 0.2 sec

Waters Alliance 2695 Autosampler Initial Conditions

Needle Depth (mm)	3.00
Sample Temperature (°C)	20.0
Sample Temperature Limit (°C)	20.0
Purge Loop Volumes	0.00

Sample Run Injection Parameter

Injection Volume (ul) - 10.00

---------------------------- oOo ----------------------------

Create PDF files without this message by purchasing novaPDF printer (http://www.novapdf.com)

End of experimental record.------------------- Generic Instrument Postrun Report ----------

Software Version: 1.30.2065
Firmware Version: 1.40.1966 (Dec 12 2008)
Checksum: 0xe45cd0c3
Serial Number: D1187E056A
Lamp Changed: No
Lamp Life: 20887.00

---------------------------- oOo ----------------------------

Function 1

Scans in function:	1969
Cycle time (secs):	Automatic
Scan duration (secs):	0.200
Inter Scan Delay (secs):	Automatic
Start and End Time(mins):	0.000 to 14.500
Ionization mode:	ES+
Data type:	Enhanced Mass
Function type:	Scan
Mass range:	100 to 1000

Function 2

Scans in function:	1968
Cycle time (secs):	Automatic
Scan duration (secs):	0.200
Inter Scan Delay (secs):	Automatic
Start and End Time(mins):	0.000 to 14.500
Ionization mode:	ES-
Data type:	Enhanced Mass
Function type:	Scan
Mass range:	100 to 1000

Function 3

Scans in function:	1741
Function type:	Diode Array
Wavelength range (nm):	220 to 254

Create PDF files without this message by purchasing novaPDF printer (http://www.novapdf.com)

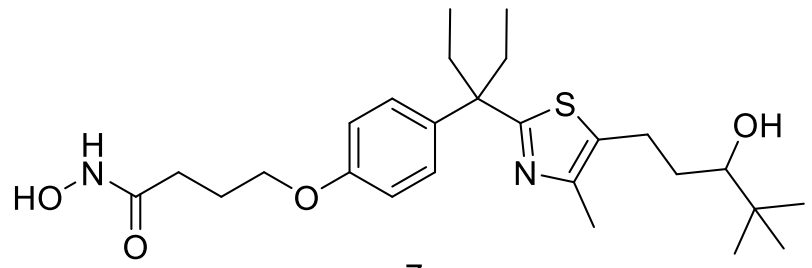

7a

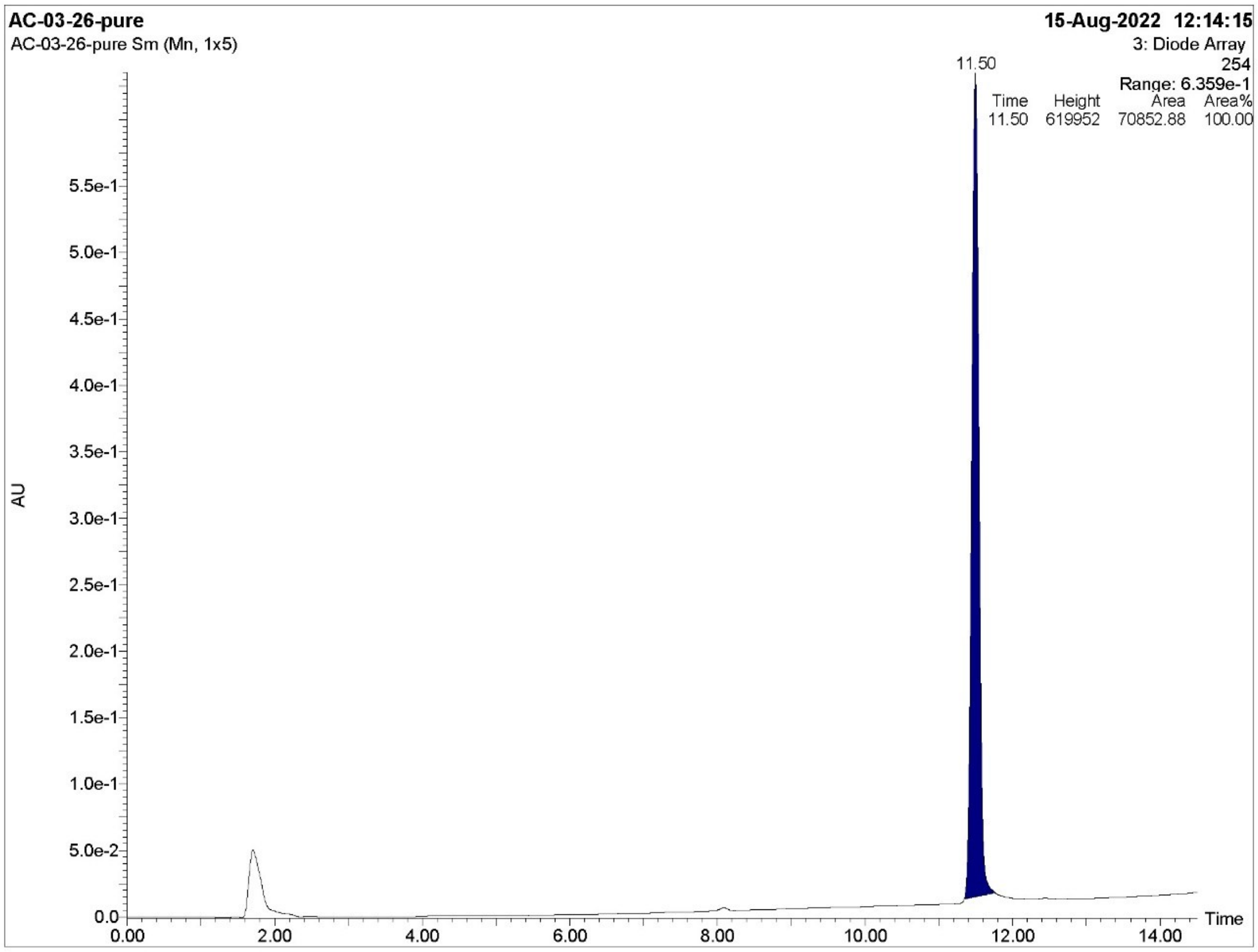

Create PDF files without this message by purchasing novaPDF printer (http://www.novapdf.com)

HPLC report of **7b**

Acquisition Experiment Report MassLynx 4.1 SCN805 **Page 1 of 5**

File: **C:\MASSLYNX\DEFAULT.PRO\DATA\AC-03-25.RAW**

Printed: **Monday, June 30, 2025 13:01:21 Eastern Daylight Time**

Header

```
Acquired File Name:   AC-03-25
Acquired Date:        28-Jul-2022
Acquired Time:        17:13:16
Job Code:             Ryan-AC-03-25
Task Code:
User Name:            Ryan
Laboratory Name:
Instrument:           W3100#MBA486
Conditions:
Submitter:            Ryan
SampleID:             AC-03-25
Bottle Number:        15
Description:          rp pure

Instrument Calibration:
Calibration File: C:\MassLynx\IntelliStart\Results\Unit Mass Resolution\Calibration_20210304
Parameters
MS1 Static:       None
MS1 Scanning:     None
MS1 Scan Speed Compensation:   None
MS2 Static:       None
MS2 Scanning:     None
MS2 Scan Speed Compensation:   None
Calibration Time:
Calibration Date:
Coefficients
MS1 Static:       None
MS2 Static:       None
Function 1:       None
Function 2:       None
Function 3:       None


Parameters for C:\MassLynx\DEFAULT.PRO\ACQUDB\POS_NEG_100_1000_15min_waste_2 min.EXP

Data Processing:
SIR / MRM Chromatogram Spike Removal    ON
SIR / MRM Smoothing                  OFF
Smoothing window size (scans)        3
Number of smooths                    2


Prescan Statistics:
Initial Average Intensity       7.0793
Initial Average Std Dev         1.8935
Bunch Zero Level                0.0063
Bunch Std Dev                   0.0127
Bunch Threshold                 0.1013
Spike Removal Std Dev           1.7167


Method Events:
Initial Stop Flow:      No Change
Initial Switch 2:       No Change
Initial Switch 3:       No Change
Initial Switch 4:       No Change
```

Create PDF files without this message by purchasing novaPDF printer (http://www.novapdf.com)

```
Initial Infusion:       No Change
Initial Flow State:     Waste
Initial Flow Rate:      5
Initial Reservoir:      No Action
API Probe Delay Temp:   20
Initial Refill:         No Action
Timed Events Enabled
Event   Time    Name            Action
1       0       Flow State              Waste
2       120     Flow State              LC


Instrument Parameters - Function 1:
Parameter File - C:\MassLynx\Default.pro\Acqudb\Standard_Tune_POS_NEG.IPR
Polarity        ES+
Calibration     Dynamic 1
Capillary (kV)  3.00    2.81
Cone (V)        25.00   8.55
Extractor (V)   3.00    0.73
RF (V)  0.10
Source Temperature (°C) 150     148
Desolvation Temperature (°C)    475     468
Cone Gas Flow (L/Hr)    80      81
Desolvation Gas Flow (L/Hr)     950     981
LM 1 Resolution 8.40
HM 1 Resolution 15.00
Ion Energy 1    0.70
Gain    1.00
Multiplier      -548.75
Active Reservoir        B

Instrument Parameters - Function 2:
Parameter File - C:\MassLynx\Default.pro\Acqudb\Standard_Tune_POS_NEG.IPR
Polarity        ES-
Calibration     Dynamic 1
Capillary (kV)  2.80    2.81
Cone (V)        25.00   8.55
Extractor (V)   3.00    0.73
RF (V)  0.10
Source Temperature (°C) 150     148
Desolvation Temperature (°C)    475     468
Cone Gas Flow (L/Hr)    80      81
Desolvation Gas Flow (L/Hr)     1000    981
LM 1 Resolution 8.40
HM 1 Resolution 15.00
Ion Energy 1    0.70
Gain    1.00
Multiplier      -548.75
Active Reservoir        B


Engineers Settings:
Low Mass Position               518
High Mass Position              426
Low Mass Resolution             517
High Mass Resolution            276
Resolution Linearity            830
High Mass DC Balance            0
```

Create PDF files without this message by purchasing novaPDF printer (http://www.novapdf.com)

```
DC Polarity                     Positive
HM RF Lens Correction +         0
HM RF Lens Correction -         0


Inter-scan delays:
Automatic Mode
MS Delay Table
          R      delay
<=    1.000      0.005
<=    3.000      0.010
<=    7.000      0.015
<=   17.000      0.020
>    17.000      0.025



ACE Experimental Record


Inlet Method File: c:\masslynx\default.pro\acqudb\5_95mecn_19_min_fa

---------------------   Run method parameters   ----------------



Waters Alliance 2695 HPLC Pump Initial Conditions

Solvents
Degasser        NormalStroke Volume     130.0 µlFinal Flow Settings being held at End of Run
B%                                              5.0 MeCN + 0.1% Formic
C%                                              0.0 Solvent C
D%                                              0.0 Solvent D
Flow (ml/min)                                   1.000
Flow Ramp                                       2.00
Stop Time (mins)                                5.0
Column Temperature (°C)                         35.0
Column Temperature Limit (°C)                   5.0
Min Pressure (Bar)                              0.0
Max Pressure (Bar)                              300.0
Pre-column Volume (µl)                          0.00
Column Position         Column 1Pre-column Volume (µl)                         0.00

Waters Alliance 2695 HPLC Pump Gradient Timetable

The gradient Timetable contains 6 entries which are :

                Time      A%      B%      C%      D%    Flow    Curve
0.00    95.0    5.0     0.0     0.0     1.000     1
1.00    95.0    5.0     0.0     0.0     1.000     6
13.00   0.0     100.0   0.0     0.0     1.000     6
14.50   0.0     100.0   0.0     0.0     1.000     1
15.00   95.0    5.0     0.0     0.0     1.000     1
18.50   95.0    5.0     0.0     0.0     0.100     11

Waters Alliance 2695 HPLC Pump External Event Timetable

No Entries in the Pump External Event Timetable.
```

Create PDF files without this message by purchasing novaPDF printer (http://www.novapdf.com)

Waters 2489

Run Time: 14.50 min
Wavelength Mode: Dual Wavelength
Lamp On: On
Channel A...
Comment:
Wavelength: 254 nm
Sampling Rate: 2 points/sec
Data Mode: Absorbance A
Time Constant: 1.0000 sec
Auto Zero On Wavelength Change: Maintain Baseline
Auto Zero On Inject Start: Yes
Channel B...
Comment:
Wavelength: 220 nm
Sampling Rate: 2 points/sec
Data Mode: Absorbance B
Time Constant: 1.0000 sec
Auto Zero On Wavelength Change: Maintain Baseline
Auto Zero On Inject Start: Yes
Analog 1...
Sensitivity: 2.000 AUFS
Chart Polarity: Positive (+)
Voltage Offset: 0 mV
Enable Chart Mark: Yes
Analog 2...
Sensitivity: 2.000 AUFS
Chart Polarity: Positive (+)
Voltage Offset: 0 mV
Enable Chart Mark: Yes
Run Events: Yes
Pulse Width: 1.0 sec
Rect Wave Period: 0.2 sec

Waters Alliance 2695 Autosampler Initial Conditions

Needle Depth (mm)	3.00
Sample Temperature (°C)	20.0
Sample Temperature Limit (°C)	20.0
Purge Loop Volumes	0.00

Sample Run Injection Parameter

Injection Volume (ul) - 10.00

---------------------------- oOo ----------------------------

Create PDF files without this message by purchasing novaPDF printer (http://www.novapdf.com)

End of experimental record.------------------- Generic Instrument Postrun Report ----------

Software Version: 1.30.2065
Firmware Version: 1.40.1966 (Dec 12 2008)
Checksum: 0xe45cd0c3
Serial Number: D1187E056A
Lamp Changed: No
Lamp Life: 20727.00

---------------------------- oOo ----------------------------

Function 1

Scans in function:	1969
Cycle time (secs):	Automatic
Scan duration (secs):	0.200
Inter Scan Delay (secs):	Automatic
Start and End Time(mins):	0.000 to 14.500
Ionization mode:	ES+
Data type:	Enhanced Mass
Function type:	Scan
Mass range:	100 to 1000

Function 2

Scans in function:	1968
Cycle time (secs):	Automatic
Scan duration (secs):	0.200
Inter Scan Delay (secs):	Automatic
Start and End Time(mins):	0.000 to 14.500
Ionization mode:	ES-
Data type:	Enhanced Mass
Function type:	Scan
Mass range:	100 to 1000

Function 3

Scans in function:	1741
Function type:	Diode Array
Wavelength range (nm):	220 to 254

Create PDF files without this message by purchasing novaPDF printer (http://www.novapdf.com)

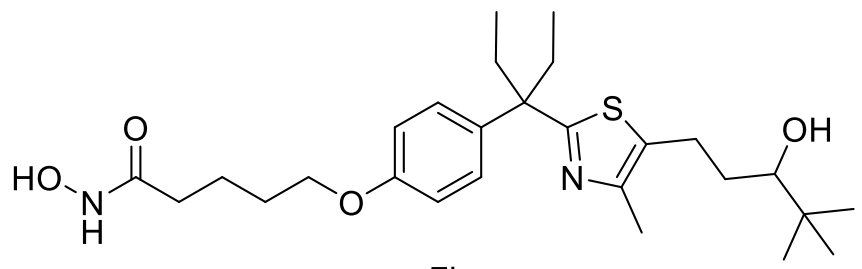

7b

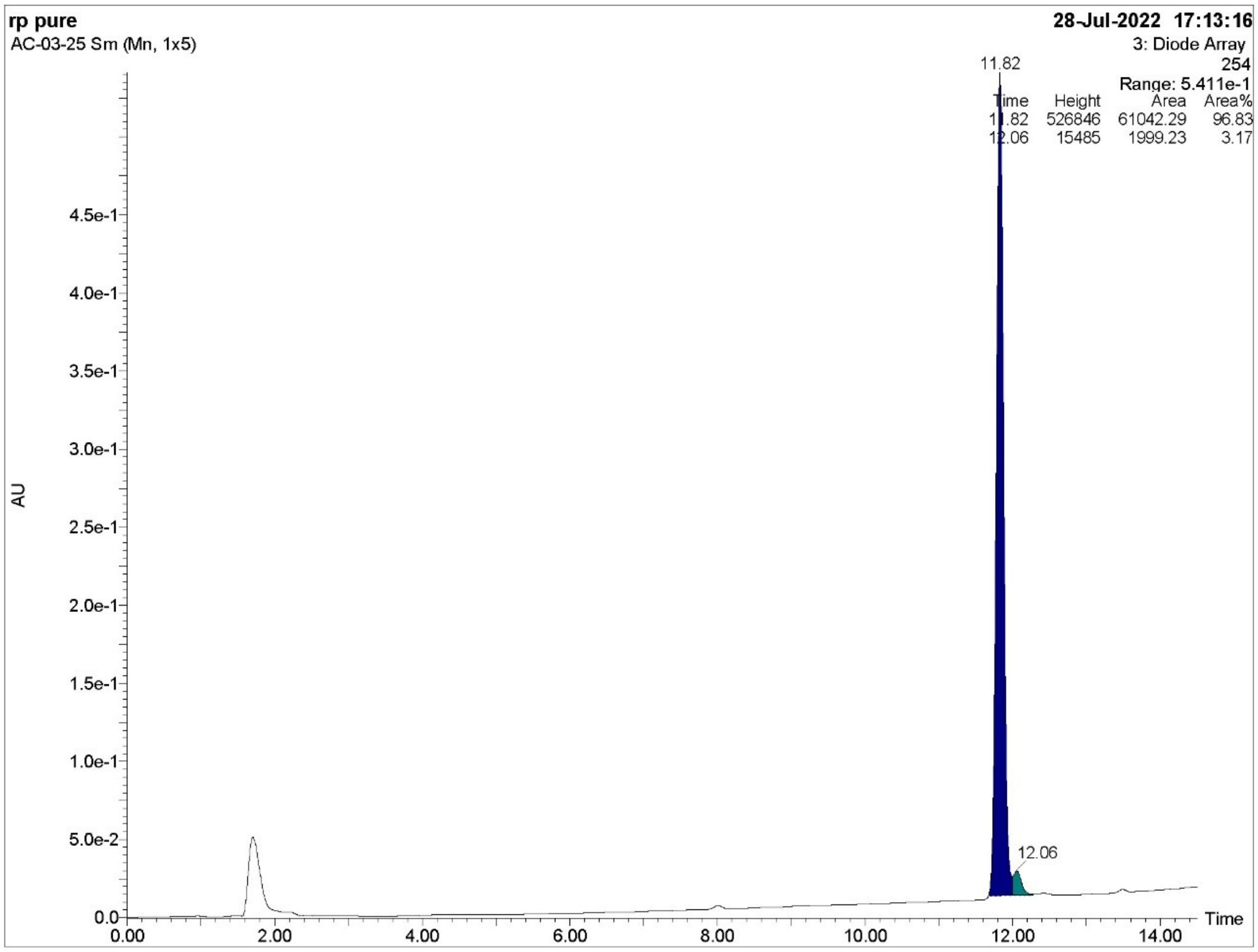

Create PDF files without this message by purchasing novaPDF printer (http://www.novapdf.com)

HPLC report of **7c**

Acquisition Experiment Report MassLynx 4.1 SCN805

File: **C:\MASSLYNX\DEFAULT.PRO\DATA\AC-03-27.RAW**

Printed: **Monday, June 30, 2025 13:03:54 Eastern Daylight Time**

Header

```
Acquired File Name:   AC-03-27
Acquired Date:        01-Aug-2022
Acquired Time:        21:18:50
Job Code:             RyanAC-03-27
Task Code:
User Name:            Ryan
Laboratory Name:
Instrument:           W3100#MBA486
Conditions:
Submitter:            Ryan
SampleID:             AC-03-27
Bottle Number:        72
Description:          rp pure

Instrument Calibration:
Calibration File: C:\MassLynx\IntelliStart\Results\Unit Mass Resolution\Calibration_20210304
Parameters
MS1 Static:       None
MS1 Scanning:     None
MS1 Scan Speed Compensation:   None
MS2 Static:       None
MS2 Scanning:     None
MS2 Scan Speed Compensation:   None
Calibration Time:
Calibration Date:
Coefficients
MS1 Static:       None
MS2 Static:       None
Function 1:       None
Function 2:       None
Function 3:       None


Parameters for C:\MassLynx\DEFAULT.PRO\ACQUDB\POS_NEG_100_1000_15min_waste_2 min.EXP

Data Processing:
SIR / MRM Chromatogram Spike Removal    ON
SIR / MRM Smoothing                OFF
Smoothing window size (scans)      3
Number of smooths                  2


Prescan Statistics:
Initial Average Intensity          7.0660
Initial Average Std Dev            1.8517
Bunch Zero Level                   0.0054
Bunch Std Dev                      0.0116
Bunch Threshold                    0.0930
Spike Removal Std Dev              1.7127


Method Events:
Initial Stop Flow:      No Change
Initial Switch 2:       No Change
Initial Switch 3:       No Change
Initial Switch 4:       No Change
```

Create PDF files without this message by purchasing novaPDF printer (http://www.novapdf.com)

```
Initial Infusion:       No Change
Initial Flow State:     Waste
Initial Flow Rate:      5
Initial Reservoir:      No Action
API Probe Delay Temp:   20
Initial Refill:         No Action
Timed Events Enabled
Event   Time    Name            Action
1       0       Flow State              Waste
2       120     Flow State              LC


Instrument Parameters - Function 1:
Parameter File - C:\MassLynx\Default.pro\Acqudb\Standard_Tune_POS_NEG.IPR
Polarity        ES+
Calibration     Dynamic 1
Capillary (kV)  3.00    2.81
Cone (V)        25.00   9.04
Extractor (V)   3.00    0.73
RF (V)  0.10
Source Temperature (°C) 150     148
Desolvation Temperature (°C)    475     475
Cone Gas Flow (L/Hr)    80      80
Desolvation Gas Flow (L/Hr)     950     978
LM 1 Resolution 8.40
HM 1 Resolution 15.00
Ion Energy 1    0.70
Gain    1.00
Multiplier      -547.97
Active Reservoir        B

Instrument Parameters - Function 2:
Parameter File - C:\MassLynx\Default.pro\Acqudb\Standard_Tune_POS_NEG.IPR
Polarity        ES-
Calibration     Dynamic 1
Capillary (kV)  2.80    2.81
Cone (V)        25.00   9.04
Extractor (V)   3.00    0.73
RF (V)  0.10
Source Temperature (°C) 150     148
Desolvation Temperature (°C)    475     475
Cone Gas Flow (L/Hr)    80      80
Desolvation Gas Flow (L/Hr)     1000    978
LM 1 Resolution 8.40
HM 1 Resolution 15.00
Ion Energy 1    0.70
Gain    1.00
Multiplier      -547.97
Active Reservoir        B


Engineers Settings:
Low Mass Position               518
High Mass Position              426
Low Mass Resolution             517
High Mass Resolution            276
Resolution Linearity            830
High Mass DC Balance            0
```

Create PDF files without this message by purchasing novaPDF printer (http://www.novapdf.com)

DC Polarity Positive
HM RF Lens Correction + 0
HM RF Lens Correction - 0

Inter-scan delays:
Automatic Mode
MS Delay Table

	R	delay
<=	1.000	0.005
<=	3.000	0.010
<=	7.000	0.015
<=	17.000	0.020
>	17.000	0.025

ACE Experimental Record

Inlet Method File: c:\masslynx\default.pro\acqudb\5_95mecn_19_min_fa

--------------------- Run method parameters ----------------

Waters Alliance 2695 HPLC Pump Initial Conditions

Solvents
Degasser NormalStroke Volume 130.0 µlFinal Flow Settings being held at End of Run
B% 5.0 MeCN + 0.1% Formic
C% 0.0 Solvent C
D% 0.0 Solvent D
Flow (ml/min) 1.000
Flow Ramp 2.00
Stop Time (mins) 5.0
Column Temperature (°C) 35.0
Column Temperature Limit (°C) 5.0
Min Pressure (Bar) 0.0
Max Pressure (Bar) 300.0
Pre-column Volume (µl) 0.00
Column Position Column 1Pre-column Volume (µl) 0.00

Waters Alliance 2695 HPLC Pump Gradient Timetable

The gradient Timetable contains 6 entries which are :

		Time	A%	B%	C%	D%	Flow	Curve
0.00	95.0	5.0	0.0	0.0	1.000	1		
1.00	95.0	5.0	0.0	0.0	1.000	6		
13.00	0.0	100.0	0.0	0.0	1.000	6		
14.50	0.0	100.0	0.0	0.0	1.000	1		
15.00	95.0	5.0	0.0	0.0	1.000	1		
18.50	95.0	5.0	0.0	0.0	0.100	11		

Waters Alliance 2695 HPLC Pump External Event Timetable

No Entries in the Pump External Event Timetable.

Create PDF files without this message by purchasing novaPDF printer (http://www.novapdf.com)

Waters 2489

Run Time: 14.50 min
Wavelength Mode: Dual Wavelength
Lamp On: On
Channel A...
Comment:
Wavelength: 254 nm
Sampling Rate: 2 points/sec
Data Mode: Absorbance A
Time Constant: 1.0000 sec
Auto Zero On Wavelength Change: Maintain Baseline
Auto Zero On Inject Start: Yes
Channel B...
Comment:
Wavelength: 220 nm
Sampling Rate: 2 points/sec
Data Mode: Absorbance B
Time Constant: 1.0000 sec
Auto Zero On Wavelength Change: Maintain Baseline
Auto Zero On Inject Start: Yes
Analog 1...
Sensitivity: 2.000 AUFS
Chart Polarity: Positive (+)
Voltage Offset: 0 mV
Enable Chart Mark: Yes
Analog 2...
Sensitivity: 2.000 AUFS
Chart Polarity: Positive (+)
Voltage Offset: 0 mV
Enable Chart Mark: Yes
Run Events: Yes
Pulse Width: 1.0 sec
Rect Wave Period: 0.2 sec

Waters Alliance 2695 Autosampler Initial Conditions

Needle Depth (mm)	3.00
Sample Temperature (°C)	20.0
Sample Temperature Limit (°C)	20.0
Purge Loop Volumes	0.00

Sample Run Injection Parameter

Injection Volume (ul) - 10.00

---------------------------- oOo ----------------------------

Create PDF files without this message by purchasing novaPDF printer (http://www.novapdf.com)

End of experimental record.------------------- Generic Instrument Postrun Report ----------

Software Version: 1.30.2065
Firmware Version: 1.40.1966 (Dec 12 2008)
Checksum: 0xe45cd0c3
Serial Number: D1187E056A
Lamp Changed: No
Lamp Life: 20782.00

---------------------------- oOo -----------------------------

Function 1

Scans in function:	1969
Cycle time (secs):	Automatic
Scan duration (secs):	0.200
Inter Scan Delay (secs):	Automatic
Start and End Time(mins):	0.000 to 14.500
Ionization mode:	ES+
Data type:	Enhanced Mass
Function type:	Scan
Mass range:	100 to 1000

Function 2

Scans in function:	1968
Cycle time (secs):	Automatic
Scan duration (secs):	0.200
Inter Scan Delay (secs):	Automatic
Start and End Time(mins):	0.000 to 14.500
Ionization mode:	ES-
Data type:	Enhanced Mass
Function type:	Scan
Mass range:	100 to 1000

Function 3

Scans in function:	1741
Function type:	Diode Array
Wavelength range (nm):	220 to 254

Create PDF files without this message by purchasing novaPDF printer (http://www.novapdf.com)

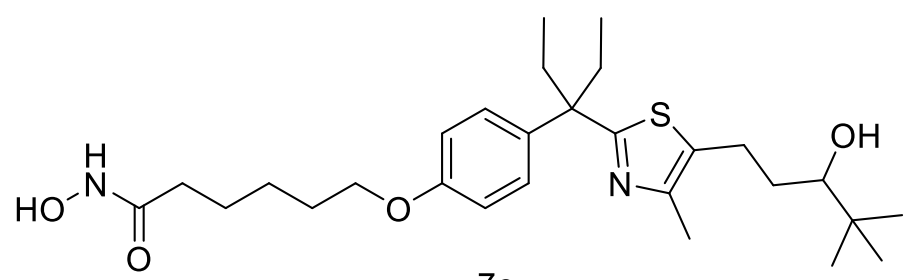

7c

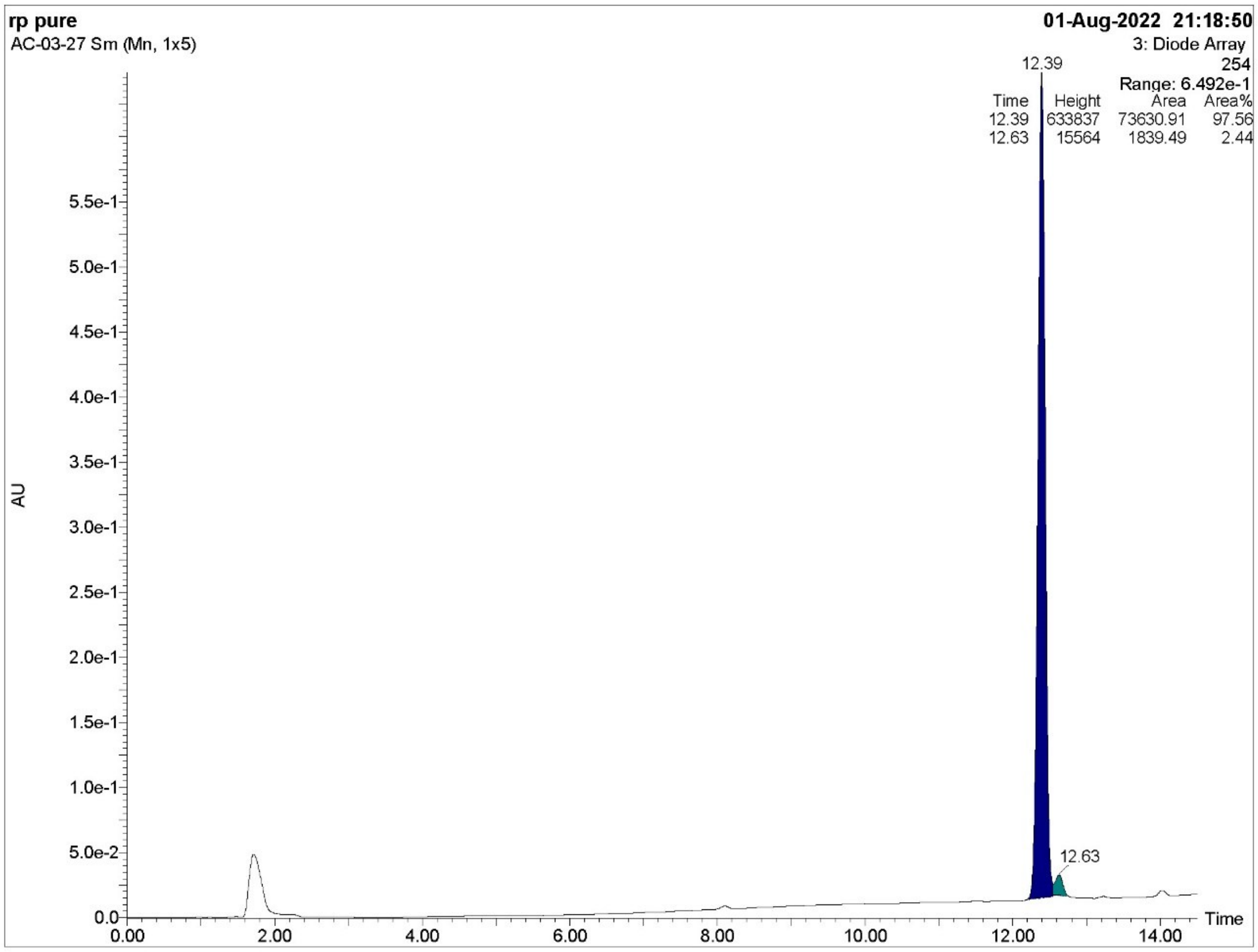

Create PDF files without this message by purchasing novaPDF printer (http://www.novapdf.com)

Bifunctionality of AC-340 in human melanoma cells *in vitro*

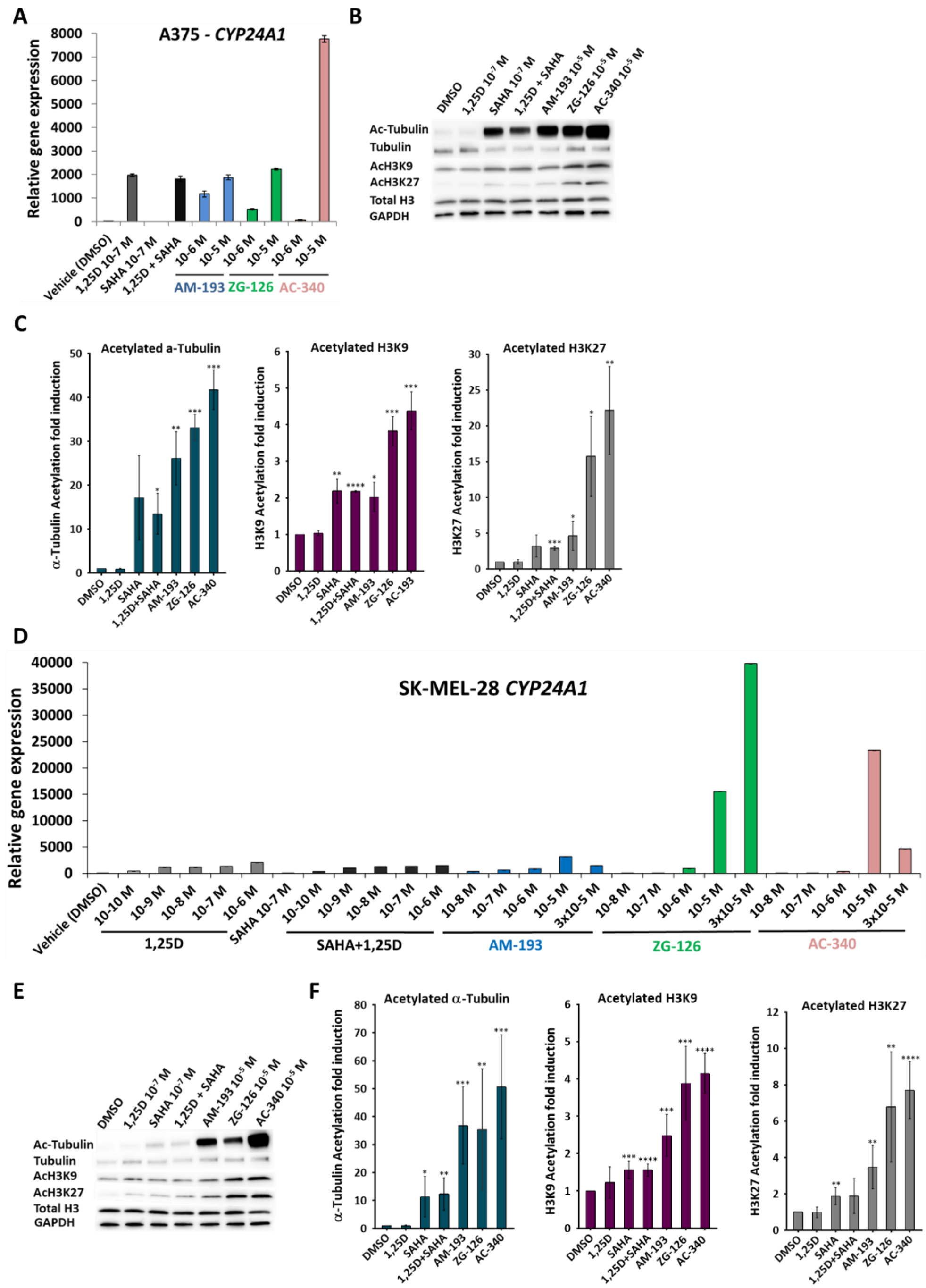

Figure S1. (A) Analysis of *CYP24A1* induction by AC-340, along with 1,25D or SAHA alone or together, AM-193 and ZG-126 in A375 cells. **(B)** Representative western blots showing the level of α-tubulin and histone H3 acetylation in 1,25D-, SAHA- 1,25D+SAHA- or hybrid-treated A375 lysates. **(C)** Fold induction of α-tubulin, H3K9 and H3K27 acetylation quantified from multiple biological replicates. **(D)** Analysis of *CYP24A1* induction by AC-340, along with 1,25D or SAHA alone or together, AM-193 and ZG-126 in SK-MEL-28 cells. **(E)** Representative western blots showing the level of α-tubulin and histone H3 acetylation in 1,25D-, SAHA- 1,25D+SAHA- or hybrid-treated SK-MEL-28 lysates. **(F)** Fold induction of α-tubulin, H3K9 and H3K27 acetylation quantified from multiple biological replicates.

Dose response profiles for inhibition of different HDACs by AC-340

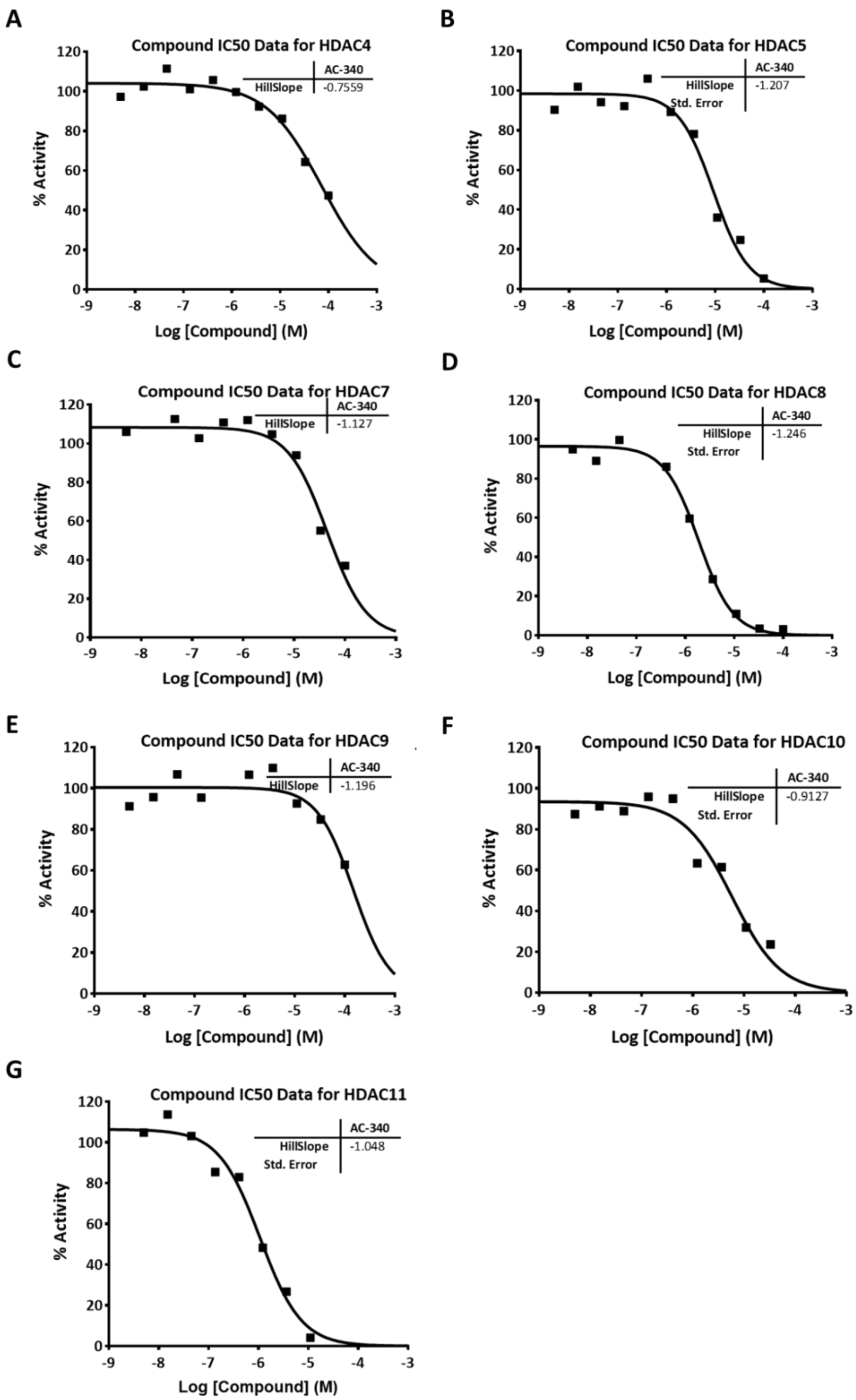

Figure S2. Dose response profiles for inhibition of **(A)** HDAC4**, (B)** HDAC5, **(C)** HDAC7, **(D)** HDAC8, **(E)** HDAC9, **(F)** HDAC10 and **(G)** HDAC11 by AC-340.

RNAseq workflow and volcano plots demonstrating the magnitude of gene regulation in each treatment condition

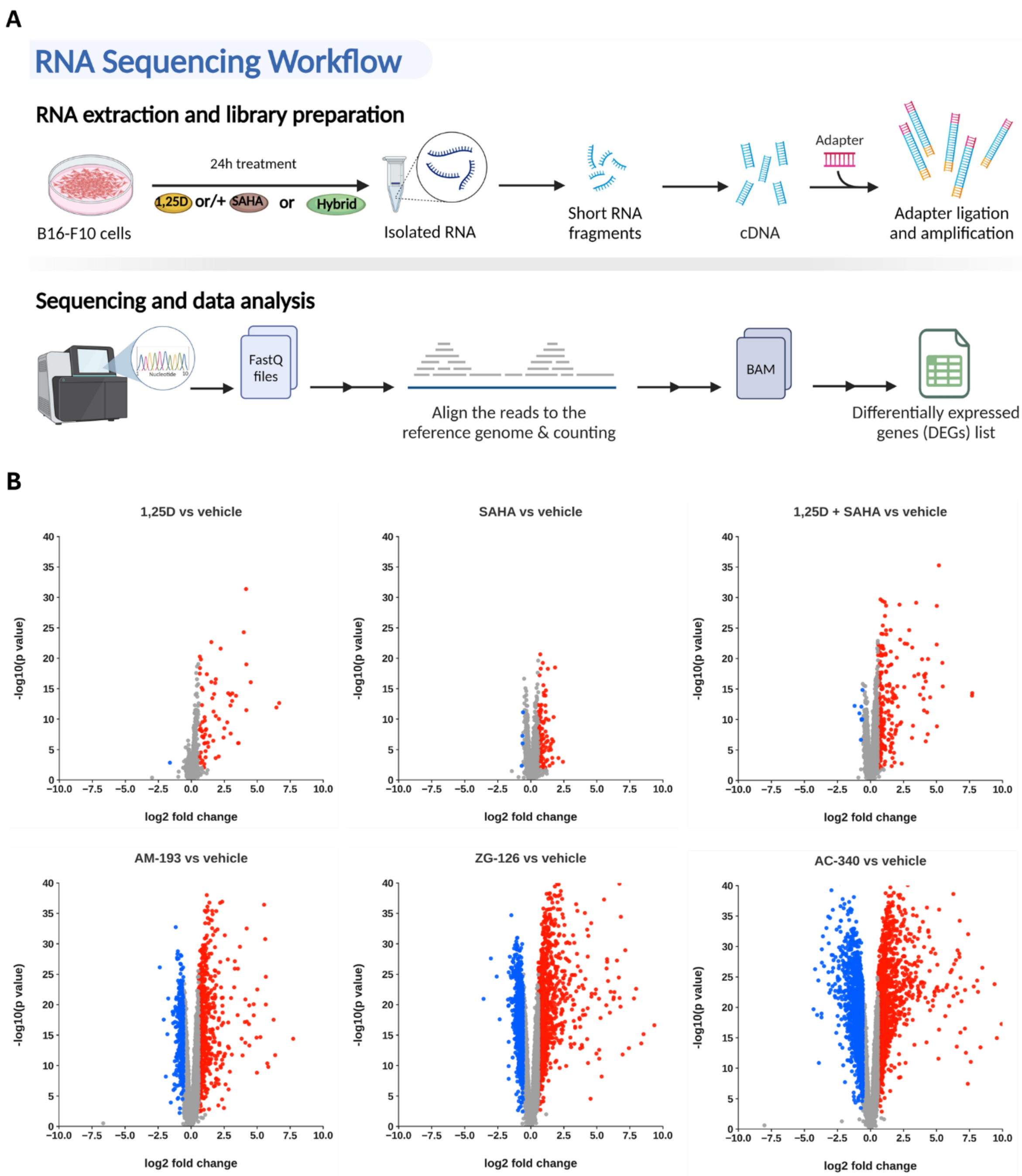

Fig. S3. (A) RNAseq workflow illustrating B16-F10 cells treatment for 24h with DMSO, 1,25D and/or SAHA, AM-193, ZG-126 or AC-340 followed by RNA extraction, library preparation, sequencing and the downstream analyses to discover differentially expressed genes list. **(B)** Volcano plots demonstrating the magnitude of gene regulation in each condition. Significantly upregulated genes were shown in red, whereas significantly downregulated genes were shown in blue. Gray dots represent the genes whose regulation were insignificant.

RNAseq data validation by RT-qPCR of selected VDR target genes in B16-F10 cells

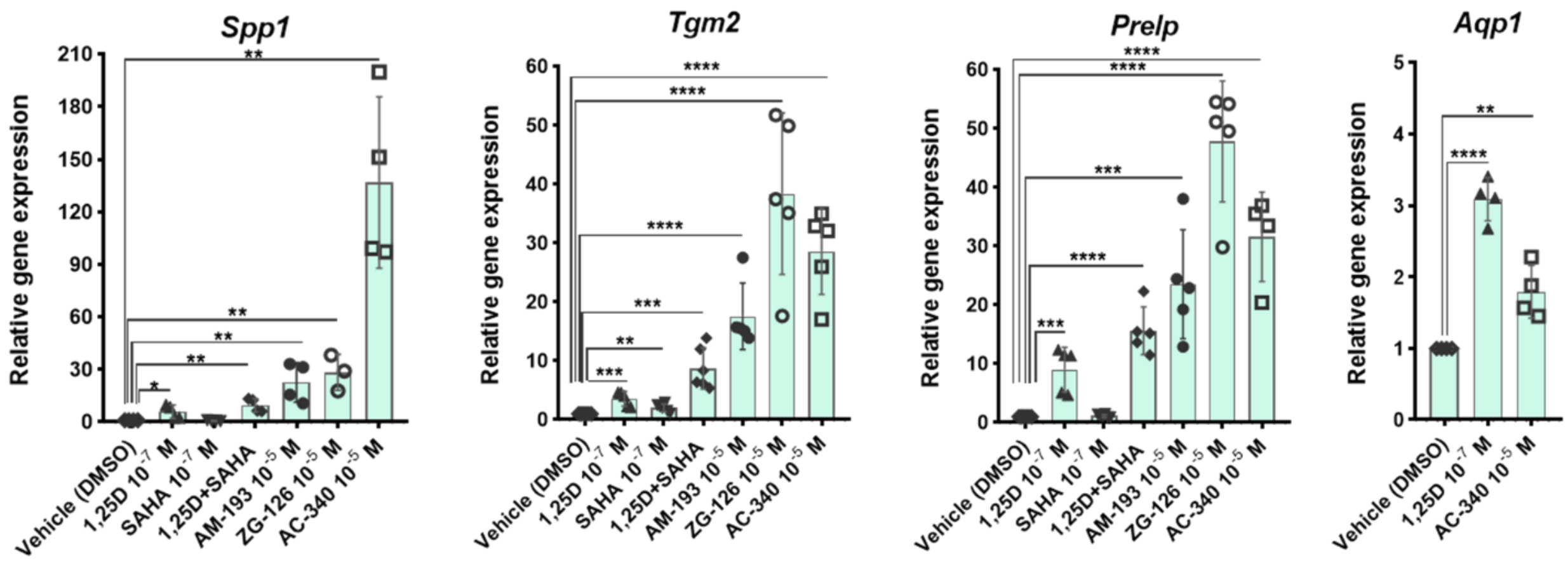

Fig. S4. Validation of the RNAseq data by RT-qPCR. Relative expression of selected VDR target genes in B16-F10 cells treated for 24h with DMSO, 1,25D and/or SAHA, AM-193, ZG-126 or AC-340.

ChIP-qPCR data showing the recruitment of VDR and coactivator CBP to the mouse *Vdr* gene along with the acetylation level of H3K27 in 1,25D- or AC-340-treated B16-F10 cells

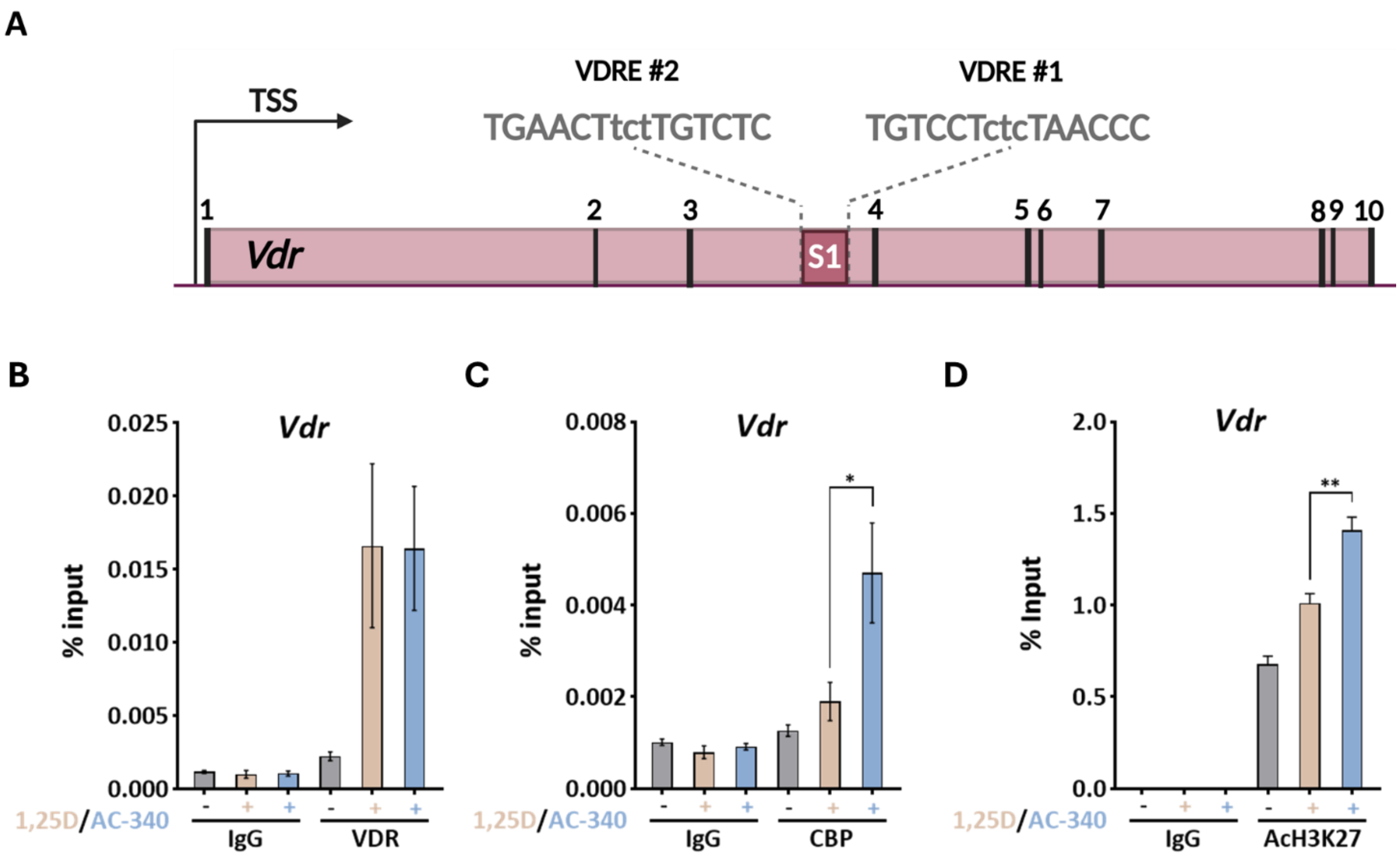

Fig. S5. (A) Schematic diagram of the mouse VDR gene and its 10 exons located on chromosome 15. Intronic region S1 containing two putative VDREs is depicted. **(B)** ChIP-qPCR data showing the recruitment of VDR and **(C)** coactivator CBP to the S1 VDREs, along with **(D)** The acetylation level of H3K27 in B16-F10 cell treated with 1,25D or AC-340 relative to control.

AC-340 modeled into the omit Polder maps

AC-340

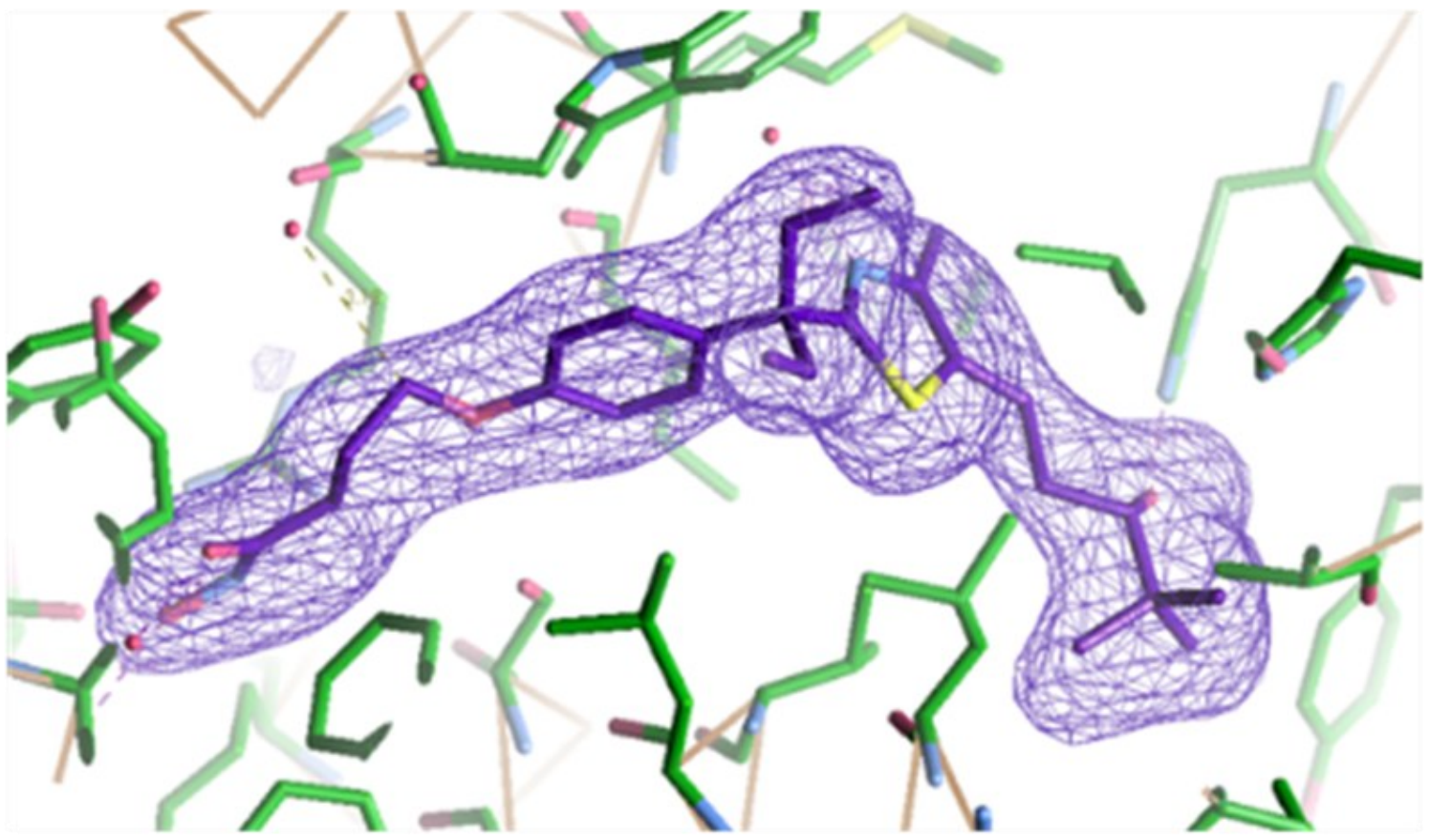

Fig. S6. AC-340 modeled into the omit Polder maps contoured at 3σ.

Protein-ligand distances observed in the AC- or 1,25D-zVDR LBD complexes

AC340

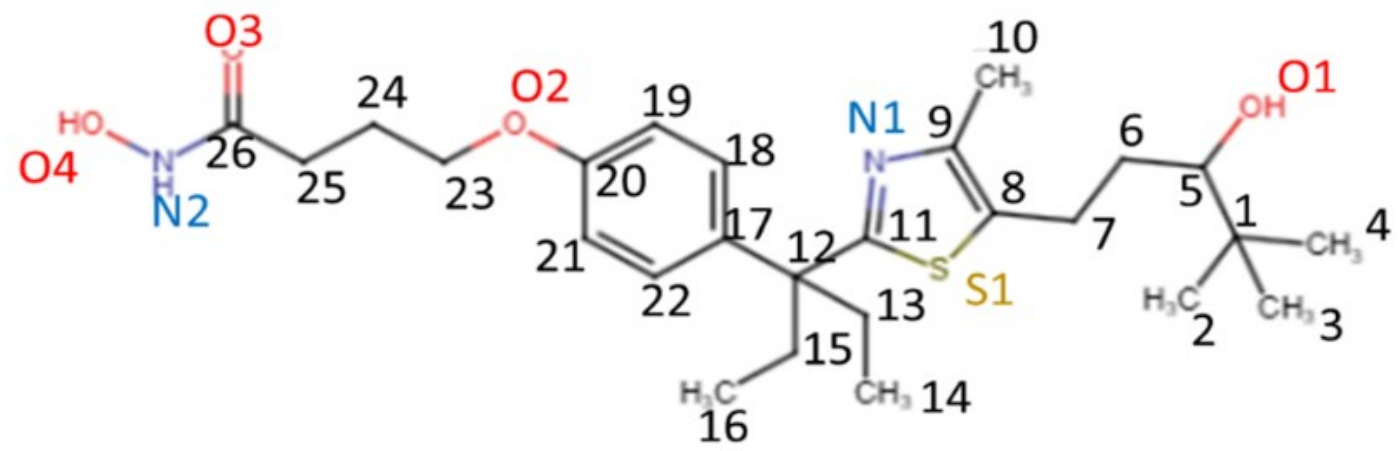

1,25D3 atoms	1,25D$_3$ PDB: 2HC4	AC- atoms	AC-340	1,25D3 atoms	1,25D$_3$ PDB: 2HC4	AC- atoms	AC-340
O1	S265 OG-2.8<br>R302 NH1-2.8	O1	H333 NE2-3.1<br>H423 NE2-2.7	C17	L341 CD1-4.0	C15	W314 CE2-3.5<br>H2O O-3.5
O3	Y175 OH-2.8<br>R302 O-3.9<br>S303 CA-3.3<br>S306 OG-2.8	O2	L261 CD1-3.9<br>S265 OG-3.8<br>H20 O-2.8	C18	V262 CG2-3.8	C16	W314 CH2-3.6<br>V328 CG2-3.4<br>L341 CD2-4.1
C1	S265 OG-3.7<br>R302 CD-4.0<br>S303 CA-4.0	N1	L341 CD2-4.3<br>H2O O-2.6	C20	V328 CG1-4.1	C17	L261 CD2-4.1<br>H2O O-4.0
C2	Y175 CE2-4.0<br>R302 NH1-4.0	S1	V262 CG2-3.8	C21	L337 CD2-3.7<br>H333 CD2-4.0	C18	L261 CD2-4.1<br>I299 CG2-3.9
C3	Y175 OH-3.6<br>Y179 CE2-3.7<br>S306 OG-3.8	C1	H333 NE3-4.2	C22	V262 CG2-4.2	C19	S265 OG-3.9<br>I299 CG2 3.8<br>S303 CB-4.1<br>H2O O-3.5
C4	F182 CZ-4.0<br>L261 CD1-4.2<br>S306 OG-3.7<br>C316 SG-3.5	C2	Y427 CD1-4.0<br>L440 CD2-3.9	C23	H333 NE2-3.7	C20	L261 CD1-4.0<br>S303 CB-3.8<br>H2O O-3.5
C5	S303 CB-3.8	C3	L255 CD1-3.7<br>A331 O-3.4<br>H333 NE2-3.9<br>L430 CD1 3.9	C24	V262 CG2-3.7<br>H423 NE2-4.0	C21	S303 CB-3.6<br>W314 CD1-4.2
C6	L261 CD2-4.2<br>S303 CB-3.5<br>W314 CD1-3.8	C4	L258 O-3.6<br>A259 N-3.7<br>V262 CG2-4.0	C25	H333 NE2-3.7<br>H423 NE2-3.9	C22	S303 CB-3.8<br>W314 CD1-3.8
C7	S303 CB-3.3	C5	H333 NE2-3.3<br>H423 NE2-4.0	C26	L255 CD1-3.8<br>H333 CD2-3.7<br>L430 CD2-3.8	C23	S306 OG-4.1<br>C316 SG-4.2<br>H2O O-3.1
C8	W314 CE2-3.9	C6	V262 CG2-3.4<br>H333 NE2-4.2	C27	Y427 CD1-3.8<br>V444 CG1-4.0	C24	Y175 CE2-4.1<br>F182 CD-4.1<br>H20 O-3.6
C9	W314 CD2-3.4	C7	V262 CG2-4.0<br>H333 NE2-4.1<br>H423 NE2-4.0	O25	H333 NE2-2.8<br>H423 NE2-2.8	C25	S265 OG-3.9<br>R302 NH1-3.5<br>H2O O-3.4
C10	S265 OG-3.8<br>S303 CB-4.1	C8	V262 CG2-4.1			C26	Y175 CE2-4.0<br>Y264 CE2-4.0<br>R302 NH1-3.6
C19	L261 CG-3.6<br>S265 OG-3.1<br>I299 CG2-4.1	C9	H2O O-3.5			O3	Y179 CE1-3.8<br>Y175 CE2-4.0<br>F182 CE1-4.0<br>H2O O 2.1
C11	L258 CD2-4.0<br>Y323 CB-4.0	C10	I296 CD1-3.7<br>M300 CE-3.7<br>H2O O-3.5			O4	T174 O-3.4<br>Y175 CA-3.5<br>D176 N-3.5<br>Y264 CE2-3.6<br>K268 CG-3.9<br>R302 NH2-3.7<br>H2O O-2.1
C12	V328 CG2-3.5	C11	H2O O-3.6			N2	T174 O-4.3<br>Y175 CD2-4.0<br>Y264 CE2-3.6<br>R302 NH2-3.6<br>H2O O-3.6
C14	W314 CE2-4.3	C12	H2O O-3.9				
C15	I299 CG2-4.0	C13	L258 CD2-4.0<br>L261 CD2-3.9				
C16	M300 CG-4.3<br>L341 CD1-4.0	C14	L258 CD2-4.0<br>L261 CD2-4.0<br>Y323 CD2-3.4				

Table S1. Protein-ligand distances (in Å) observed in the zVDR LBD-1,25D, AC- analogs complexes. A cutoff of 4.0 Å has been used. For comparison purposes, contacts greater than 4.0 Å are indicated in italics. When multiple contacts are formed between a ligand atom and one residue, only the strongest one is indicated.

Crystallographic data and refinement

	zVDR LBD - AC-340
PDB ID	9RCG
Data collection	
Space group	$P6_522$
Unit-cell parameters (Å, °)	66.099, 66.099, 263.593<br>90, 90, 120
Resolution range (Å)	47.97-2.15
Unique reflections	19542
$I/\sigma(I)$	25.7
$CC_{1/2}$	1
Completeness	100
Multiplicity	2.0
Refinement	
Resolution range (Å)	22.02-2.10
R_{work} / R_{free} (%)	20.89 / 18.18
Ramachandran	
Allowed (%)	0.82
Favored (%)	99.18
Rmsd	
Bond (Å)	0.002
Angle (°)	0.48
B factors	
Protein	59.25
Ligand	48.13
Water	66.5

Table S2. Crystallographic data and refinement.

Primers used for RT-qPCR and ChIP-qPCR

Gene	Forward sequence	Reverse sequence	Application
Cyp24a1	5'-GCGAGCTGAACAAATGGTCC-3'	5'-CTTTGGTGTTGAGGCGCTTG-3'	RT-qPCR
Vdr	5'-ACGCTATGACCTGTGAAGGC-3'	5'-TTACGCTGCACCTCCTCATC-3'	RT-qPCR
Spp1	5'-TCCTTGCTTGGGTTTGCAGT-3'	5'-GTGGCTATAGGATCTGGGTGC-3'	RT-qPCR
Tgm2	5'-CCACTTTCCGATCCCCTGTA-3'	5'-GAGGCCAATATCAGTCGGGA-3'	RT-qPCR
Prelp	5'-GAACAGAAGAGTGCCCCAGA-3'	5'-CCAGGTTTTGGTCTTGTCGG-3'	RT-qPCR
Aqp1	5'-GAGCCCTGTCTGCATCCATC-3'	5'-GGTGCTCAACTCCCTCCAAG-3'	RT-qPCR
18s	5'-GCAATTATTCCCCATGAACG-3'	5'-GGGACTTAATCAACGCAAGC-3'	RT-qPCR
CYP24A1	5'- CTACCGCAAAGAAGGCTACG-3'	5'- TTGGTGTTGAGGCTCTTGTG-3'	RT-qPCR
VDR	5'-ACCTACGACCCCACCTACTC-3'	5'-GGAGTGTGTCTGGAGTTGGG-3'	RT-qPCR
18S	5'- CGGCTACCACATCCAAGGAA-3'	5'- GCTGGAATTACCGCGGCT-3'	RT-qPCR
Spp1	5'-ACCTCTTCTGCTCTATATGGC-3'	5'-AATTCCTCTCCTGCCTCCA-3'	ChIP-qPCR
Vdr	5'-CCCCTGGCACTTTATGTTTTT-3'	5'-AACCCTGTCATTCTCTAACTGG-3'	ChIP-qPCR

Table S3. Primer sequences for RT-qPCR and ChIP-qPCR.

Antibodies used for western blots and ChIP assays

Antibody	Company	Catalog number	Application
Acetylated tubulin	Sigma Aldrich	T7451	Western Blot
Tubulin	Sigma Aldrich	T9026	Western Blot
Acetyl-histone H3(Lys9)	Merck Millipore	07-352	Western Blot
Acetyl-histone H3 (Lys27)	Abcam	ab4729	Western Blot
Histone H3	Cell Signaling Technology	3638	Western Blot
GAPDH	Abcam	ab8245	Western Blot
VDR	Santa Cruz Biotechnology	sc-13133	Western Blot and ChIP
anti-mouse IgG HRP-linked	Cell Signaling Technology	7076	Western Blot
anti-rabbit IgG HRP-linked	Cell Signaling Technology	7074	Western Blot
CBP	Invitrogen	MA5-13634	ChIP
Acetyl-histone H3 (Lys27)	Cell Signaling Technology	8173	ChIP
Normal rabbit IgG	Cell Signaling Technology	2729	ChIP

Table S4. Antibodies used for Western Blot and ChIP assay.
